# SAG1.3-derived Frizzled-targeting small-molecule compounds

**DOI:** 10.1016/j.jbc.2025.110751

**Published:** 2025-09-22

**Authors:** Lukas Grätz, Ainoleena Turku, Pawel Kozielewicz, Carl-Fredrik Bowin, Magdalena M. Scharf, Jan H. Voss, Julia Kinsolving, Rawan Shekhani, Nuria Oliva-Vilarnau, Tobias Koolmeister, Marlies Körber, Volker M. Lauschke, Stefan Löber, Peter Gmeiner, Gunnar Schulte

**Affiliations:** 1 Karolinska Institutet, Department Physiology & Pharmacology, Sec. Receptor Biology & Signaling, Biomedicum, Stockholm, Sweden; 2 Karolinska Institutet, Department Physiology & Pharmacology, Sec. Personalized Medicine and Drug Development, Biomedicum, Stockholm, Sweden; 3 Chemical Biology Consortium Sweden, Science for Life Laboratory, Department of Medical Biochemistry and Biophysics, Karolinska Institutet, Stockholm, Sweden; 4 Department of Chemistry and Pharmacy, Friedrich-Alexander-Universität Erlangen-Nürnberg, Erlangen, Germany; 5 Dr Margarete Fischer-Bosch Institute of Clinical Pharmacology, Translational Pharmacology and Drug Discovery, Stuttgart, Germany; 6 University of Tübingen, Tübingen, Germany; 7 FAUNeW – Research Center New Bioactive Compounds, Friedrich-Alexander-Universität Erlangen-Nürnberg, Erlangen, Germany

**Keywords:** G protein–coupled receptor, molecular pharmacology, medicinal chemistry, drug discovery, biosensor, bioluminescence resonance energy transfer, signal transduction, Wnt pathway

## Abstract

Exaggerated Wingless/Int1 (WNT)–Frizzled (FZD) signaling contributes to pathologies, including fibrosis and different forms of cancer. Thus, targeting FZDs as WNT receptors for therapeutic purposes constitutes a promising intervention if the imminent risk of unwanted side effects caused by the involvement of WNT–FZD signaling in stem cell regulation and tissue homeostasis can be controlled. Here, we derivatize SAG1.3 (SMO agonist), which acts through FZD_6_ as a partial agonist. Screening of SAG1.3 derivatives identified compound 11 that competed with BODIPY (boron–dipyrromethene)–cyclopamine binding at different FZDs and inhibited WNT-induced FZD dynamics and β-catenin signaling in human embryonic kidney 293 (HEK293) cells. Furthermore, compound 11 blocked WNT-3A-induced *LGR5* gene expression in human primary hepatocyte spheroids and reduced the viability of RNF43-mutated but not RNF43-wildtype pancreatic cancer cells. Based on our data, we suggest that compound 11 acts on FZDs to limit WNT- and WNT-surrogate–induced receptor dynamics, providing a valid proof of concept for targeting FZDs with small-molecule compounds.

The class Frizzled (class F) of G protein–coupled receptors (GPCRs) consists of 11 receptors (1, 2). One of these receptors, Smoothened (SMO), mediates Hedgehog signaling and has already been targeted by an elaborate set of small-molecule compounds, some of which are in clinical use for the treatment of basal cell carcinoma (3). The other 10 Frizzleds (FZD_1–10_) bind the Wingless/Int-1 (WNT) family of secreted lipoglycoproteins *via* their extracellular cysteine-rich domain (CRD), which thereby represents by definition the orthosteric binding site of FZDs (4). WNT–FZD signaling plays an essential role in embryonic development, stem cell regulation, tissue homeostasis, and its perturbation. Imbalanced WNT–FZD signaling can result in diverse pathologies, including various developmental disorders, fibrosis, and cancer (5), making the WNT–FZD signaling system a promising therapeutic target (6, 7, 8, 9, 10).

While some types of cancer, such as colon cancer, are often driven by WNT pathway mutations in genes encoding for proteins acting downstream of FZDs, such as mutations in adenomatous polyposis coli and β-catenin, other cancer types are associated with high expression levels of WNT proteins and increased total or surface expression of FZDs (6, 10, 11). Therefore, it appears most suitable in the context of antitumor therapy to inhibit exaggerated WNT–FZD-mediated cell communication by developing inverse agonists, antagonists, or negative allosteric modulators into potential therapeutically meaningful concepts. Addressing cell surface receptors pharmacologically allows targeting signaling with higher precision, especially when compared with modulation of downstream cascades that are often pleiotropically involved in complex signaling networks. In order to directly target FZDs, several strategies seem to be feasible. On one hand, the route of targeting the orthosteric WNT binding site on the CRD by biologics such as antagonistic antibodies as well as bifunctional agonistic WNT mimetics, the so-called WNT surrogates, has been pursued (12, 13, 14, 15). The CRD can also be targeted by small molecules exemplified by carbamazepine, which is surmised to block WNT-induced signaling at FZD_8_ (16). On the other hand, following the more classical GPCR strategy, the seven transmembrane core of FZDs offers diverse opportunities for drug targeting. Despite the claim that the core of FZDs could be undruggable (17), molecules can indeed target the FZD receptor core, as illustrated by the SMO agonist SAG1.3 that acts as a weak partial agonist at FZD_6_ (18). Also, other supposedly core-targeting compounds have been identified by *in silico* structure–based virtual screening (19), and allosteric modulators acting at intracellular sites have been reported as folding chaperones with pharmacological activity (20, 21). However, it must be underlined that most of the aforementioned compounds did not withstand an independent validation of the proposed pharmacological mode of action of compound-induced inhibition of WNT-induced and FZD-mediated downstream signaling along the WNT–β-catenin signaling axis (22, 23). Clearly, these recent discoveries underline how difficult it is to reliably target these receptors.

Here, building on our previous efforts, we employ a strategy guided by a receptor model and systematic lead compound modifications to derivatize SAG1.3 aiming at the discovery of receptor core–targeting small molecules for FZDs. Competition binding experiments at FZD_6_ enabled us to identify DJ503701 (compound 11) as the compound with the highest affinity in our set. Further validation defined compound 11 as a small-molecule modulator acting *via* FZDs in a paralog-nonselective manner, as it efficiently inhibited WNT-induced and FZD-mediated signaling in several diverse contexts, including in human embryonic kidney 293 (HEK293) cells and patient-derived primary human liver spheroids. Furthermore, compound 11 reduced the viability of WNT signaling–dependent human pancreatic ductal adenocarcinoma cells (PDACs). Thus, our findings demonstrate that FZDs indeed are druggable by small molecules, which can elicit therapy-relevant effects.

## Results

### Designing SAG1.3 analogs and ligand competition binding at FZD_6_

**Figure 1:**
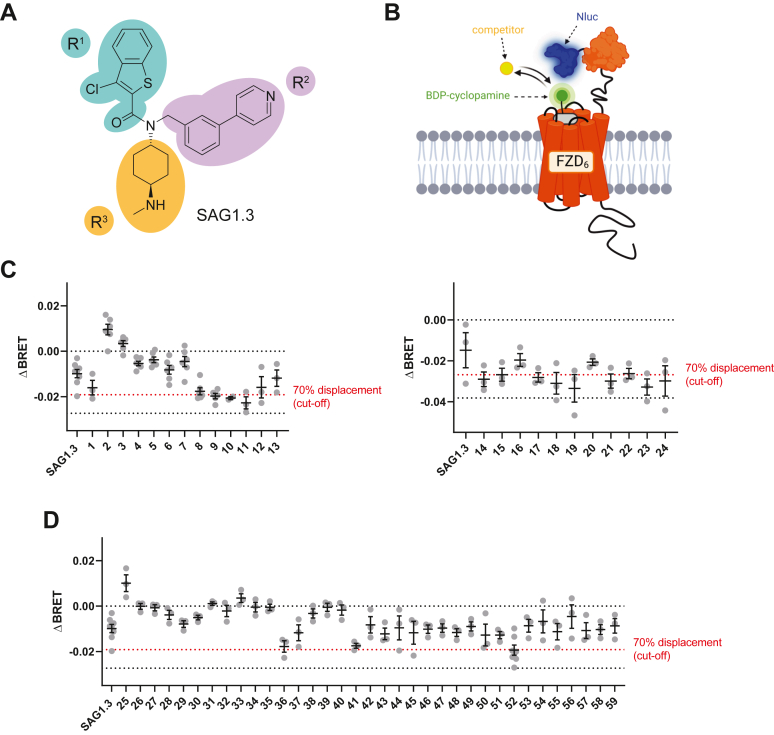
**Rationale of the study and screening of SAG1.3 derivatives for binding to Nluc-FZD_6_**. *A,* chemical structure of SAG1.3. The molecule is divided into three regions for subsequent chemical modifications (R^1^–R^3^). *B*, schematic of the BRET-based competition binding assay at Nluc-FZD_6_ (tracer: BODIPY [BDP]–cyclopamine) used for screening the generated SAG1.3 derivatives. Created with biorender.com. *C* and *D*, screening results for SAG1.3 derivatives 1 to 59 (10 μM) from BRET-based competition binding assays with BODIPY–cyclopamine (c = 300 nM) performed in ΔSMO HEK293A or ΔFZD_1–10_ HEK293T cells transiently transfected with Nluc-FZD_6_. Compounds from the first round of modifications focusing on R^3^ (1–24) are shown in (*C*), whereas compounds from the second set (25–59) are shown in (*D*). The *lower black dashed line* indicates baseline levels (“donor-only” conditions) indicative of full tracer displacement. The *red dashed line* indicates a predefined cutoff value (70% tracer displacement), which was used as a decision criterion for further characterization. Note that the absolute ΔBRET values for full displacement in the *right panel* of (*C*) are different, as another plate reader was used for data acquisition. Data in (*C* and *D*) represent mean values ± SEM from two to eight independent experiments, each performed in duplicate. BODIPY, boron–dipyrromethene; BRET, bioluminescence resonance energy transfer; FZD, Frizzled; HEK293, human embryonic kidney 293 cell line.

We recently showed that SAG1.3 (Fig. 1*A*) acts on FZD_6_ as a weak partial agonist by targeting the transmembrane receptor core (18, 24). However, SAG1.3 acts at FZD_6_ with double-digit micromolar potency and very low efficacy, rendering it a relatively poor tool compound for studying FZD pharmacology effectively. Thus, we aimed at using the information about the binding site for the design of more potent FZD-targeting ligands. To do so, we divided SAG1.3 into three regions, which we called R^1^–R^3^ (Fig. 1*A*). Initially, we designed and synthesized a small number of SAG1.3 analogs (1–24), focusing on modifications of the R^3^ region (Fig. 1*A*) given that our receptor model suggested the availability of an aromatic subpocket able to accommodate such compounds (Fig. S1). The compounds were tested at a concentration of 10 μM in a nanobioluminescence resonance energy transfer (BRET)–based competition binding assay at Nluc-FZD_6_ employing the tracer compound boron–dipyrromethene (BODIPY)–cyclopamine (Fig. 1*B*), which we have previously used to assess FZD_6_ binding in live HEK293 cells (18, 24). The chemical structures of all generated SAG1.3 analogs are shown in Table S1. While some of the compounds were only weakly or not at all interfering with BODIPY–cyclopamine binding, others represented a significant improvement over the parent compound SAG1.3, almost reaching baseline levels (“donor-only” control) at a concentration of 10 μM (Fig. 1*C*, *black dashed line*). Building upon these first promising results, we set out to investigate the other two regions in SAG1.3 (R^1^ and R^2^), which had not been the focus in the first compound set. The design of the majority of analogs modified at R^1^–R^3^ was then based on exploring the chemical space, which could be cost-effectively assessed both from an economical and chemical–synthetical perspective (compounds 25–59). However, out of this second compound set, only a few showed better properties than the parental compound SAG1.3 (Fig. 1*D*). Few compounds tested showed an increase in BRET (Fig. 1, *C* and *D*). While this most likely originates from optical interference, it could theoretically be evidence for a positive allosteric regulation of tracer binding. At this point, we have not followed up on this phenomenon.

### Compound 11 binds nonselectively to the receptor core of FZDs

**Figure 2:**
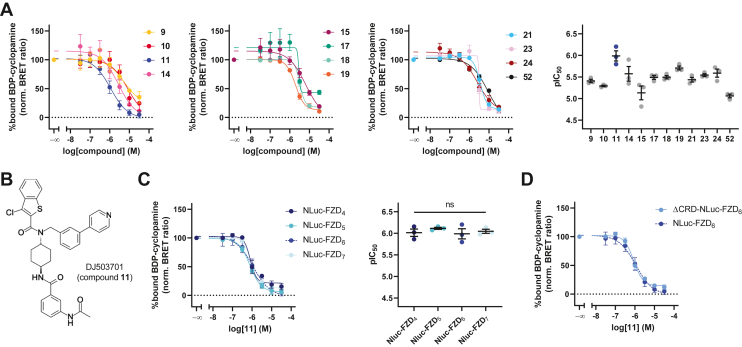
**Validation of selected compounds in BRET-based competition binding experiments.***A*, displacement curves and pIC_50_ values from BRET-based competition binding experiments at Nluc-FZD_6_ with selected SAG1.3 derivatives and BODIPY–cyclopamine (c = 200 nM). Experiments were performed in ΔFZD_1–10_ HEK293T cells transiently transfected with Nluc-FZD_6_. *B*, chemical structure of DJ503701 (compound 11). *C* and *D*, displacement curves and corresponding pIC_50_ values (only in (*C*)) from BRET-based competition binding experiments with compound 11 and BODIPY–cyclopamine (c = 200 nM) performed in ΔFZD_1–10_ HEK293T cells transiently transfected with Nluc-FZD_4_, Nluc-FZD_5_, or Nluc-FZD_7_ (*C*) or ΔCRD-Nluc-FZD_6_ (*D*). Note that data for Nluc-FZD_6_ (*dashed blue lines*) in (*C* and *D*) were copied from (*A*) for illustration purposes. Experimental data in (*A*, *C*, and *D*) represent mean values ± SEM from three independent experiments performed in triplicate. Statistical significance in (*C*) was assessed using one-way ANOVA followed by Tukey’s *post hoc* test. BODIPY, boron–dipyrromethene; BRET, bioluminescence resonance energy transfer; FZD, Frizzled; HEK293, human embryonic kidney 293 cell line; ns, not significant.

Based on these screening results, 12 compounds were selected (cutoff value: 70% reduction of BODIPY–cyclopamine binding; Fig. 1 and Table S1) for full concentration–response curves in our competition binding assay setup. Interestingly, most of these compounds showed a sterically demanding substituent at the secondary amine. In particular, arylamide derivatives revealed very promising data. Confirming our screening results, all tested compounds were able to reduce tracer binding to Nluc-FZD_6_ in a concentration-dependent manner (Fig. 2*A*). The set of compounds displayed IC_50_ values in the low micromolar to high nanomolar range. DJ503701 (compound 11; Fig. 2*B*) showed the highest pIC_50_ value (Figs. 2*A* and Table S2) and was therefore chosen for a more in-depth characterization. We performed analogous BODIPY–cyclopamine competition binding experiments with one member of every FZD homology cluster, employing ΔFZD_1–10_ HEK293 cells transiently transfected with N-terminally Nluc-tagged versions of either FZD_4_, FZD_5_, or FZD_7_ to determine paralog selectivity. Compound 11 showed a similar ability to reduce BODIPY–cyclopamine binding from any of these receptors (Fig. 2*C* and Table S3), suggesting that compound 11 is not selective within the FZD family. Competition binding experiments with a FZD_6_ construct lacking the CRD (ΔCRD-Nluc-FZD_6_) further support the notion—similar to what was observed for SAG1.3—that compound 11 targets the transmembrane core of FZDs and not the orthosteric WNT binding site on the CRD (Fig. 2*D*).

### Assessment of the mode of action of compound 11

**Figure 3:**
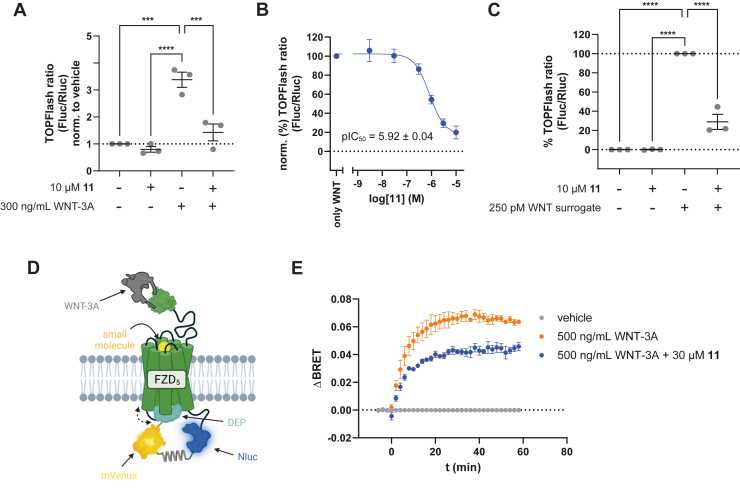
**Functional characterization of compound 11**. *A*, TOPFlash reporter gene response induced by vehicle control, compound 11, WNT-3A or a combination of compound 11 and WNT-3A in ΔFZD_1–10_ HEK293T cells transiently transfected with HiBiT-FZD_5_. *B*, concentration–response curve of compound 11 inhibiting the TOPFlash response induced by 300 ng/ml WNT-3A. Experiments were performed in ΔFZD_1–10_ HEK293T cells transiently transfected with HiBiT-FZD_5_. *C*, TOPFlash reporter gene response induced by vehicle control, compound 11, WNT surrogate, or a combination of compound 11 and WNT surrogate in ΔFZD_1–10_ HEK293T cells transiently transfected with HiBiT-FZD_5_. Vehicle response was set to 0%, and WNT surrogate response was normalized to 100%. *D*, schematic depiction of the FZD_5_–DEP–Clamp assay setup. Created with biorender.com. *E*, the kinetic ΔBRET response of the FZD_5_–DEP–Clamp sensor recorded upon addition of vehicle control, 500 ng/ml high-purity WNT-3A, or 500 ng/ml high-purity WNT-3A together with 30 μM of compound 11. Experiments were performed in HEK293A cells stably expressing the FZD_5_–DEP–Clamp sensor. All experimental data (*A*, *B*, *C*, and *E*) represent mean values ± SEM from three independent experiments, each performed in triplicate. Statistical significance in (*A* and *C*) was assessed using one-way ANOVA followed by Dunnett’s *post hoc* test. ns, not significant; ∗*p* < 0.05; ∗∗∗*p* < 0.001. DEP, Dishevelled, Egl-10, and Pleckstrin; FZD, Frizzled; HEK293, human embryonic kidney 293 cell line; WNT, Wingless/Int1.

While SAG1.3 showed weak positive efficacy on FZD_6_ with regard to conformational changes in the receptor core, miniG protein recruitment, heterotrimeric G_i_ protein activation, and extracellular signal–regulated kinase 1/2 phosphorylation, it remained unclear whether the positive efficacy of the parent compound was maintained through the chemical modification of the R(1–3) moiety (18). The ability of compound 11 to bind FZD paralogs nonselectively opened for a rich arsenal of functional assays to define the compound’s mode of action. For this functional characterization, we decided to focus on human FZD_5_, a receptor that mediates WNT-induced CRD dynamics, receptor core conformational changes, FZD–Dishevelled (DVL) interface dynamics, WNT–β-catenin signaling, and the activation of heterotrimeric G proteins (18, 24, 25, 26, 27, 28). In ΔFZD_1–10_ HEK293T cells transiently transfected with human FZD_5_, addition of 300 ng/ml recombinant WNT-3A elicited a substantial TOPFlash response indicative of activation of the WNT–β-catenin pathway (Fig. 3*A*). Compound 11 efficiently counteracted this WNT-3A-induced effect in a concentration-dependent manner with a pIC_50_ value of 5.92 ± 0.04 (equal to an IC_50_ value of 1.20 μM; Fig. 3*B*) while not inducing any effect in the absence of added WNT-3A (Fig. 3*A*). In agreement with the BODIPY–cyclopamine competition binding experiments, compound 11 did not show a subtype preference in TOPFlash assays (Fig. S3). In this context, it must be underlined that small-molecule compounds can interfere with the experimental readout and that counter assays are instrumental to define the compound’s mode of action. An important tale of caution was recently presented by us and others with a surmised FZD_7_ inhibitor that turned out to mediate its effects solely through firefly luciferase (Fluc) inhibition (22, 23). Also, compound 11 interferes with Fluc bioluminescence (Fig. S3*D*). However, the extent of signal reduction in WNT-induced TOPFlash was much higher than for Fluc alone. Thus, the interference of compound 11 with Fluc still allows conclusions regarding the pharmacological efficacy of the compound in Fluc-dependent assay formats such as the TOPFlash readout. In order to further support the FZD selectivity of compound 11, we have used an unrelated class A GPCR (muscarinic acetylcholine M_1_ receptor) in connection with a BRET-based mini-Gsq protein recruitment assay (29). Compound 11 neither elicited a mini-Gsq recruitment on its own nor affected the concentration-dependent M_1_-mediated mini-Gsq response upon the addition of carbachol (Fig. S3*E*).

Initiation of WNT–β-catenin signaling is typically governed by WNT-mediated association of the extracellular regions of both FZD and the coreceptor LRP5/6 and the formation of a so-called signalosome. Signalosome formation can also be induced by artificial biologics called WNT surrogates carrying binding moieties for both entities (13). Along these lines, the addition of WNT surrogate to ΔFZD_1–10_ HEK293T cells transiently transfected with human FZD_5_ led to a significant increase in WNT–β-catenin signaling (Fig. 3*C*). Notably, the WNT surrogate–induced signal, only induced by crosslinking the extracellular moieties, could also be blocked by coaddition of the small-molecule compound 11 (Fig. 3*C*).

The phosphoprotein DVL is at the crossroads of both β-catenin-dependent and -independent WNT signaling, and DVL interacts with FZDs mainly through its Dishevelled, Egl-10, and Pleckstrin (DEP) domain (26, 30, 31, 32). We have recently designed a genetically encoded biosensor, which we called FZD_5_–DEP–Clamp (for schematic depiction, see Fig. 3*D*) (26), selectively reporting on the WNT-induced FZD_5_–DEP interface dynamics. WNT stimulation of the FZD_5_–DEP–Clamp sensor equipped with an intramolecular BRET donor–acceptor pair displayed a robust increase in BRET (Fig. 3*E*) (26). In contrast, compound 11 alone did not elicit a robust change in BRET response (Fig. S4*A*). However, in analogy to our findings in the TOPFlash reporter gene assay, compound 11 was able to interfere with the WNT-induced effects on the FZD_5_–DEP–Clamp, suggesting an information flow from the extracellular WNT binding site to the intracellular FZD–DEP–DVL interface, which can be modulated by small molecules binding to the receptor core in an allosteric manner.

Moreover, we controlled for the effect of compound 11 on receptor cell surface expression, which could be negatively affected by a compound destabilizing the receptor, thereby leading to reduced cell surface trafficking. Overnight exposure to compound 11 or compound 26, which is structurally related to compound 11 but did not compete with BODIPY–cyclopamine binding (Fig. S4*B* and *C*), had no effect on the surface levels of HiBiT-FZD_5_ and HiBiT-FZD_6_. Thereby, we exclude the risk that compound 11 exerts its inhibitory effect on WNT-induced and FZD-mediated effects by affecting receptor stability or trafficking.

Taken together, even though compound 11 most likely binds to the receptor core and thus to a site distinct from the orthosteric WNT binding site, the compound is still capable of impacting the signaling output induced by WNT. Therefore, we suggest that compound 11 acts as a negative allosteric modulator of WNT–FZD-mediated signaling.

### Compound 11 interferes with WNT-driven signaling in hepatocyte spheroids

Next, we aimed to evaluate whether and how compound 11 would also impact signaling in a physiologically relevant, nonrecombinant system. To this end, we used organotypic 3D cultures of primary human hepatocytes (PHHs) in which the cultured cells retain their transcriptomic, proteomic, and metabolomic phenotype for multiple weeks (33, 34, 35).

**Figure 4:**
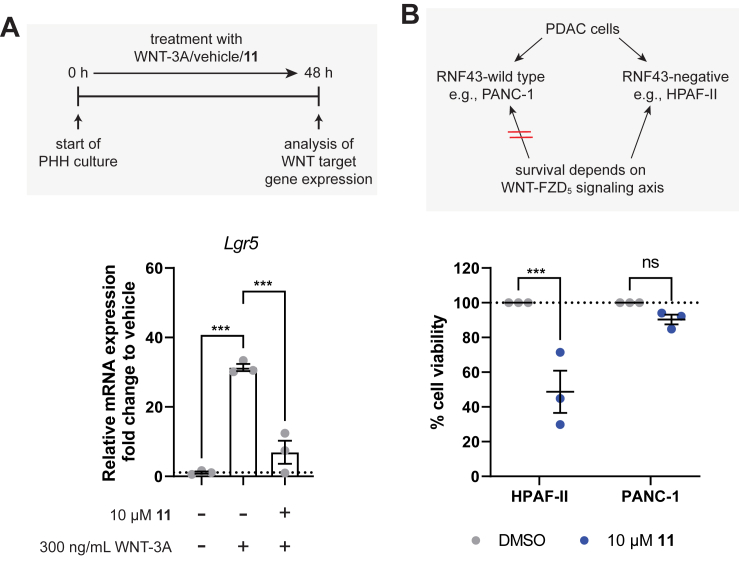
**Effect of compound 11 on WNT-induced signaling in primary hepatocyte spheroids and pancreatic cancer cells**. *A*, the effect of compound 11 on WNT-3A-induced gene expression (*LGR5*) in primary human hepatocyte (PHH)–derived spheroids. Data shown are mean values ± SEM from three independent experiments. A representative micrograph of a PHH spheroid is shown in Fig. S5*A*. *B*, the effect of compound 11 or vehicle control on the viability of RNF43-negative (HPAF-II) and RNF43-wildtype (PANC-1) pancreatic ductal adenocarcinoma (PDAC) cells. Data represent normalized mean values ± SEM from three independent experiments performed in triplicate. Statistical significance in (*A*) was assessed using a one-way ANOVA followed by Tukey’s *post hoc* test, whereas for (*B*), a two-way ANOVA followed by Šidak’s *post hoc* test was used. ns, not significant, ∗∗∗*p* < 0.001. WNT, Wingless/Int1.

The liver is the organ with the highest regenerative capacity in the human body, for which functional WNT–β-catenin signaling, among other pathways, is a critical cue (36). To assess the effects of compound 11, we generated 3D spheroids from PHHs and monitored expression changes of the prototypical WNT–β-catenin target gene *LGR5* upon addition of WNT alone or together with the non–FZD-selective compound 11. In agreement with previous reports, the addition of WNT-3A increased the mRNA levels of *LGR5* (Fig. 4*A*) (37). Upon coaddition of compound 11, a reduction of the WNT-induced increase in expression could be detected for the target gene, returning mRNA expression almost back to vehicle levels. This observation confirmed that compound 11 interferes with WNT–β-catenin signaling also in more complex and physiologically relevant test systems.

### Compound 11 reduces viability of RNF43-negative pancreatic cancer cells

The development of small molecules directly inhibiting FZD function represents an interesting yet unexploited avenue for drug discovery and therapy, even though some improvements have been made. Inhibition of FZDs could have a therapeutical benefit for tumor subtypes that are driven by either overexpression of WNTs, activating mutations of FZDs, or enhanced cell surface expression of FZDs. In human PDAC, about 7% of the patients present with impairing mutations in the E3 ubiquitin ligase RNF43, which was described to reduce surface levels of FZDs (38). The interrupted inhibitory regulation by RNF43 of FZD surface expression results in an enhanced autocrine WNT-dependent signaling loop (10, 39). It was further shown that disruption of this signaling axis in this RNF43-negative subgroup of PDAC cell lines through pharmacological inhibition of the O-acyltransferase porcupine, which is essential for WNT secretion, led to reduced cell proliferation, suggesting that their survival depends on a functional WNT–FZD signaling axis (Fig. 4*B*, *top panel*) (10, 39, 40, 41). Building upon these reports, we therefore hypothesized an alternative potential interference strategy: addressing the WNT–FZD signaling axis directly on the receptor level through a FZD-targeting molecule such as compound 11 should also reduce the viability of RNF43-negative and thus WNT-sensitive pancreatic cancer cells. Indeed, cell viability of RNF43-negative HPAF-II cells was strongly reduced upon treatment with compound 11 (Figs. 4*B* and S5). In contrast, PANC-1 cells, which do not carry an inactivating mutation in RNF43 and whose survival therefore does not depend on a functioning autocrine WNT signaling loop, were not significantly affected. These results provide further evidence that FZD-targeting small molecules indeed provide a therapeutic opportunity in cancer types stratified for WNT dependence that is caused for example, by RNF43 mutations but potentially also by overexpression of WNTs or FZDs.

## Discussion

Here, we report on the development of a small-molecule compound addressing FZDs in a paralog-nonselective manner. Derivatizing SAG1.3 has resulted in the discovery of DJ503701 (compound 11), which was not only able to efficiently suppress WNT–β-catenin signaling in HEK293 cells but also in hepatocyte-derived spheroids. Moreover, compound 11 reduced the survival of WNT signaling–dependent pancreatic cancer cells, thereby opening new therapeutic avenues.

The starting point of our study was the SMO agonist or FZD_6_ weak partial agonist SAG1.3, which was divided into three regions (R^1^–R^3^, Fig. 1*A*). SAG1.3 derivatives modified in one or more regions were synthesized and screened for binding to FZD_6_. It was evident from the screening results that 10 of the 12 compounds selected for further characterization were only modified in R^3^ compared with only two that were modified in other regions. Full concentration–response curves in competition binding experiments at Nluc-FZD_6_ revealed compound 11 as the one with the highest potency in our set, exhibiting IC_50_ values in the three-digit nanomolar to single-digit micromolar range, while not showing subtype selectivity within the FZD family (Fig. 2*C*). Being based on SAG1.3 and supported by binding experiments using a FZD_6_ construct lacking the extracellular region (ΔCRD-Nluc-FZD_6_, Fig. 2*D*), we propose that compound 11 binds to the transmembrane region rather than the orthosteric WNT binding site, the CRD.

A functional characterization using readouts for WNT–β-catenin signaling and the dynamic changes in the FZD_5_–DEP interface suggests that compound 11 reduces WNT-induced and FZD-mediated effects without possessing any intrinsic efficacy on FZDs (Fig. 3). Collectively, our binding and functional data suggest that compound 11 acts through the core of FZDs as an allosteric modulator. Nevertheless, we were not able to define the compound binding site experimentally, for example, through mutagenesis. Thus, we can so far only surmise that the mode of action of compound 11 is based on negative allostery.

In PHH-derived spheroids as an example for a more complex cell model, compound 11 suppressed WNT-induced target gene transcription close to basal levels (Fig. 4). Compound 11 was moreover capable of reducing the viability of a specific group of RNF43-mutated pancreatic cancer cells (*e.g.*, HPAF-II), whose survival is dependent on functional WNT signaling (10, 39). Thus, this can serve as a proof of concept of addressing FZDs in a therapeutically relevant context, substantially expanding the concept of targeting FZDs with CRD-binding biologics in the same experimental model (10).

Currently, several paradigms coexist, explaining the mechanisms underlying WNT signal initiation and pathway specification. One of them is the signalosome model, according to which the initiation of WNT signaling is achieved by WNTs serving as crosslinkers between FZDs and certain coreceptors, for example, LRP5/6 for WNT–β-catenin signaling, thereby specifying the signaling outcome (42, 43). While the signalosome model excludes intrinsic receptor dynamics (44, 45, 46), FZDs behave—in agreement with what is known for other GPCRs—as dynamic entities, sometimes referred to as molecular machines or gearboxes (26, 47, 48). The latter model includes an allosteric coupling between the CRD—the orthosteric WNT binding site—and the intracellular transducer coupling interface (25, 26, 49, 50). Without contradicting the necessity and relevance of coreceptors, the ability of small molecules to modulate WNT- and especially WNT surrogate–induced β-catenin signaling emphasizes the possibility that the CRD and the receptor core are allosterically interconnected and cooperate to initiate WNT signaling. A model that explains signal initiation solely by complexing a signalosome in the absence of FZD dynamics cannot explain the reduction of WNT–β-catenin signaling by a small-molecule compound binding to the receptor core. Thus, our findings underline the need to integrate the signalosome model with functional FZD dynamics to fully understand how WNT signaling is initiated and specified.

Previous efforts of targeting FZDs pharmacologically resulted in diverse biologics targeting the CRD and various small molecules addressing mainly the receptor core. WNT surrogates were developed based on the signalosome concept as WNT mimics by crosslinking FZDs and LRP5/6, eventually inducing WNT–β-catenin signaling (12, 13). While the development of WNT surrogates undoubtedly presents an exciting opportunity for applications in regenerative medicine, promoting, for example, alveolar regeneration after injury (9), their positive efficacy limits their use in the treatment of diseases presenting with overactive WNT signaling, for example, certain types of cancer. To fill this gap, other biomolecules, including peptides, antibodies, or antibody fragments, were investigated, which block WNT binding to the CRD of FZDs (10, 15, 51, 52, 53). Many of the developed antibodies showed promising effects on tumor growth and in *in vitro* studies. Most prominently, vantictumab, a monoclonal antibody binding to five of 10 FZD paralogs, even made it to phase Ib clinical trials until being dismissed because of bone toxicity (14, 54).

As an alternative strategy, there were a few reports over the last years on small organic compounds binding to the transmembrane core of FZDs even though this region of the receptors was deemed undruggable (17). Despite several reports on supposedly FZD-targeting small molecules capable of suppressing WNT signaling, the mechanisms of action of these compounds were poorly defined and mostly not FZD-dependent (19, 22, 23, 55). Despite the diversity of the assays that we present here to corroborate compound 11 action on FZDs, the lipophilicity of the substance and the potential of perturbing secondary effects are high, as is obvious by the effect on Fluc. Nevertheless, the effects on WNT surrogate–induced effects argue that compound 11 does not exert its effects by preventing WNT action on FZDs by interaction with the ligands directly, strengthening our conclusions that compound 11 acts through FZDs. Furthermore, the series of compounds developed during this work comprises substances structurally related to compound 11 with comparable solubility and lipophilicity, which, however, do not exert a strong inhibitory effect on FZD-mediated effects, arguing that lipophilic interference cannot explain the observed effects.

In the present study, we further extend the concept of the modulation of WNT signaling with small molecules by targeting receptor activation instead of directly interfering with WNT binding. We foresee that this strategy can indeed present a valid direction for future FZD-directed drug discovery. By definition, receptors only possess one orthosteric binding site while having many more allosteric binding pockets, which increases the number of potentially targetable sites (56). Moreover, it is a hallmark of allosteric modulators (without intrinsic efficacy) that they only affect receptor signaling in the presence of the respective orthosteric ligand, thus “fine-tuning” its response (57). With respect to eventually using drugs targeting the WNT–FZD signaling axis in a clinical setting, we envision that modulating or tuning the WNT response instead of interfering with WNT binding and thus completely blocking signaling, as done with CRD-targeting antibodies, might be advantageous in terms of the drug safety profile, especially when achieved in a paralog- and eventually pathway-selective manner.

The characterization of FZD-targeting small molecules does not only provide useful tool compounds but also deeper insight into FZD activation mechanisms and dynamics. The series of SAG1.3 derivatives presents a conceptual starting point for further refinement and discovery of compounds with higher affinity and potentially FZD paralog selectivity. Achieving paralog selectivity can be instrumental in finding a window of opportunity for human therapy by balancing therapeutic and undesired on-target side effects in FZD-targeting therapeutic approaches.

## Experimental procedures

### Cell culture and transfection

HEKs (HEK293A, female origin; Thermo Fisher Scientific), ΔFZD_1–10_ HEK293T cells (kindly provided by Benoit Vanhollebeke) (58), ΔSMO HEK293A cells (59) and the pancreatic cancer cell lines HPAF-II (American Tissue Culture Collection) and PANC-1 (American Tissue Culture Collection) were routinely cultured in complete Dulbecco’s modified Eagle’s medium (DMEM; Hyclone), supplemented with 1% penicillin–streptomycin (Gibco; catalog no.: 151-40122) and 10% fetal bovine serum (Gibco) in a humidified 5% CO_2_ incubator at 37 °C. All used cell culture plastics were purchased from Sarstedt or VWR, unless specified otherwise. Whenever indicated, cells were transiently transfected in suspension with 1 μg of total plasmid DNA per ml cell suspension using either Lipofectamine 2000 (Invitrogen; Lipofectamine [μl]:DNA [μg] = 2:1) or linear polyethyleneimine (PEI; Alfa Aesar, molecular weight 25,000, stock solution: 1 mg/ml; PEI [μl]:DNA [μg] = 5:1) as the transfection reagents. All transfected plasmid amounts indicated below refer to the amount of plasmid DNA used to transfect 1 ml of density-adjusted cell suspension.

The absence of mycoplasma contamination was routinely confirmed by PCR using 5′-GGC GAA TGG GTG AGT AAC ACG-3′ (forward) and 5′-CGG ATA ACG CTT GCG ACT ATG-3′ (reverse) primers, which were designed to detect 16S ribosomal RNA of mycoplasma in the media after at least 3 days of cell exposure.

### Plasmids and stable cell line generation

Plasmids encoding HiBiT-FZD_4_, HiBiT-FZD_5_, HiBiT-FZD_6_, HiBiT-FZD_7_, HiBiT-FZD_8_, Nluc-FZD_4_, and Nluc-FZD_6_ have been described previously (60, 61). Plasmids encoding Nluc-FZD_5_ and Nluc-FZD_7_ were generated by replacing the FZD_4_ sequence in the Nluc-FZD_4_ backbone with the respective nucleotide sequences for FZD_5_ and FZD_7_ *via* the BamHI and XbaI restriction sites in HiBiT-FZD_5_ and HiBiT-FZD_7_ (60). ΔCRD-Nluc-FZD_6_ was cloned by amplifying ΔCRD-FZD_6_ (starting from D156) using SNAP-FZD_6_ as a template (a kind gift from Madelon M. Maurice [University Medical Center Utrecht, Utrecht, The Netherlands]). The amplicon was then also cloned into the Nluc-FZD_4_ backbone using BamHI and XbaI restriction sites. To generate the FZD_5_–DEP–Clamp (thymidine kinase [TK]) plasmid, which was used to generate the stable FZD_5_–DEP–Clamp HEK293A cell line, the sequence for the human TK promoter (note: weak promoter sequence) was amplified from pRL-TK (Promega; E2241) and used to replace the cytomegalovirus promoter present in the original FZD_5_–DEP–Clamp plasmid (26) *via* Gibson assembly. HA-M_1_R-Nluc was generated by cloning the coding sequence of M_1_R (from cDNA.org) into the backbone of HA-FZD_4_-Nluc (47). The Super 8X TOPFlash plasmid was obtained from Addgene (catalog no.: 12456), and the Renilla luciferase (Rluc) control plasmid pRL-TK was from Promega. Venus mGsq was a kind gift from Nevin Lambert (Augusta University) (29). Salmon sperm DNA was from Thermo Fisher Scientific.

Primer sequences used to generate new plasmids are listed in Table S4. Sequences of all newly generated plasmids were verified by Sanger sequencing (Eurofins Genomics).

To generate the stable FZD_5_–DEP–Clamp cell line, HEK293A cells were seeded at a density of 500,000 cells/well in a 6-well plate. On the next day, the adherent cells were transfected with 2 μg of FZD_5_–DEP–Clamp (TK) plasmid using PEI as the transfection reagent. After 2 days of transfection, cells were detached and transferred to a 75 cm^2^ flask. Stably transfected cells were selected using complete medium (DMEM + 10% fetal calf serum + 1% penicillin–streptomycin) supplemented with 2000 μg/ml G-418 sulfate (Gibco; catalog no.: 10131027) for 3 weeks. After obtaining stable growth, cell culture of the stable cell line was continued with 500 μg/ml G-418.

### PHH cell culture

Cryopreserved adult PHHs were purchased from BioIVT and cultured as 3D spheroids as previously described (35). Briefly, PHHs were seeded in 96-well ultra-low attachment plates (Corning) at a density of 1500 cells/well in William’s E medium (Gibco) supplemented with insulin, transferrin, and selenium (Life Technologies), 100 nM dexamethasone (Sigma), and 10% fetal bovine serum (Cytiva). PHHs were treated from the start of the culture with vehicle control (dimethyl sulfoxide [DMSO]), recombinant WNT-3A, and 10 μM compound 11 for 48 h, as indicated. The supplier BioIVT collected informed consent from each donor or the subject’s legally authorized representative, and the documentation was reviewed and approved by the appropriate regulatory authorities in accordance with US Department of Health and Human Services regulations for the protection of human subjects (45 CFR §46.116 and §46.117) and Good Clinical Practice (ICH E6). The demographics and medical history of the donor used are reported in Table S5.

### Ligands

Synthesis procedures for compounds selected for further characterization after the first screen (9, 10, 11, 14, 15, 17, 18, 19, 21, 23, 24, and 52), including the respective analytical characterization, can be found in the supporting information. Stock solutions of all newly synthesized small molecules were prepared in pure DMSO at a concentration of 10 mM. SAG1.3 (IUPAC: 3-chloro-*N*-[trans-4-(methylamino)cyclohexyl]-*N*-[[3-(4-pyridinyl)phenyl]methyl]-benzo[b]thiophene-2-carboxamide dihydrochloride) was purchased from Sigma–Aldrich (catalog no.: SML1314) and dissolved at a concentration of 10 mM in Millipore water. BODIPY–cyclopamine (BioVision; catalog no.: 2160) was dissolved at 1 mM in DMSO and stored in aliquots. The porcupine inhibitor C59 (IUPAC: 2-[4-(2-methylpyridin-4-yl)phenyl]-*N*-[4-(pyridin-3-yl)phenyl]acetamide, Abcam, catalog no.: ab142216) was dissolved at 10 mM in DMSO and used wherever indicated to reduce endogenous WNT secretion. All stock solutions of small-molecule compounds were stored at −20 °C.

Lyophilized recombinant WNT-3A (R&D Systems; 5036-WN-010) and high-purity WNT-3A (R&D Systems; 5036-WNP-010) were resuspended in 0.1% bovine serum albumin (Sigma–Aldrich)/Dulbecco’s PBS (Hyclone) at a concentration of 100 μg/ml or 200 μg/ml, respectively. WNT-surrogate-Fc fusion protein (WNT surrogate, U-Protein Express B.V., catalog no.: N001) was diluted to a concentration of 500 nM with 0.1% bovine serum albumin/Dulbecco’s PBS. WNT-3A and WNT surrogate stock solutions were stored at 4 to 8 °C for a maximum of 6 weeks.

### BRET-based binding assays

One day prior the experiment, ΔFZD_1–10_ HEK293T (400,000 cells/ml) or ΔSMO HEK293A cells (300,000 cells/ml) were transiently transfected in suspension with 10 ng of Nluc-FZD_x_ and 990 ng of empty pcDNA3.1 (to adjust the DNA amount) per ml cell suspension and seeded (ΔFZD_1–10_ HEK293T cells: 40,000 cells/well, ΔSMO HEK293A cells: 30,000 cells/well) into poly-d-lysine (PDL)–coated, white opaque 96-well plates (Nunc; Thermo Fisher Scientific). After 24 h, cells were washed once with Hanks' balanced salt solution (HBSS), followed by the addition of 80 μl of HBSS. Next, 10 μl of the competitive ligand, that is, the tested SAG1.3 derivative (either in different concentrations for the concentration–response curves or at a final concentration of 10 μM for the first screens) and 10 μl of the fluorescent tracer BODIPY–cyclopamine (final concentration: 300 nM for the first screens, 200 nM for the concentration–response experiments) were added. All serial dilutions were prepared in HBSS. The cells were incubated for 90 min at 37 °C without additional CO_2_, after which 10 μl of luciferase substrate diluted in HBSS (furimazine; Promega, N1572, final dilution: 1:1000 for first screening) and coelenterazine h (Biosynth; final concentration: 5 μM for concentration–response experiments) were added to the cells. Following a 10 min incubation period in the dark, the measurement was started on a BMG Labtech ClarioStar or TECAN Spark multimode microplate reader, prewarmed to 37 °C. Nluc bioluminescence was detected between 460 and 490 nm (ClarioStar) or 460 and 500 nm (TECAN Spark). The emission of the fluorescent tracer was detected between 520 and 550 nm (ClarioStar) or between 520 and 560 nm (TECAN Spark).

### TOPFlash reporter gene assay

ΔFZD_1–10_ HEK293T cells were transiently transfected in suspension (450,000 cells/ml) with a mix of 200 ng of HiBiT-FZD_x_ (paralog indicated in figures), 250 ng of Super 8X TOPFlash reporter (TCF/LEF activity–dependent Fluc expression), 75 ng of pRL-TK (constitutive Rluc expression as a transfection control), and 475 ng of empty pcDNA3.1 (to adjust the DNA amount) per ml cell suspension (450,000 cells/ml). The transfected cell suspension was then seeded (45,000 cells/well) into a PDL-coated, white opaque 96-well plate. One day after transfection, cells were washed once with HBSS, and 80 μl of serum-free DMEM containing 10 nM of the porcupine inhibitor C59 were added. Next, 10 μl of compound 11 (concentrations as indicated) or DMSO were added, followed by the addition of 10 μl of recombinant WNT-3A (final concentration: 300 ng/ml), WNT surrogate (final concentration: 250 pM), or their respective vehicle controls. All ligands/vehicle controls were diluted in serum-free DMEM containing 10 nM C59. One day after ligand stimulation, cells were washed once with HBSS, and the lysis was started using 1× Passive Lysis Buffer (Promega; catalog no.: E1910, 20 μl/well), after which the plate was shaken for 20 min at room temperature. Fluc and Rluc bioluminescence were then assessed using the Dual-Luciferase Assay System (Promega; catalog no.: E1910, 20 μl of both LARII [Luciferase Assay II reagent] and 1× Stop-and-Glo Reagent). Measurements were performed using a TECAN Spark multimode microplate reader. Fluc bioluminescence (proportional to the activation of β-catenin-dependent gene transcription) was detected between 550 and 620 nm (integration time: 2000 ms), whereas Rluc bioluminescence (constitutive transfection control measure) was measured between 445 and 530 nm (integration time: 2000 ms).

### NanoBiT cell surface expression assay

HEK293A cells were transiently transfected in suspension (350,000 cells/ml) with 100 ng of HiBiT-FZD_5_ or HiBiT-FZD_6_ and 900 ng of empty pcDNA3.1 (to adjust the DNA amount). The transfected cells were seeded at a density of 35,000 cells per well into a PDL-coated white opaque 96-well plate. After 1 day of transfection, cells were washed once with HBSS, and 90 μl of serum-free DMEM containing 10 nM of porcupine inhibitor C59 was added to each well. Subsequently, 10 μl of either compound 11 or 26 (DD436001) was added at a final concentration of 10 μM. DMSO was added separately as a control. One day after compound or DMSO addition, cells were washed once with HBSS, and 90 μl of HBSS was added to the wells. To measure cell surface expression, a 1:20 dilution of furimazine live cell substrate and 1:20 LgBiT mix (final dilution = 1:200) were prepared in HBSS and 10 μl was added. The plate was incubated for 30 min to allow for HiBiT–LgBiT complementation, and then luminescence was measured on a TECAN Spark multimode microplate reader between 460 and 500 nm (integration time: 200 ms).

### Venus-mGsq recruitment assay

HEK293A cells were transiently transfected in suspension (450,000 cells/ml) with 50 ng of M_1_R-Nluc, 250 ng of Venus-mGsq, and 700 ng of salmon sperm DNA (to adjust the DNA amount). The transfected cells were seeded at a density of 40,000 cells per well into a white opaque 96-well plate. After 1 day of transfection, cells were washed once with HBSS, and 90 μl of luciferase substrate diluted in HBSS (furimazine [Promega; N1572], final dilution: 1:1000) was added to each well. Following a 10 min incubation period in the dark, the measurement was started on a TECAN Spark multimode microplate reader, prewarmed to 37 °C. After the three baseline measurements, the ligands diluted in HBSS were added (10 μl) and the measurement continued for a further 15 min. Nluc bioluminescence was detected between 460 and 500 nm. The emission of Venus was detected between 520 and 560 nm.

### Gene expression analysis in primary hepatocytes

RNA from 24 hepatocyte spheroids per condition was isolated using Qiazol lysis reagent (QIAGEN). For expression profiling of target genes by quantitative PCR, complementary DNA synthesis was carried out using SuperScript III reverse transcriptase (Invitrogen), and expression was evaluated using Taqman probes (Table S6) according to the supplier’s instructions in a 7500 Fast Real-Time PCR system (Applied Biosystems). Gene expression was quantified using the ΔΔ*Ct* method (normalized to the average of the vehicle control from all three experiments).

### Cell viability

HPAF-II cells and PANC-1 cells were seeded in DMEM at a density of 1000 cells per well into a black opaque 96-well plate (Greiner BioOne). After 1 day, the medium was exchanged with complete DMEM containing either compound 11, the porcupine inhibitor C59, or DMSO (vehicle control). After 3 days of incubation (37 °C, 5% CO_2_), the medium was exchanged once again with DMEM containing the same concentration of ligands or DMSO (vehicle control). Three days after (equal to 7 days after seeding), the medium was removed and 90 μl of fresh DMEM (without any ligands) were added. Last, 10 μl of AlamarBlue HS reagent (Thermo Fisher Scientific; catalog no.: A50100) were added to each well, and the cells were incubated for 4 h at 37 °C inside the incubator (5% CO_2_). After the incubation step, fluorescence was read using a TECAN Spark multimode microplate reader (excitation: 535 ± 25 nm, emission: 595 ± 35 nm).

### Receptor modeling

In the compound design focusing on R^3^ modifications, we utilized FZD_6_ models as previously described (18). The model, where the docked SAG1.3 pose was in the best agreement with the SAG1.5 pose in the SMO-SAG1.5 crystal structure (Protein Data Bank ID: 4QIN) was selected for the docking studies (62). Docking was performed with AutoDock Vina 1.1.2, and SAG1.3 defined the location of the utilized docking grid. Ten poses per compound were written out, and they underwent visual analysis by which the most promising designs were selected for synthesis.

### Data and statistical analysis

All raw data from plate reader experiments were obtained as Microsoft Excel spreadsheets. The subsequent data analysis and visualization were performed in Microsoft Excel and GraphPad Prism 9.0 (GraphPad Software, Inc).

BRET was defined as the ratio of acceptor emission (BODIPY–cyclopamine/mVenus-tagged protein) over the donor emission (Nluc-tagged protein).

For each binding experiment, the measurement was repeated five times and averaged for the analysis. ΔBRET values were obtained by subtracting the BRET ratio obtained in wells containing BODIPY–cyclopamine but no competitive ligand (100% value). The BRET values for full displacement were defined using “donor-only” control wells, which contained Nluc-FZD_x_-transfected cells but no BODIPY–cyclopamine.

For the full concentration–response BRET binding curves in Figure 2, BRET values were normalized (separately for each experiment) to control wells containing BODIPY–cyclopamine but no competitive ligand (100% value) and “donor-only” wells (0% value, described previously). The normalized data were analyzed using three-parameter or four-parameter nonlinear regression, where the model was chosen for each independent experiment separately after running extra-sum-of-squares *F* tests (*p* < 0.05), resulting in an IC_50_ value. Obtained IC_50_ values were transformed to pIC_50_ values, which were subsequently averaged between experiments. Displacement curves showing the non-normalized data (ΔBRET values generated as described previously) can be found in Fig. S2.

For reporter gene assays, the TOPFlash ratio was defined as the ratio of β-catenin-dependent gene transcription (Fluc) over a constitutively expressed transfection control (Rluc). For all experiments, the calculated TOPFlash ratios were normalized to the respective vehicle conditions. For the concentration–response curve of compound 11 (Fig. 2*B*), the vehicle-corrected TOPFlash ratios were normalized (100% value) to the wells containing WNT-3A but no compound 11.

For kinetic experiments with the FZD_5_–DEP–Clamp sensor, BRET values were first baseline-corrected for every well separately by subtracting the average of the first three reads prior ligand addition. Afterward, the average values for vehicle-containing wells were subtracted for every timepoint to get access to actual ligand-induced changes in BRET ratio (ΔBRET values).

For the WNT target gene analysis in PHH-derived spheroids, ΔΔ*Ct* values were calculated by normalization to the averaged vehicle control from three independent experiments.

For each cell viability experiment, the fluorescence measured in ligand-treated wells was normalized to the average fluorescence from wells treated with vehicle control (DMSO).

Statistical significance was assessed using either one-way ANOVA or two-way ANOVA. In all instances, *p* < 0.05 was considered significant. Specific details are given in the respective figure legends.

## Data and materials availability

All relevant data generated and analyzed during this study are included in this article and its supporting information. Should any raw data files be needed, they are available from the corresponding author upon reasonable request (Gunnar Schulte—gunnar.schulte@ki.se). Expression vectors used and created for this work can be obtained from the corresponding author.

## Supporting information

This article contains supporting information (23, 34, 63, 64).

## Conflict of interest

V.M.L. is CEO and a shareholder of HepaPredict AB, as well as a cofounder and shareholder of Shanghai Hepo Biotechnology Ltd. All other authors declare that they have no conflicts of interest with the contents of this article.
