# Supplementary material for: SAG1.3-derived Frizzled-targeting small-molecule compounds
Source: J Biol Chem. 2025 Sep 22;301(11):110751. doi: 10.1016/j.jbc.2025.110751 (PMC12605013; doi:10.1016/j.jbc.2025.110751)
Supplement: Supporting information [file mmc1.docx]

**Supporting Information**

**SAG1.3-derived Frizzled-targeting small molecule compounds**

**Authors:** Lukas Grätz^1,#^, Ainoleena Turku^1,2,#^, Pawel Kozielewicz^1^, Carl-Fredrik Bowin^1,3^, Magdalena M. Scharf^1^, Jan H. Voss^1^, Julia Kinsolving^1^, Rawan Shekhani^1^, Nuria Oliva-Vilarnau^4^, Tobias Koolmeister^5^, Marlies Körber^6^, Volker M. Lauschke^4,7,8^, Stefan Löber^6,9^, Peter Gmeiner^6,9^, Gunnar Schulte^1,*^

**Affiliations:**

^1^Karolinska Institutet, Dept. Physiology & Pharmacology, Sec. Receptor Biology & Signaling, Biomedicum, Stockholm, Sweden

^2^current address: Orion Pharma R&D, Espoo, Finland

^3^current address: University of Copenhagen, Department of Neuroscience, Copenhagen, Denmark

^4^Karolinska Institutet, Dept. Physiology & Pharmacology, Sec. Personalized Medicine and Drug Development, Biomedicum, Stockholm, Sweden

^5^Chemical Biology Consortium Sweden, Science for Life Laboratory, Department of Medical Biochemistry and Biophysics, Karolinska Institutet, Stockholm, Sweden

^6^Department of Chemistry and Pharmacy, Friedrich-Alexander-Universität Erlangen-Nürnberg, Erlangen, Germany

^7^Dr. Margarete Fischer-Bosch Institute of Clinical Pharmacology, Stuttgart, Germany

^8^University of Tübingen, Tübingen, Germany

^9^FAUNeW – Research Center New Bioactive Compounds, Friedrich-Alexander-Universität Erlangen-Nürnberg, Erlangen, Germany

^#^Authors contributed equally

*To whom correspondence should be addressed: gunnar.schulte@ki.se

**Figure S1**

**
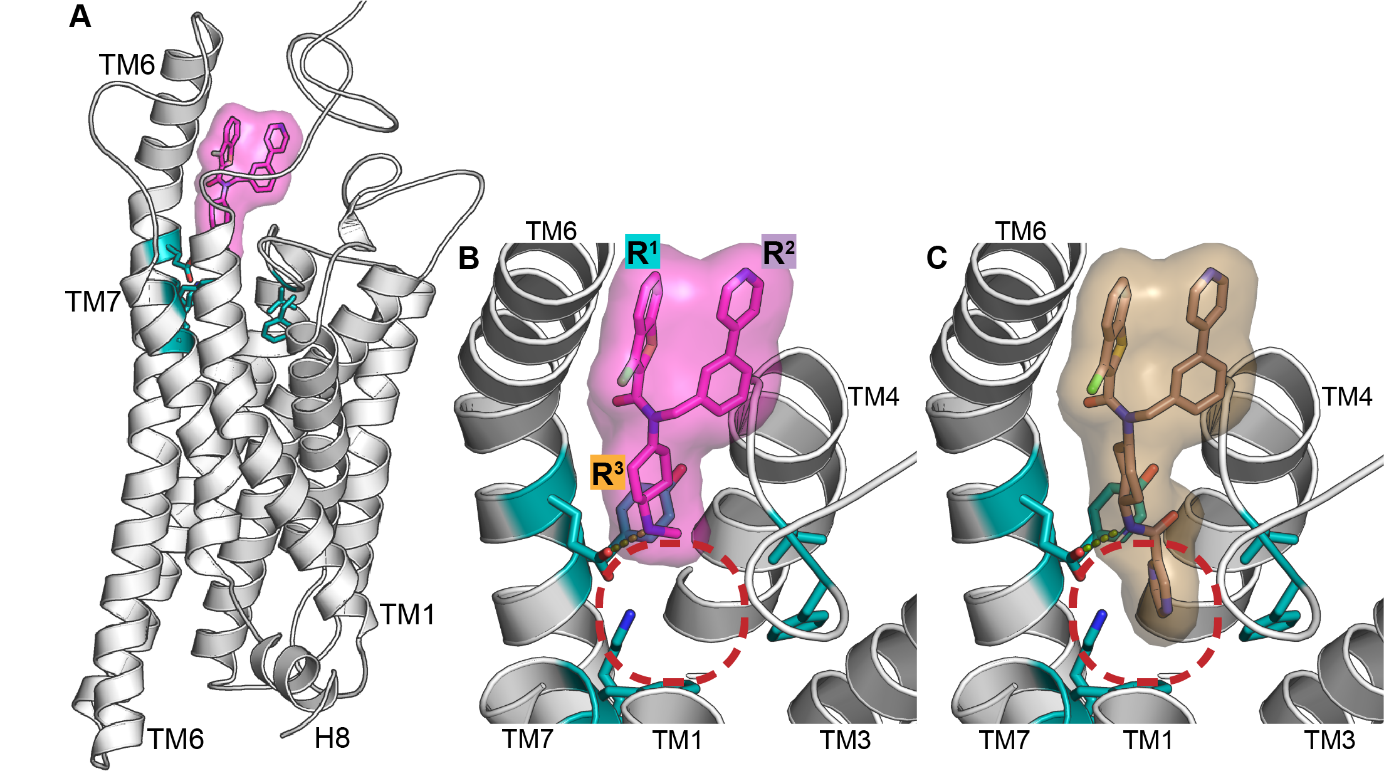
**

# Figure S1. Molecule pose used for the initial compound design. (A) Side-view of the FZD_6_ receptor model. The predicted binding site of parent compound SAG1.3 (pink sticks and surface) is at the extracellular side of the receptor, close to the extended TM6. (B) Close-up of the docking pose of parent compound SAG1.3 (pink sticks and surface) within the predicted binding site of the FZD_6_ model. The three regions that were modified during this study (R^1^-R^3^) are indicated. The red circle highlights the region that was predicted to accommodate modifications of R^3^ in an aromatic sub-pocket. (C) Docking pose of compound 9 (MRG32; sand). The modification in R^3^ compared to SAG1.3 is placed in the sub-pocket highlighted by the red circle. Numbering of relevant TMs is added in all sub-panels for better orientation.

**Figure S2**


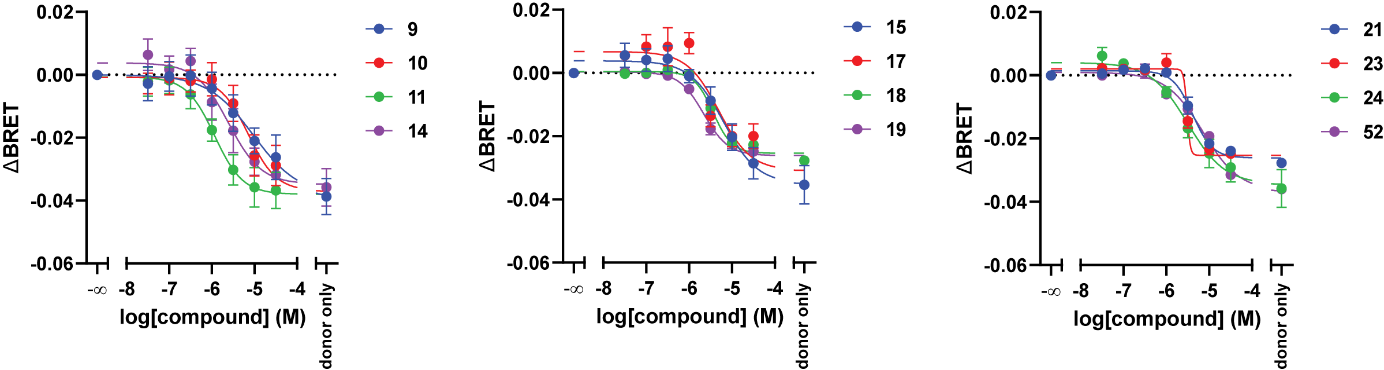


# Figure S2. ΔBRET values from competition binding experiments at Nluc-FZD_6_. ΔBRET values were calculated by subtracting the values from wells containing BODIPY-cyclopamine but no competitive ligand. Values on the right side of the broken axis represent “donor only” wells indicative of full tracer displacement. Shown data represent mean values ± SEM from three independent experiments performed in triplicate. Normalized data is shown in Figure 2a.

**Figure S3**

**
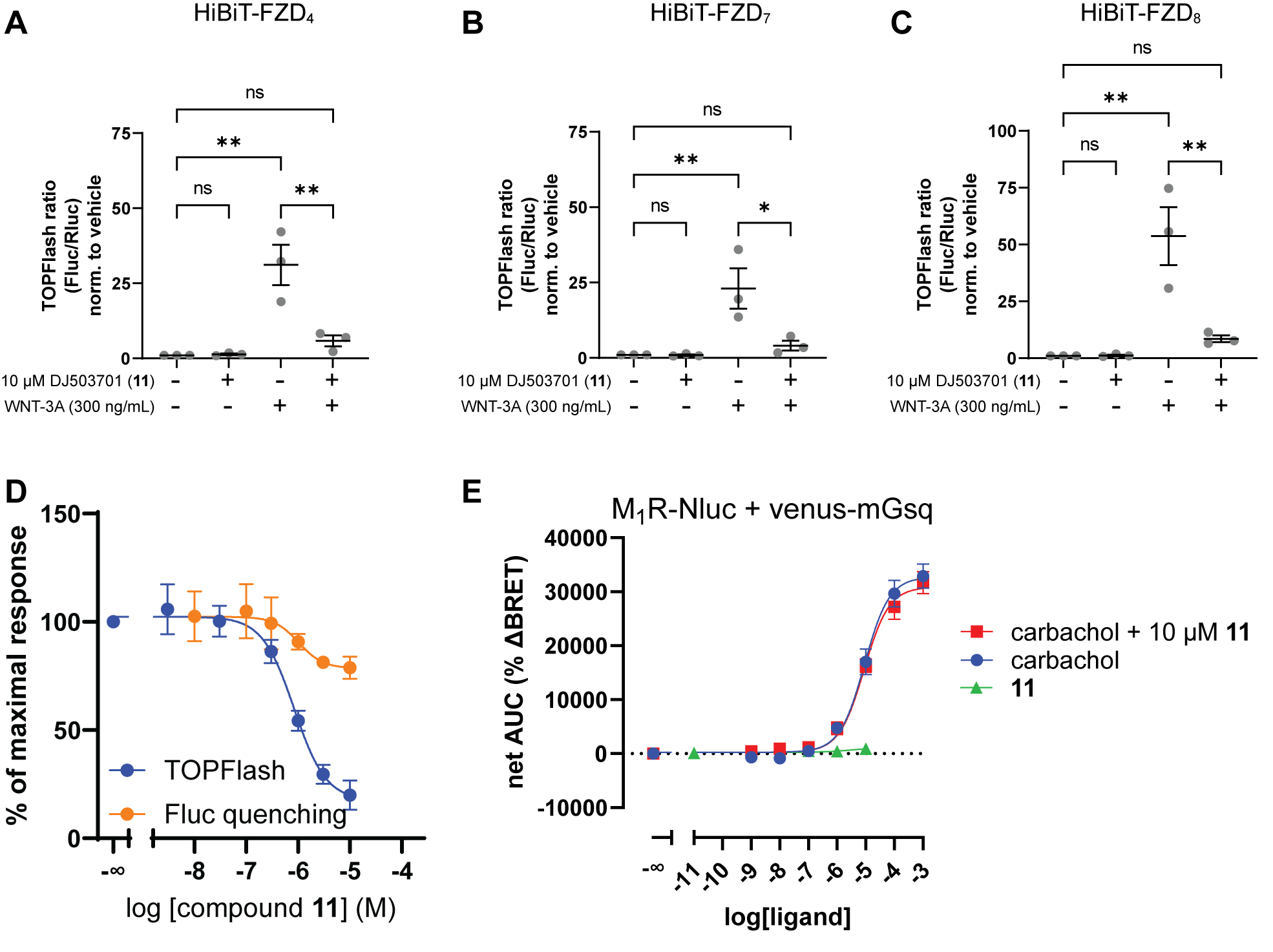
**

# Figure S3. Test for paralog selectivity of compound 11 in TOPFlash reporter gene assays. TOPFlash reporter gene responses upon addition of vehicle or 300 ng/mL WNT-3A in the presence or absence of 10 µM DJ503701 (compound 11). Experiments were performed in ΔFZD_1-10_ HEK293T cells transiently transfected with HiBiT-FZD_4_ (A), HiBiT-FZD_7_ (B) or HiBiT-FZD_8_ (C). Data represent mean values ± SEM from three independent experiments performed in triplicate. Statistical significance was assessed using one-way ANOVA followed by Tukey’s post-hoc test. ns: not significant; *: p < 0.05; **: p < 0.01. (D) The curves compare the effect of compound 11 on the WNT-3A-induced TOPFlash response (blue) and the pure Fluc quenching using transfection of recombinant Fluc alone. Therefore, the transient expression of Fluc driven by a CMV promotor, instead of a WNT-responsive TCF/LEF responsive element, was used as counter assay to control for compound interference with the assay readout^1^. Experiments were performed in HEK293 cells. The data (n=3) are normalized to the maximal response in each individual data set. Note that the TOPFlash data was copied from main Figure 3B. (E) Compound 11 was tested for interference with muscarinic M_1_ receptors in HEK293 cells in a BRET-based mini Gsq (Venus-mGsq) protein recruitment assay. Cells were stimulated with increasing concentrations of compound 11 in agonist mode or with 10 µM compound 11 +/- increasing concentrations of the M_1_R agonist carbachol. Data (mean ± SEM) from 5 independent experiments are summarized.

**Figure S4**

**
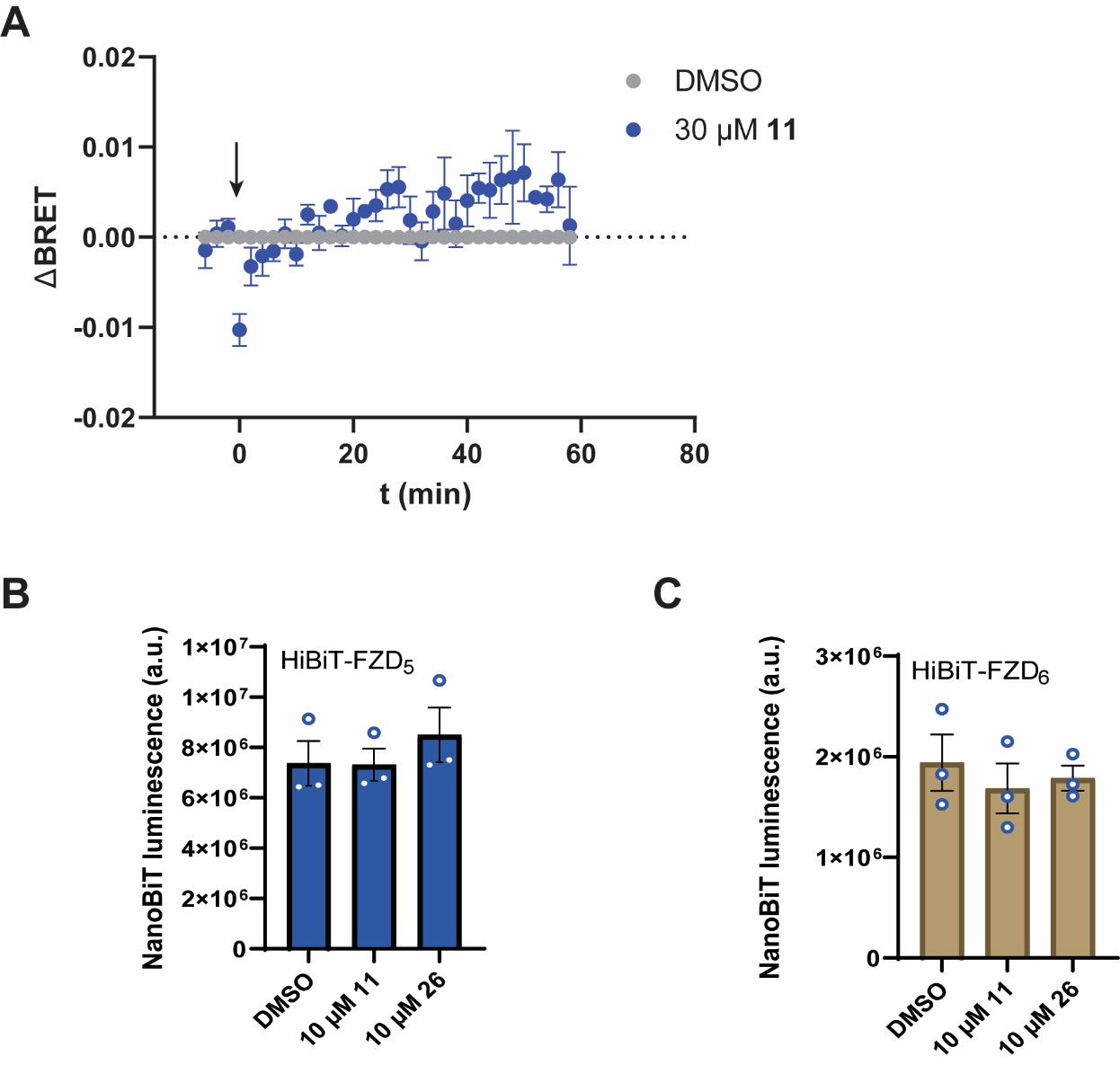
**

# Figure S4. BRET time-course of compound 11 at the FZD_5_-DEP-Clamp in agonist mode and HiBiT-FZD_5/6_ surface expression upon compound exposure. (A) Shown is the BRET time-course recorded upon addition of 30 µM compound 11 or vehicle (DMSO). Experiments were performed in HEK293A cells stably expressing the FZD_5_-DEP-Clamp sensor. Data represent mean values ± SEM from three independent experiments each performed in triplicate. The timepoint of ligand addition is indicated by a black arrow. (B, C) Impact of compound 11 on FZD cell surface expression. The cell surface pool of transiently-transfected HiBiT-FZD_5_ (B) and HiBiT-FZD_6_ (C) was assessed by addition of recombinant LgBiT in combination with detection of NanoBiT luminescence. The transfected HEK293 cells were treated with DMSO, compound 11 (competing with BODIPY-cyclopamine) or compound 26 (structurally related to compound 11 but not competing with BODIPY-cyclopamine). Compound exposure was for one day matching the time course of the TOPFlash assays presented in Figure 3. Data represent mean values ± SEM from three independent experiments

**Figure S5**


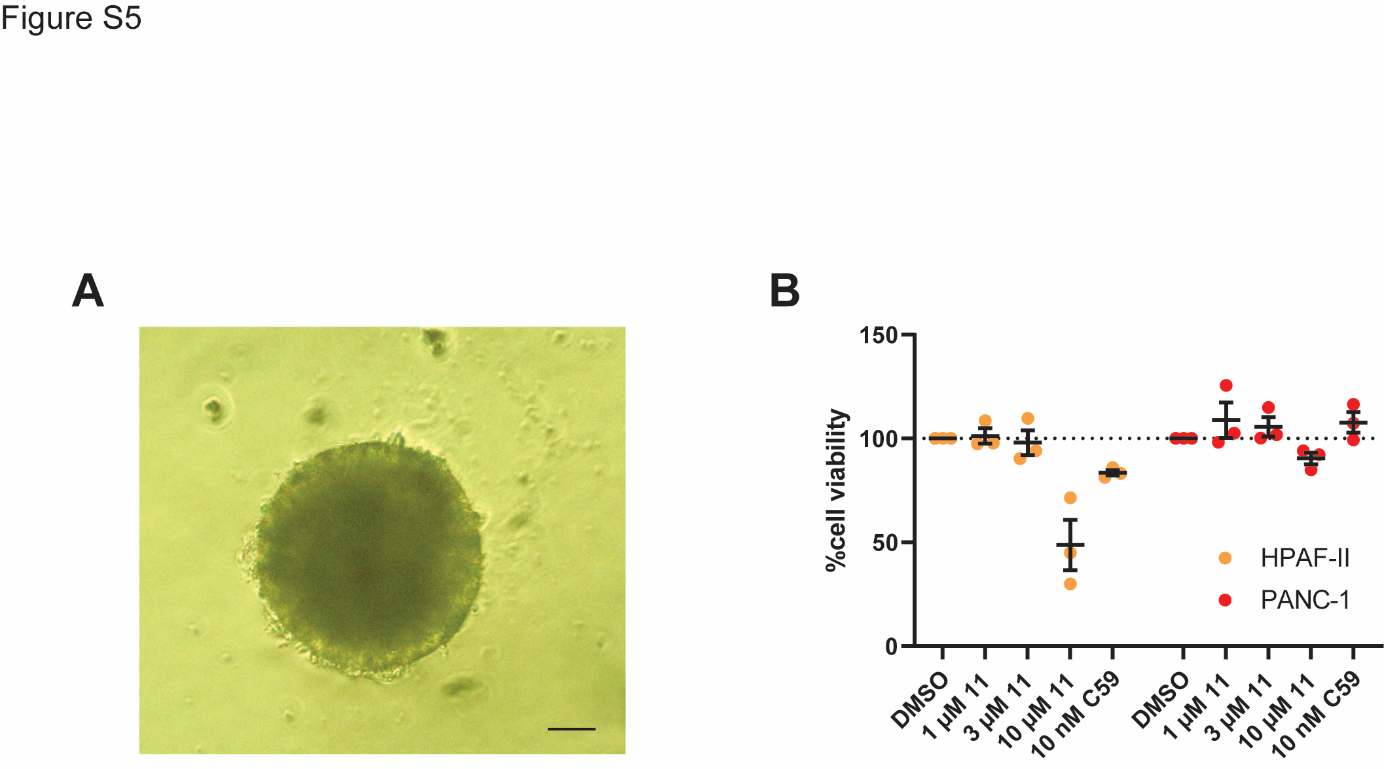


# Figure S5. Application of compound 11 in PHH and pancreatic cancer cell models.. (A) A bright field microscopy image of a PHH spheroid, representative of those used for experiments in Figure 4a. The PHH spheroid model was previously established and validated ^2,3^. Size bar = 100 µm. (B) Cell viability of RNF43-negative HPAFII and RNF43-wild type PANC-1 cells was assessed after one-week treatment with the indicated concentrations of compound 11 or the porcupine inhibitor C59. Data represent normalized mean values ± SEM from three independent experiments performed in triplicate. Values for DMSO and 10 µM 11 are copied from main Figure 4b for illustration purposes.

# Table S1: List of all synthesized compounds and ΔBRET values from BRET-based binding screen.

ΔBRET values were obtained from BRET-based competition binding experiments using 10 µM of SAG1.3/compound **1**-**59** and BODIPY-cyclopamine (c = 300 nM). Experiments were performed in ΔSMO HEK293A or ΔFZD_1-10_ HEK293T cells transiently transfected with Nluc-FZD_6_. Shown are mean values and SDs from N independent experiments (N = 2-8) performed in duplicate. Compounds in cells labeled with light green shading were measured on a TECAN Spark microplate reader (complete tracer displacement: net BRET = -0.03823), while all other compounds were measured on a BMG Labtech ClarioStar microplate reader (complete tracer displacement: net BRET = -0.02736). Compounds surpassing the predefined cut-off value of 70% tracer displacement are written in **bold red text**. Changes of R^1^, R^2^ and R^3^ compared to the parent compound SAG1.3 are highlighted by grey shading.

| **Cmpd No.** | **Cmpd name** | **R^1^** | **R^2^** | **R^3^** | **Displacement @ 10 µM compound (Nluc-FZD_6_)** | | | **Displacement %** |
| --- | --- | --- | --- | --- | --- | --- | --- | --- |
|  |  |  |  |  | **ΔBRET** | **SD** | **N** |  |
|  | SAG1.3 |  |  |  | -0.0099 | 0.00512 | 8 | 36.1% |
|  |  |  |  |  | -0.0148 | 0.01483 | 3 | 38.7% |
| **1** | DD435401 |  |  |  | -0.0160 | 0.00558 | 3 | 58.6% |
| **2** | DD434001 |  |  |  | 0.0096 | 0.005645 | 6 | -34.9% |
| **3** | DD433701 |  |  |  | 0.0034 | 0.003067 | 6 | -12.4% |
| **4** | DD433801 |  |  |  | -0.0054 | 0.002358 | 6 | 19.7% |
| **5** | DD433901 |  |  |  | -0.0038 | 0.003055 | 6 | 13.8% |
| **6** | DD434201 |  |  |  | -0.0082 | 0.004765 | 6 | 29.9% |
| **7** | DD434401 |  |  |  | -0.0046 | 0.005549 | 6 | 16.7% |
| **8** | MRG31 |  |  |  | -0.0176 | 0.003829 | 6 | 64.5% |
| **9** | **MRG32** |  |  |  | **-0.0197** | **0.003702** | **5** | **72.1%** |
| **10** | **MRG33** |  |  |  | **-0.0207** | **0.0009263** | **2** | **75.5%** |
| **11** | **DJ503701** |  |  |  | **-0.0228** | **0.004593** | **3** | **83.2%** |
| **12** | DJ503801 |  |  |  | -0.0159 | 0.008136 | 3 | 58.1% |
| **13** | DJ503901 |  |  |  | -0.0119 | 0.006255 | 3 | 43.4% |
| **14** | **DJ508501** |  |  |  | **-0.0290** | **0.006142** | **3** | **75.9%** |
| **15** | **DJ508401** |  |  |  | **-0.0268** | **0.005491** | **3** | **70.1%** |
| **16** | DJ507701 |  |  |  | -0.0196 | 0.005407 | 3 | 51.3 |
| **17** | **DJ508701** |  |  |  | **-0.0281** | **0.003942** | **3** | **73.6%** |
| **18** | **DQ090601** |  |  |  | **-0.0310** | **0.00915** | **3** | **81.1%** |
| **19** | **DQ090801** |  |  |  | **-0.0335** | **0.01164** | **3** | **87.6%** |
| **20** | DQ090501 |  |  |  | -0.0207 | 0.002763 | 3 | 54.1% |
| **21** | **DQ090001** |  |  |  | **-0.0299** | **0.005978** | **3** | **78.2%** |
| **22** | DJ509801 |  |  |  | -0.0262 | 0.00437 | 3 | 68.6% |
| **23** | **DQ090901** |  |  |  | **-0.0328** | **0.006846** | **3** | **85.8%** |
| **24** | **DJ509001** |  | | | **-0.0298** | **0.01285** | **3** | **78.1%** |
| **25** | DJ501501 |  |  |  | 0.0109 | 0.006382 | 3 | -36.8% |
| **26** | DD436001 |  |  |  | -0.0001 | 0.002218 | 3 | 0.4% |
| **27** | DD435701 |  |  |  | -0.0008 | 0.002357 | 3 | 2.8% |
| **28** | DD435601 |  |  |  | -0.0039 | 0.003461 | 3 | 14.2% |
| **29** | DD435801 |  |  |  | -0.0079 | 0.002522 | 3 | 28.8% |
| **30** | DD435901 |  |  |  | -0.0051 | 0.001721 | 3 | 18.5% |
| **31** | DD434901 |  |  |  | 0.0011 | 0.001351 | 3 | -3.9% |
| **32** | DD435201 |  |  |  | -0.0021 | 0.004303 | 3 | 7.8% |
| **33** | DD435001 |  |  |  | 0.0035 | 0.003355 | 3 | -12.9% |
| **34** | DD435101 |  |  |  | -0.0005 | 0.003616 | 3 | 1.8% |
| **35** | DD438401 |  |  |  | -0.0005 | 0.00237 | 3 | 2.0% |
| **36** | DD438701 |  |  |  | -0.0178 | 0.004365 | 3 | 65.0% |
| **37** | DD438901 |  |  |  | -0.0118 | 0.006054 | 3 | 43.0% |
| **38** | DD436501 |  |  |  | -0.0032 | 0.003462 | 3 | 11.8% |
| **39** | DD436601 |  |  |  | -0.0005 | 0.003095 | 3 | 1.9% |
| **40** | DD436701 |  |  |  | -0.0017 | 0.003905 | 3 | 6.4% |
| **41** | DD436801 |  |  |  | -0.0175 | -0.001892 | 3 | 63.9% |
| **42** | DJ502601 |  |  |  | -0.0082 | 0.006238 | 3 | 30.1% |
| **43** | DD436901 |  |  |  | -0.0122 | 0.004431 | 3 | 44.7% |
| **44** | DD439301 |  |  |  | -0.0096 | 0.009168 | 3 | 35.1% |
| **45** | DD439401 |  |  |  | -0.0118 | 0.008718 | 3 | 43.0% |
| **46** | DD439501 |  |  |  | -0.0102 | 0.003167 | 3 | 37.2% |
| **47** | DD439901 |  |  |  | -0.00967 | 0.003167 | 3 | 35.3% |
| **48** | DJ500001 |  |  |  | -0.0116 | 0.002952 | 3 | 42.5% |
| **49** | DJ500101 |  |  |  | -0.0090 | 0.003404 | 3 | 32.7% |
| **50** | DJ500301 |  |  |  | -0.0127 | 0.008314 | 3 | 46.6% |
| **51** | DJ500201 |  |  |  | -0.0128 | 0.002707 | 3 | 46.7% |
| **52** | **MRG26** |  |  |  | **-0.0194** | **0.005906** | **7** | **70.9%** |
| **53** | DJ500701 |  |  |  | -0.0087 | 0.004885 | 3 | 31.7% |
| **54** | DJ500801 |  |  |  | -0.0067 | 0.008789 | 3 | 24.6% |
| **55** | DJ502901 |  | | | -0.0113 | 0.006216 | 3 | 41.2% |
| **56** | DJ503001 |  | | | -0.0046 | 0.008961 | 3 | 16.8% |
| **57** | DJ503101 |  | | | -0.0108 | 0.006009 | 3 | 39.4% |
| **58** | DJ503201 |  | | | -0.0103 | 0.003722 | 3 | 37.8% |
| **59** | DJ503301 |  | | | -0.0087 | 0.005511 | 3 | 31.8% |

# Table S2: pIC_50_ values from competition binding experiments at Nluc-FZD_6_.

| **Compound** | | **pIC_50_ (Nluc-FZD_6_) mean ± SEM** | **N** |
| --- | --- | --- | --- |
| **No.** |  |  |  |
| **9** | MRG32 | 5.41 ± 0.04 | 3 |
| **10** | MRG33 | 5.29 ± 0.02 | 3 |
| **11** | DJ503701 | 5.99 ± 0.12 | 3 |
| **14** | DJ508501 | 5.57 ± 0.17 | 3 |
| **15** | DJ508401 | 5.13 ± 0.16 | 3 |
| **17** | DJ508701 | 5.49 ± 0.04 | 3 |
| **18** | DQ090601 | 5.50 ± 0.04 | 3 |
| **19** | DQ090801 | 5.71 ± 0.04 | 3 |
| **21** | DQ090001 | 5.44 ± 0.05 | 3 |
| **23** | DQ090901 | 5.54 ± 0.03 | 3 |
| **24** | DJ509001 | 5.59 ± 0.09 | 3 |
| **52** | MRG26 | 5.06 ± 0.04 | 3 |

pIC_50_ values were determined in BRET-based competition binding experiments with the indicated compounds and the tracer molecule BODIPY-cyclopamine (c = 200 nM). Experiments were performed in ΔFZD_1-10_ HEK293T cells transiently transfected with Nluc-FZD_6_. Shown are mean values ± SEM from N independent experiments performed in triplicate.

**Table S3: pIC_50_ values for compound 11 from competition binding experiments at different Nluc-FZD_x_.**

|  | **pIC_50_ (11) mean ± SEM** | **N** |
| --- | --- | --- |
| Nluc-FZD_4_ | 6.02 ± 0.09 | 3 |
| Nluc-FZD_5_ | 6.12 ± 0.02 | 3 |
| ΔCRD-Nluc-FZD_6_ | 6.07 ± 0.05 | 3 |
| Nluc-FZD_7_ | 6.05 ± 0.05 | 3 |

pIC_50_ values were determined in competition binding experiments with **11** and the tracer molecule BODIPY-cyclopamine (c = 200 nM). Experiments were performed in ΔFZD_1-10_ HEK293T cells transiently transfected with the indicated Nluc-FZD_x_ construct. Shown are mean values ± SEM from N independent experiments performed in triplicate.

# Table S4: Primers used in the study.

| **primer name** | **primer sequence (5’ – 3’)** |
| --- | --- |
| ΔCRD-Nluc-FZD_6__FW | GACGGATCCGACATTGGATTTTGGTGTCCA |
| Nluc-FZD_6__RV | AACGGGCCCTCTAGATTAAGTATCTGAATGACA |
| lin pcDNA3.1 promoter_FW | TCTGGCTAACTAGAGAACCCAC |
| lin pcDNA3.1 promoter_RV | GCGTATATCTGGCCCGTAC |
| TK promoter for Gibson_FW | CGCGATGTACGGGCCAGATATACGCAAATGAGTCTTCGGACCTCG |
| TK promoter for Gibson_RV | GCAGTGGGTTCTCTAGTTAGCCAGATTAAGCGGGTCGCTGCAG |
| lin HA-FZD-XX_FW^4^ | GGCTCGAGTCTAGATGGAG |
| lin HA-FZD-XX_RV^4^ | ATCCCGCATAATCCGGCAC |
| M1 for M1-Nluc_FW | tgatgtgccggattatgcgggatccAACACTTCAGCCCCACCTG |
| M1 for M1-Nluc_RV | cacctccatctagACtcgagccGCATTGGCGGGAGGGAG |

# Table S5: Demographic information of the PHH donor used in this study.

| **Sex** | **Ethnicity** | **Age (years)** | **Cause of death** | **Relevant medical and social history** |
| --- | --- | --- | --- | --- |
| F | Hispanic | 30 | head trauma | smoker, narcotic dependency |

# Table S6: Taqman probes used in this study.

| **Gene** | **Probe ID** |
| --- | --- |
| *Tbp* (TATA-Box binding protein) | Hs00427620_m1 |
| *Lgr5* | Hs00969422_m1 |

# Experimental Section – Chemistry

**Abbreviations used in the following description of synthesis procedures:**

Boc *tert*-butoxycarbonyl

DCM dichloromethane

DIPEA diisopropylethylamine

DMF dimethylformamide

Et_3_N triethylamine

EtOAc ethyl acetate

EtOH ethanol

HATU 1-[Bis(dimethylamino)methylene]-1H-1,2,3-triazolo[4,5-b]pyridinium 3-oxide hexafluorophosphate

MeOH methanol

TFA trifluoroacetic acid

**General Information:**

Starting materials and chemical compounds in the synthesis described below were commercially available.

For compounds **9**, **10** and **52**

Exact masses of the final compounds were determined by high resolution mass spectrometry (HR-MS) using Electron Ionization Spray (ESI) with a timsTOF Pro instrument. The measured ^1^H and ^13^C NMR spectra were measured by a Bruker Avance 400 MHz or Avance 600 MHz and the chemical shifts were expressed in parts per million (ppm, δ) referenced to added tetramethylsilane (TMS). HPLC analysis was performed on an AGILENT 1200 series machine with a VMD detector. A standard system on a Zorbax Eclipse XDB-C8 (4.6 mm x 150 mm, 5 μm) column was used (acetonitrile/H_2_O + 0.1% HCOOH flow rate: 0.5 ml/min). Detection was performed at wavelengths of λ = 220 and 254 nm.

For compounds **11**, **14**, **15**, **17**, **18**, **19**, **21**, **23** and **24**

Mass spectrometry data are reported from liquid chromatography-mass spectrometry (LCMS) using ESI performed on an Agilent InfinityLab LC/MSD system. ^1^H and ^13^C NMR spectra were recorded on a Bruker Ascend 400 NMR spectrometer and chemical shifts are expressed in parts per million (ppm, δ) referenced to residual peaks from the deuterated solvent used. Preparative HPLC was carried out on a Shimadzu LC-20 system (XBridge C18 column, basic gradient using acetonitrile - H_2_O /ammonium hydroxide).

## Synthesis protocols for compounds 9, 10 and 52

Scheme 1. Synthesis of ligands **MRG26** and **MRG31-33.**

a) 4-(Pyridin-3-yl)benzaldehyde (**MRG21**), 3-(pyridin-4-yl)benzaldehyde (**MRG28**), NaBH_4_, MeOH, 2-2.5 h b) 3-Chlorbenzo[b]thiophene-2-carboxylic acid, HATU, DIPEA, DMF, 2.5-3 h c) DCM/TFA (1:1),1 h
d) Pyrazine-2-carbonyl chloride (**9** (**MRG32**)), furoyl chloride (**10** (**MRG33**), **52** (**MRG26**)), Et_3_N, DCM, 2 h.

***tert*-Butyl (4-((3-(pyridin-4-yl)benzyl)amino)cyclohexyl)carbamate (MRG28)**

*N*-Boc-1,4-diaminocyclohexane (777 mg; 3.626 mmol) and 3-(pyridin-4-yl)benzaldehyde (511.0 mg; 2.789 mmol) were dissolved in methanol (40 ml) and stirred for 1 h at room temperature. NaBH_4_ (1.09 g; 28.81 mmol; 10 eq.) was then added in portions at 0°C and the solution stirred for a further 1.5 h. After completion of the reaction, the mixture was taken up with saturated NaHCO_3_ solution (30 ml) and extracted with DCM (3 x 15 ml). The combined organic phases were dried over Na_2_SO_4_ and the solvent was finally removed. The crude product obtained was purified by flash chromatography using silica gel column (EtOAc/MeOH 90:10 + 1% Et_3_N). A white solid was obtained (1.06 g; 2.77 mmol; 99.3%). ^1^H NMR (400 MHz, CDCl_3_): δ ppm 8.59 (td, J = 4.6, 1.7 Hz, 2H), 7.54 – 7.31 (m, 6H), 4.29 (s, 1H), 3.81 (s, 2H), 3.39 (d, J = 25.4 Hz, 1H), 2.43 (tt, J = 10.8, 3.6 Hz, 1H), 2.04 – 1.88 (m, 4H), 1.37 (s, 9H), 1.23 – 0.99 (m, 5H).

***tert*-Butyl(4-(3-chloro-N-(3-(pyridin-4-yl)benzyl)benzo[*b*]thiophene-2-carboxamido)cyclohexyl)carbamate (MRG29)**

3-Chlorbenzo[*b*]thiophene-2-carboxylic acid (803.8 mg; 3.78 mmol; 1.5 eq.) and HATU (1.44 g; 3.78 mmol) were dissolved in DMF (25 ml) and DIPEA (0.98 ml; 5.76 mmol) was added while stirring. After 1 h, a solution of **MRG28** (957 mg; 2.51 mmol) in DMF (5 ml) was added and stirred for another 2 h at room temperature. The solvent was removed under reduced pressure. After addition of tert-butyl methyl ether the mixture was and washed with saturated NaHCO_3_ solution (3 x 30 ml). The organic phase was dried over Na_2_SO_4_ and the solvent was removed under vacuum. The crude product was purified over a silica gel column by flash chromatography (DCM/MeOH; gradient 0% MeOH - 10% MeOH). The product was obtained as a brown oil (1.26 g; 2.19 mmol; 87.3%). ^1^H NMR (400 MHz, CDCl_3_): δ ppm 8.61 (dd, J = 7.9, 3.4 Hz, 2H), 7.95 – 7.31 (m, 10H), 4.68 (d, J = 63.0 Hz, 2H), 3.72 (s, 1H), 3.24 (s, 1H), 1.92 – 1.51 (m, 6H), 1.35 (d, J = 26.2 Hz, 9H), 1.17 (dt, J = 13.0, 4.5 Hz, 2H), 1.00 – 0.85 (m, 1H).

***N*-(4-Aminocyclohexyl)-3-chloro-*N*-(3-(pyridin-4-yl)benzyl)benzo[*b*]thiophene-2-carboxamide (MRG30)**

**MRG29** (1.253 g; 2.177 mmol) was dissolved in DCM (32 ml)/TFA (8 ml) and allowed to stir for 2.5 h at room temperature. A sodium hydroxide solution was added and extracted with DCM (3 x 20 ml). The organic phase was finally dried over Na_2_SO_4_ and the solvent was removed. A brown viscous oil (1.00 g; 2.10 mmol; 96.7%) was obtained and used without further purification.

***N*-(4-(3-chloro-*N*-(3-(pyridin-4-yl)benzyl)benzo[*b*]thiophene-2-carboxamido)cyclohexyl)pyrazine-2-carboxamide (MRG32, 9)**

**MRG30** (51.3 mg; 0.11 mmol) and pyrazine-2-carbonyl chloride (19.9 mg; 0.14 mmol; 1.3 eq.) were dissolved in dry DCM (2ml) and Et_3_N (29 μl; 0.21 mmol; 2.0 eq.) was subsequently added with stirring. The mixture was then stirred for 2 h at room temperature. Saturated NaHCO_3_ solution (4 ml) was finally added to the reaction mixture and extracted with DCM (3 x 4 ml). The combined organic phases were dried over Na_2_SO_4_ and then the solvent was removed. The crude product was purified by preparative HPLC (Nucleodur column C-18; acetonitrile/H_2_O + TFA). The product obtained was a pink solid (5.3 mg; 0.01 mmol; 9.5%). ^1^H NMR (400 MHz, CDCl_3_) δ ppm 9.28 (s, 1H), 8.69 (d, *J* = 39.9 Hz, 3H), 8.40 (d, *J* = 38.7 Hz, 1H), 7.79 (d, *J* = 25.1 Hz, 4H), 7.68 – 7.27 (m, 7H), 4.74 (d, *J* = 60.3 Hz, 2H), 3.84 (d, *J* = 10.3 Hz, 2H), 2.14 – 1.60 (m, 6H), 1.19 (d, *J* = 7.7 Hz, 2H). ^13^C NMR (101 MHz, CDCl3): δ ppm 162.79, 161.63, 161.20, 146.37, 143.27, 141.36, 136.21, 128.90, 127.43, 126.75, 125.76, 124.66, 121.85, 121.55, 117.95, 57.75, 46.15, 44.05, 30.70, 29.51, 28.73, 18.75. HR-MS for C_32_H_28_ClN_5_O_2_S: calcd. m/z [M+H]^+1^ = 582.1730, found m/z [M+H]^+1^ = 582.1726

**4-(3-Chloro-*N*-(3-(pyridin-4-yl)benzyl)benzo[*b*]thiophene-2-carboxamido)cyclohexyl)furan-2-carboxamide (MRG33, 10)**

**MRG30** (51.2 mg; 0.11 mmol) and furoyl chloride (15.6 μl; 0.158 mmol) were dissolved in dry DCM (2 ml) and Et_3_N (33.5 μl; 0.242 mmol) was added while stirring at room temperature. The reaction mixture was continuously stirred at room temperature for 2 h. Saturated NaHCO_3_ solution (4 ml) was then added and extracted with DCM (3 x 3 ml). The organic phase was dried over Na_2_SO_4_ and the solvent was removed. The crude product was purified by preparative HPLC (Nucleodur C-18 column; acetonitrile/H_2_O + TFA). A white solid was obtained (23.3 mg; 0.041 mmol; 39%). ^1^H NMR (400 MHz, CDCl_3_) δ ppm 8.77 (s, 2H), 7.90 (s, 1H), 7.54 (ddt, *J* = 45.0, 43.7, 11.3 Hz, 9H), 6.99 (s, 1H), 6.36 (d, *J* = 25.2 Hz, 1H), 6.02 (t, *J* = 35.6 Hz, 1H), 4.73 (d, *J* = 56.0 Hz, 2H), 3.78 (s, 2H), 1.86 (dt, *J* = 57.4, 28.4 Hz, 6H), 1.21 (t, *J* = 59.1 Hz, 2H). ^13^C NMR (151 MHz, CDCl3): δ ppm 162.84, 156.58, 146.69, 142.75, 139.00, 136.12, 128.98, 125.73, 125.04, 124.59, 122.50, 121.56, 117.83, 113.05, 111.17, 57.74, 45.72, 44.01, 31.10, 30.82, 29.66, 29.49. HR-MS for C_32_H_28_ClN_3_O_3_S: calcd. m/z [M+H]^+1^ = 570.1613, found m/z [M+H]^+1^ = 570.1616.

***tert*-Butyl (4-((4-(pyridin-3-yl)benzyl)amino)cyclohexyl)carbamate (MRG21)**

4-(Pyridin-3-yl)benzaldehyde (601.5 mg; 3.283 mmol) and *trans*-*N*-Boc-1,4-diaminocyclohexane (915.8 mg; 4.273 mmol) were dissolved in methanol (65 ml) and stirred at room temperature for 1 h. The reaction mixture was then cooled to 0 °C and NaBH_4_ (1.238 g; 32.73 mmol; 10 eq.) was added slowly and allowed to stir for another hour. After the addition of saturated NaHCO_3_ solution (70 ml), the solution was extracted with DCM (3 x 50 ml). The combined organic phases were dried over Na_2_SO_4_ and the solvent was removed *under vacuum*. A white solid was obtained (1.250 g; 3.278 mmol; 99.8%). ^1^H NMR (400 MHz, CDCl_3_): δ ppm 8.84 (dd, J = 2.3, 0.8 Hz, 1H), 8.60 – 8.54 (m, 1H), 7.89 – 7.84 (m,1H), 7.64 – 7.33 (m, 5H), 4.38 (s, 1H), 3.87 (d, J = 5.7 Hz, 2H), 3.46 (d, J = 21.4 Hz, 1H), 2.54 – 2.41 (m, 1H), 2.06 – 1.83 (m, 4H), 1.44 (s, 9H), 1.30 – 1.07 (m, 5H).

**tert-Butyl(4-(3-chloro-N-(4-(pyridin-3-yl)benzyl)benzo[*b*]thiophene-2-carboxamido)cyclohexyl)carbamate (MRG23)**

3-Chlorbenzo[*b*]thiophene-2-carboxylic acid (725.8 mg; 3.413 mmol; 1.3 eq.) and HATU (1.296 g; 3.41 mmol; 1.3 eq.) were dissolved in DMF (40 ml) and DIPEA (0.8 ml; 5.246 mmol; 2.0 eq.) was added with stirring. After stirring for 1 h, MRG21 (1.0 g; 2.62 mmol) was then added and the reaction mixture was stirred for another 1.5 h. The solvent was then removed, redissolved in DCM, and washed with saturated NaHCO_3_ solution three times. The obtained organic phase was finally dried over Na_2_SO_4_ and the solvent was removed under vacuum. After purification over a silica gel column by flash chromatography (EtOAc/isohexane 1:1 + 0.1% Et_3_N), a light-yellow solid was obtained (592.8 mg; 1.03 mmol; 39.3%). ^1^H NMR (400 MHz, CDCl_3_): δ ppm 9.00 – 8.54 (m, 2H), 8.01 – 7.34 (m, 10H), 4.90 – 4.63 (m, 2H), 3.33 (s, 1H), 1.98 – 1.57 (m, 5H), 1.41 (s, 9H), 1.31 – 0.93 (m, 5H).

***tert*-Butyl(4-(3-chloro-N-(4-(pyridin-3-yl)benzyl)benzo[*b*]thiophene-2-carboxamido)cyclohexyl)carbamate (MRG24)**

**MRG23** (0.50 g; 0.87 mmol) was dissolved in a DCM/TFA (1:1; 16 ml) solution and stirred at room temperature for 1 h. The reaction mixture was then taken up with a NaHCO_3_ solution (pH 12; 15 ml) and extracted with DCM (3 x 5ml). The organic phase was dried over Na_2_SO_4_ and the solvent was removed. A yellow-brown oil was obtained and used without further purification (114 mg; 0.240 mmol; 28%).

***N*-(4-(3-Chloro-N-(4-(pyridin-3-yl)benzyl)benzo[*b*]thiophene-2-carboxamido)cyclohexyl)furan-2-carboxamide (MRG26, 52)**

**MRG24** (31 mg; 0.063 mmol) was added to anhydrous DCM (1ml) in a microwave tube under nitrogen atmosphere. Furoyl chloride (81 μl; 0.082 mmol) was then added and slowly Et_3_N (26.2 μl; 0.185 mmol) was added dropwise. The solution was stirred for additional 2 h at room temperature. The product was purified by preparative HPLC using a Nucleodur C18 column (acetonitrile/H_2_O + TFA). The product obtained was a white solid (3.5 mg; 0.0062 mmol; 9.8%). ^1^H NMR (400 MHz, CDCl_3_) δ ppm 9.01 (s, 1H), 8.66 (s, 1H), 8.31 (s, 1H), 7.95 – 7.22 (m, 10H), 6.96 (d, *J* = 26.0 Hz, 1H), 6.39 (s, 1H), 6.04 (d, *J* = 56.6 Hz, 1H), 4.72 (d, *J* = 51.5 Hz, 2H), 3.77 (d, *J* = 6.7 Hz, 2H), 1.78 (dd, *J* = 98.7, 44.3 Hz, 6H), 1.19 (dd, *J* = 61.3, 40.8 Hz, 2H). ^13^C NMR (151 MHz, CDCl3): δ ppm 162.67, 156.54, 146.72, 142.68, 141.13, 138.94, 136.30, 127.23, 126.34, 125.55, 124.51, 121.58, 121.28, 113.11, 111.11, 57.69, 45.72, 43.83, 31.01, 30.73, 29.52, 29.48. HR-MS for C_32_H_28_ClN_3_O_3_S: calcd. m/z [M+H]^+1^ = 570.1613, found m/z [M+H]^+1^ = 570.1617.

# Synthesis protocols for compounds 11, 14, 15, 17, 18, 19, 21, 23 and 24

**Preparation of compounds 11, 14, 15, 17, 18, 19, 21 and 23**

Scheme 1. Synthetic route to compounds **11, 14, 15, 17, 18, 19, 21 and 23**.

a) 3-Bromobenzaldehyde, NaBH(OAc)_3_, EtOH, 20 h; b) 3-Chlorobenzo[*b*]thiophene-2-carboxylic acid, HATU, Et_3_N, DMF, 16 h; c) 4-Pyridinylboronic acid, Pd(PPh_3_)_4_, K_2_CO_3_, 1,4-dioxane/H_2_O (4:1), 90°C, 2 h; d) TFA/DCM (1:1), 0.5 h; e) RCO_2_H, HATU, Et_3_N, DMF, 16 h.

***tert*-Butyl ((1r,4r)-4-((3-bromobenzyl)amino)cyclohexyl)carbamate (DD434601)**

A mixture of 3-bromobenzaldehyde (1.85 g, 10.0 mmol), *tert*-butyl ((1r,4r)-4-aminocyclohexyl)carbamate (2.14 g, 10.0 mmol) and sodium triacetoxyborohydride (6.36 g, 30.0 mmol) in ethanol (50 mL) was stirred at room temperature for 20 h. The reaction mixture was quenched with saturated aqueous NaHCO_3_ and concentrated to dryness. The residue was taken up in saturated aqueous NaHCO_3_ and extracted with EtOAc (3 times). The organic layers were dried over Na_2_SO_4_, filtered and concentrated and the residue was purified by silica gel flash chromatography (5-10% MeOH in DCM) to give the title compound. Yield: 1.35 g (35%). LCMS [M+H]^+^ 383; ^1^H NMR (400 MHz, DMSO-*d*_6_) δ ppm 7.51 - 7.55 (m, 1H), 7.36 - 7.41 (m, 1H), 7.29 - 7.34 (m, 1H), 7.22 - 7.28 (m, 1H), 6.55 - 6.71 (m, 1H), 3.69 (s, 2H), 3.05 - 3.23 (m, 1H), 2.16 - 2.30 (m, 1H), 1.98 - 2.11 (m, 1H), 1.80 - 1.91 (m, 2H), 1.66 - 1.78 (m, 2H), 1.36 (s, 9H), 0.94 - 1.17 (m, 4H); ^13^C NMR (101 MHz, DMSO-*d*_6_) δ ppm 154.8, 144.7, 130.4, 130.2, 129.1, 126.8, 121.5, 77.3, 54.9, 49.4, 49.1, 31.6, 31.2, 28.3.

***tert*-Butyl ((1r,4r)-4-(N-(3-bromobenzyl)-3-chlorobenzo[b]thiophene-2-carboxamido)cyclohexyl)carbamate (DD434801)**

To a solution of 3-chlorobenzothiophene-2-carboxylic acid (0.21 g, 1.0 mmol), *tert*-butyl *N*-[4-[(3-bromophenyl)methylamino]cyclohexyl]carbamate (**DD434601**) (0.38 g, 1.0 mmol) and HATU (0.49 g, 1.3 mmol) in DMF (3 mL). The mixture was treated with Et_3_N (0.28 mL, 2.0 mmol) and stirred at room temperature for 16 h. The reaction mixture was concentrated to dryness and the residue was taken up in EtOAc and extracted with 1M aqueous HCl (twice) and washed with H_2_O. The organic layers were dried over Na_2_SO_4_, filtered and concentrated. The crude material was purified by silica gel flash chromatography (10-50% EtOAc in iso-hexane) to give the title compound. Yield: 0.51 g (88%). LCMS [M-^t^Bu+H]^+^ 521; ^1^H NMR (400 MHz, DMSO-*d*_6_, 80°C) δ ppm 8.06 - 8.13 (m, 1H), 7.85 - 7.91 (m, 1H), 7.52 - 7.64 (m, 3H), 7.41 - 7.46 (m, 1H), 7.34 - 7.40 (m, 1H), 7.27 - 7.33 (m, 1H), 6.17 (br s, 1H), 4.71 (s, 2H), 3.60 - 3.86 (m, 1H), 3.11 - 3.24 (m, 1H), 1.62 - 1.84 (m, 6H), 1.34 (s, 9H), 0.99 - 1.15 (m, 2H); ^13^C NMR (101 MHz, DMSO-*d*_6_, 80°C) δ ppm 162.2, 154.4, 141.1, 136.2, 134.5, 130.0, 129.3, 126.4, 125.6, 125.5, 123.0, 121.5, 121.2, 117.2, 77.1, 40.1, 39.9, 39.7, 39.5, 39.3, 39.1, 38.9, 31.1, 29.1, 27.9.

***N*-((1*r*,4*r*)-4-Aminocyclohexyl)-3-chloro-*N*-(3-(pyridin-4-yl)benzyl)benzo[*b*]thiophene-2-carboxamide (DJ507601)**

A mixture of *tert*-butyl ((1*r*,4*r*)-4-(*N*-(3-bromobenzyl)-3-chlorobenzo[*b*]thiophene-2-carboxamido)cyclohexyl)carbamate (**DD434801**) (0.43 g, 0.75 mmol), 4-pyridinylboronic acid (0.11 g, 0.90 mmol), Pd(PPh_3_)_4_ (22 mg, 0.019 mmol) and K_2_CO_3_ (0.21 g, 1.5 mmol) in 1,4-dioxane (12 mL) and H_2_O (3 mL) was heated at 90°C for 2 h. The reaction mixture was concentrated and purified by silica gel flash chromatography (20% EtOAc in iso-hexane). The obtained Boc-protected material was stirred in TFA/DCM (1:1) for 0.5 h, concentrated and further purified by preparative HPLC to give the title compound. Yield: 0.23 g (64%). LCMS [M +H]^+^ 476; ^1^H NMR (400 MHz, DMSO-*d*_6_, 80°C) δ ppm 8.60 - 8.68 (m, 2H), 8.04 - 8.11 (m, 1H), 7.84 - 7.90 (m, 1H), 7.40 - 7.76 (m, 8H), 4.78 (s, 2H), 3.70 - 3.94 (m, 1H), 2.39 - 2.48 (m, 1H), 1.62 - 1.82 (m, 6H), 0.84 - 1.05 (m, 2H); ^13^C NMR (101 MHz, DMSO-*d*_6_, 80°C) δ ppm 162.2, 149.8, 146.6, 139.3, 137.0, 136.2, 134.5, 130.7, 128.8, 127.2, 126.3, 125.5, 124.9, 124.8, 123.0, 121.5, 120.7, 117.1, 48.6, 34.9, 29.3.

**General method (A) for the preparation of amides 11, 14, 15, 17, 18, 19, 21 and 23**

A mixture of *N*-((1*r*,4*r*)-4-aminocyclohexyl)-3-chloro-*N*-(3-(pyridin-4-yl)benzyl)benzo[*b*]thiophene-2-carboxamide (**DJ507601**)(19 mg, 0.040 mmol),the respective

carboxylic acid (0.044 mmol) and HATU (20 mg, 0.052 mmol) in DMF (2 mL) was treated with Et_3_N (11 µL, 0.080 mmol) and stirred at room temperature for 16 h. Purification by preparative HPLC gave the corresponding title compound.

***N*-((1*r*,4*r*)-4-(3-Acetamidobenzamido)cyclohexyl)-3-chloro-*N*-(3-(pyridin-4-yl)benzyl)benzo[*b*]thiophene-2-carboxamide (DJ503701, 11)**

Prepared from 3-acetamidobenzoic acid using general method A. Yield: 18 mg (71%). LCMS [M+H]^+^ 637; ^1^H NMR (400 MHz, DMSO-*d*_6_, 80°C) δ ppm 9.77 (br s, 1H), 8.63 - 8.72 (m, 2H), 8.05 - 8.13 (m, 1H), 7.85 - 7.93 (m, 2H), 7.64 - 7.83 (m, 6H), 7.47 - 7.64 (m, 4H), 7.35 - 7.41 (m, 1H), 7.23 - 7.31 (m, 1H), 4.85 (s, 2H), 3.68 - 3.99 (m, 2H), 2.02 (s, 3H), 1.72 - 1.92 (m, 6H), 1.19 - 1.41 (m, 2H); ^13^C NMR (101 MHz, DMSO-*d*_6_, 80°C) δ ppm 167.8, 165.1, 162.2, 149.1, 147.4, 139.4, 138.9, 136.7, 136.2, 135.2, 134.5, 130.6, 128.8, 127.8, 127.5, 126.4, 125.6, 125.1, 125.0, 123.0, 121.5, 121.3, 121.0, 118.3, 117.1, 46.6, 30.8, 29.3, 23.4.

**3-Chloro-*N*-((1*r*,4*r*)-4-(3-propionamidobenzamido)cyclohexyl)-*N*-(3-(pyridin-4-yl)benzyl)benzo[*b*]thiophene-2-carboxamide (DJ508501, 14)**

Prepared from 3‐propanamidobenzoic acid using general method A. Yield: 15 mg (58%). LCMS [M+H]^+^ 651; ^1^H NMR (400 MHz, DMSO-*d*_6_, 80°C) δ ppm 9.68 (br s, 1H), 8.59 - 8.69 (m, 2H), 8.04 - 8.14 (m, 1H), 7.85 - 7.93 (m, 2H), 7.46 - 7.82 (m, 10H), 7.34 - 7.40 (m, 1H), 7.22 - 7.31 (m, 1H), 4.84 (s, 2H), 3.69 - 3.84 (m, 1H), 2.30 (q, *J* = 7.50 Hz, 2H), 1.70 - 1.92 (m, 6H), 1.23 - 1.34 (m, 2H), 1.09 (t, *J*=7.57 Hz,3 H); ^13^C NMR (101 MHz, DMSO-*d*_6_, 80°C) δ ppm 171.6, 162.2, 159.8, 149.8, 138.9, 137.0, 135.2, 134.5, 128.8, 127.8, 127.3, 126.4, 125.6, 124.9, 124.2, 123.0, 121.5, 120.7, 118.3, 117.1, 30.8, 29.1, 9.0.

**3-Chloro-*N*-((1*r*,4*r*)-4-(3-isobutyramidobenzamido)cyclohexyl)-*N*-(3-(pyridin-4-yl)benzyl)benzo[*b*]thiophene-2-carboxamide (DJ508401, 15)**

Prepared from 3-isobutyramidobenzoic acid using general method A. Yield: 8 mg (30%). LCMS [M+H]^+^ 665; ^1^H NMR (400 MHz, DMSO-*d*_6_, 80°C) δ ppm 9.62 (br s, 1H), 8.59 - 8.68 (m, 2H), 8.05 - 8.15 (m, 1H), 7.85 - 7.95 (m, 2H), 7.45 - 7.82 (m, 10H), 7.34 - 7.40 (m, 1H), 7.23 - 7.32 (m, 1H), 4.84 (s, 2H), 3.65 - 3.96 (m, 2H), 2.53 - 2.62 (m, 1H), 1.70 - 1.93 (m, 6H), 1.21 - 1.38 (m, 2H), 1.10 (d, *J* = 6.75 Hz, 6H); ^13^C NMR (101 MHz, DMSO-*d*_6_, 80°C) δ ppm 174.9, 165.1, 162.2, 149.8, 139.0, 137.0, 136.2, 135.2, 134.5, 130.6, 128.8, 127.8, 127.3, 126.4, 125.6, 124.9, 123.0, 121.5, 121.0, 120.8, 118.4, 117.1, 46.6, 34.5, 30.8, 23.6, 18.9.

***N*-(3-(((1*r*,4*r*)-4-(3-Chloro-*N*-(3-(pyridin-4-yl)benzyl)benzo[*b*]thiophene-2-carboxamido)cyclohexyl)-carbamoyl)phenyl)-1-methylpiperidine-4-carboxamide (DJ508701, 17)**

Prepared from 3‐(1‐methylpiperidine‐4‐amido)benzoic acid using general method A. Yield: 12 mg (42%). LCMS [M+H]^+^ 720; ^1^H NMR (400 MHz, DMSO-*d*_6_, 80°C) δ ppm 9.66 (br s, 1H), 8.60 - 8.68 (m, 2H), 8.05 - 8.13 (m, 1H), 7.85 - 7.95 (m, 2H), 7.44 - 7.81 (m, 10H), 7.34 - 7.40 (m, 1H), 7.23 - 7.31 (m, 1H), 4.84 (s, 2H), 3.67 - 3.93 (m, 2H), 2.74 - 2.84 (m, 2H), 2.21 - 2.32 (m, 1H), 2.17 (s, 3H), 1.59 - 1.96 (m, 12H), 1.19 - 1.38 (m, 2H); ^13^C NMR (101 MHz, DMSO-*d*_6_, 80°C) δ ppm 173.2, 165.1, 162.2, 149.8, 146.7, 138.9, 137.0, 136.2, 135.2, 134.5, 128.8, 127.8, 127.3, 126.4, 125.6, 124.9, 123.0, 121.5, 121.4, 121.0, 120.7, 118.3, 117.1, 54.4, 45.6, 42.0, 30.8, 28.0.

**3-Chloro-*N*-((1*r*,4*r*)-4-(3-(2-(4-methylpiperazin-1-yl)acetamido)benzamido)cyclohexyl)-*N*-(3-(pyridin-4-yl)benzyl)benzo[*b*]thiophene-2-carboxamide (DQ090601, 18)**

Prepared from 3-(2-(4-methylpiperazin-1-yl)acetamido)benzoic acid using general method A. Yield: 18 mg (61%). LCMS [M+H]^+^ 735; ^1^H NMR (400 MHz, DMSO-*d*_6_, 80°C) δ ppm 9.53 (br s, 1H), 8.60 - 8.69 (m, 2H), 8.05 - 8.13 (m, 1H), 7.86 - 7.92 (m, 2H), 7.47 - 7.82 (m, 10H), 7.38 - 7.45 (m, 1H), 7.26 - 7.35 (m, 1H), 4.84 (s, 2H), 3.70 - 3.92 (m, 2H), 3.09 (s, 2H), 2.52 - 2.57 (m, 4H), 2.34 - 2.42 (m, 4H), 2.18 (s, 3H), 1.71 - 1.94 (m, 6H), 1.19 - 1.39 (m, 2H); ^13^C NMR (101 MHz, DMSO-*d*_6_, 80°C) δ ppm 168.0, 162.2, 149.8, 146.7, 138.1, 137.0, 136.2, 135.2, 134.5, 128.8, 127.9, 127.3, 126.4, 125.6, 125.0, 124.9, 123.0, 121.7, 121.5, 120.7, 118.5, 117.1, 61.3, 54.2, 52.3, 45.2, 30.8.

**3-Chloro-*N*-((1*r*,4*r*)-4-(3-(2-morpholinoacetamido)benzamido)cyclohexyl)-*N*-(3-(pyridin-4-yl)benzyl)benzo[*b*]thiophene-2-carboxamide (DQ090801, 19)**

Prepared from 3-(2-morpholinoacetamido)benzoic acid using general method A. Yield: 10 mg (35%). LCMS [M+H]^+^ 722; ^1^H NMR (400 MHz, DMSO-*d*_6_, 80°C) δ ppm 9.83 (br s, 1H), 8.58 - 8.72 (m, 2H), 8.03 - 8.17 (m, 1H), 7.48 - 7.97 (m, 11H), 7.38 - 7.47 (m, 1H), 7.26 - 7.37 (m, 1H), 4.84 (s, 2H), 3.72 - 3.92 (m, 2H), 3.65 - 3.72 (m, 4H), 3.35 (s, 2H), 2.62 - 2.79 (m, 4H), 1.68 - 1.98 (m, 6H), 1.12 - 1.48 (m, 2H); ^13^C NMR (101 MHz, DMSO-*d*_6_) δ ppm 164.9, 162.2, 149.6, 146.9, 139.3, 137.9, 137.0, 136.2, 135.2, 134.5, 130.6, 128.8, 127.9, 127.3, 126.4, 125.6, 125.0, 124.9, 123.0, 121.8, 121.7, 121.5, 120.8, 118.7, 117.1, 65.0, 60.5, 52.6, 46.6, 30.8, 29.2.

**3-Chloro-*N*-((1*r*,4*r*)-4-(3-chloro-*N*-(3-(pyridin-4-yl)benzyl)benzo[*b*]thiophene-2-carboxamido)cyclohexyl)isonicotinamide (DQ090001, 21)**

Prepared from 3-chloropyridine-4-carboxylic acid using general method A. Yield: 16 mg (65%). LCMS [M+H]^+^ 615; ^1^H NMR (400 MHz, DMSO-*d*_6_, 80°C) δ ppm 8.58 - 8.69 (m, 3H), 8.47 - 8.54 (m, 1H), 8.04 - 8.19 (m, 2H), 7.84 - 7.91 (m, 1H), 7.44 - 7.78 (m, 8H), 7.29 - 7.36 (m, 1H), 4.83 (s, 2H), 3.65 - 3.99 (m, 2H), 1.70 - 1.99 (m, 6H), 1.10 - 1.35 (m, 2H); ^13^C NMR (101 MHz, DMSO-*d*_6_, 80°C) δ ppm 163.0, 162.2, 149.8, 148.9, 147.7, 143.2, 137.0, 136.2, 134.5, 128.8, 127.3, 127.0, 126.4, 125.6, 125.0, 124.9, 123.0, 122.2, 121.5, 120.7, 117.2, 46.9, 30.7, 28.9.

**6-Amino-*N*-((1*r*,4*r*)-4-(3-chloro-*N*-(3-(pyridin-4-yl)benzyl)benzo[*b*]thiophene-2-carboxamido)cyclohexyl)pyridazine-3-carboxamide (DQ090901, 23)**

Prepared from 6-aminopyridazine-3-carboxylic acid using general method A. Yield: 15 mg (63%). LCMS [M+H]^+^ 597; ^1^H NMR (400 MHz, DMSO-*d*_6_, 80°C) δ ppm 8.61 - 8.68 (m, 2H), 8.03 - 8.13 (m, 2H), 7.84 - 7.92 (m, 1H), 7.41 - 7.78 (m, 9H), 6.82 (d, *J* = 9.13 Hz, 1H), 6.62 (s, 2H), 4.78 - 4.87 (m, 2H), 3.67 - 4.06 (m, 2H), 1.72 - 1.91 (m, 6H), 1.33 - 1.52 (m, 2H); ^13^C NMR (101 MHz, DMSO-*d*_6_, 80°C) δ ppm 162.2, 162.1, 161.2, 149.8, 146.7, 144.6, 139.3, 137.0, 136.2, 134.5, 130.6, 128.8, 127.2, 126.3, 125.8, 125.5, 125.0, 124.9, 123.0, 121.5, 120.7, 117.2, 113.1, 46.3, 30.7, 29.1.

**Preparation of compound 24**

Scheme 2. Synthetic route to compound **24**.

a) Benzofuran-2-ylboronic acid, glyoxylic acid, DCM, 16 h; b) 3-Aminobiphenyl, HATU, Et_3_N, DMF, 16 h;
c) TFA/DCM (1:1), 0.5 h; e) 3-Acetamidobenzoic acid, HATU, Et_3_N, DMF, 16 h.

**2-(Benzofuran-2-yl)-2-(4-((*tert*-butoxycarbonyl)amino)piperidin-1-yl)acetic acid (DJ508301)**

A mixture of benzofuran-2-ylboronic acid (97 mg, 0.60 mmol), *tert*-butyl *N*-(4-piperidyl)carbamate (120 mg, 0.60 mmol) and glyoxylic acid monohydrate (55 mg, 0.60 mmol) in DCM (4 mL) was stirred at room temperature for 16 h. The white precipitate was collected by vacuum filtration, washed with a small volume of DCM and dried under vacuum to give the title compound. Yield: 137 mg (61%). LCMS [M+H]^+^ 375; ^1^H NMR (400 MHz, methanol-*d*_4_) δ ppm 7.62 - 7.67 (m, 1H), 7.49 - 7.55 (m, 1H), 7.34 - 7.39 (m, 1H), 7.24 - 7.31 (m, 1H), 7.12 (s, 1H), 4.93 (s, 1H), 3.52 - 3.68 (m, 2H), 3.33 - 3.39 (m, 1H), 3.02 - 3.23 (m, 2H), 2.01 - 2.17 (m, 2H), 1.73 - 1.91 (m, 2H), 1.42 (s, 9H); ^13^C NMR (101 MHz, DMSO-*d*_6_) δ ppm 167.6, 154.8, 154.3, 151.9, 127.6, 124.5, 123.0, 121.3, 111.2, 107.4, 77.6, 65.8, 48.9, 46.3, 30.8, 28.2.

***N*-([1,1'-Biphenyl]-3-yl)-2-(4-aminopiperidin-1-yl)-2-(benzofuran-2-yl)acetamide (DJ508901)**

To a solution of 2-(benzofuran-2-yl)-2-(4-((*tert*-butoxycarbonyl)amino)piperidin-1-yl)acetic acid (**DJ508301**) (75 mg, 0.20 mmol), 3-aminobiphenyl (41 mg, 0.24 mmol) and HATU (99 mg, 0.26 mmol) in DMF (3 mL) were added. The mixture was treated with Et_3_N (56 µL, 0.40 mmol) and stirred at room temperature for 16 h. The reaction mixture was concentrated and purified by silica gel flash chromatography (1-10% MeOH in DCM). The obtained Boc-protected material was stirred in TFA/DCM (1:1) for 0.5 h, concentrated and purified by preparative HPLC to give the title compound. Yield: 55 mg (79%). LCMS [M+H]^+^ 426; ^1^H NMR (400 MHz, DMSO-*d*_6_, 80°C) δ ppm 9.98 (br s, 1H), 7.93 - 7.99 (m, 1H), 7.58 - 7.67 (m, 4H), 7.52 - 7.57 (m, 1H), 7.34 - 7.51 (m, 5H), 7.20 - 7.32 (m, 2H), 6.89 - 6.96 (m, 1H), 4.64 (s, 1H), 2.81 - 2.98 (m, obscured by water peak, 2H), 2.57 - 2.66 (m, 1H), 2.41 - 2.48 (m, 1H), 2.17 - 2.27 (m, 1H), 1.70 - 1.83 (m, 2H), 1.35 - 1.51 (m, 2H); ^13^C NMR (101 MHz, DMSO-*d*_6_, 80°C) δ ppm 166.6, 154.0, 152.8, 140.6, 139.7, 138.5, 128.9, 128.4, 127.4, 127.1, 126.2, 123.8, 122.4, 121.8, 120.7, 118.4, 117.7, 110.6, 106.4, 67.2, 49.5, 48.3, 47.4, 34.8, 34.7.

***N*-(1-(2-([1,1'-Biphenyl]-3-ylamino)-1-(benzofuran-2-yl)-2-oxoethyl)piperidin-4-yl)-3-acetamidobenzamide (DJ509001, 24)**

A mixture of *N*-([1,1'-biphenyl]-3-yl)-2-(4-aminopiperidin-1-yl)-2-(benzofuran-2-yl)acetamide (**DJ508901**) (21 mg, 0.050 mmol), 3-acetamidobenzoic acid (11 mg, 0.060 mmol) and HATU (25 mg, 0.065 mmol) in DMF (2 mL) was treated with Et_3_N (14 µL, 0.10 mmol) and stirred at room temperature for 16 h. The mixture was purified by preparative HPLC to give the corresponding title compound. Yield: 15 mg (51%). LCMS [M+H]^+^ 587; ^1^H NMR (400 MHz, DMSO-*d*_6_, 80°C) δ ppm 10.02 (s, 1H), 9.80 (br s, 1H), 7.88 - 7.99 (m, 3H), 7.70 - 7.76 (m, 1H), 7.60 - 7.69 (m, 4H), 7.53 - 7.59 (m, 1H), 7.19 - 7.51 (m, 9H), 6.93 - 6.97 (m, 1H), 4.68 (s, 1H), 3.69 - 3.83 (m, 1H), 2.92 - 3.04 (m, 2H), 2.53 - 2.62 (m, 1H), 2.26 - 2.36 (m, 1H), 2.05 (s, 3H), 1.82 - 1.94 (m, 2H), 1.67 - 1.82 (m, 2H); ^13^C NMR (101 MHz, DMSO-*d*_6_, 80°C) δ ppm 167.9, 166.5, 165.5, 154.0, 152.8, 140.6, 139.7, 138.9, 138.5, 135.4, 128.9, 128.4, 127.9, 127.4, 127.1, 126.3, 123.9, 122.4, 121.8, 121.3, 121.2, 120.7, 118.4, 118.3, 117.7, 110.6, 106.4, 67.1, 50.0, 48.7, 46.4, 31.3, 31.1, 23.4.

# Analytical data for compounds 9, 10, 11, 14, 15, 17, 18, 19, 21, 23, 24 and 52

***N*-(4-(3-chloro-*N*-(3-(pyridin-4-yl)benzyl)benzo[*b*]thiophene-2-carboxamido)cyclohexyl)pyrazine-2-carboxamide (MRG32, compound 9)**


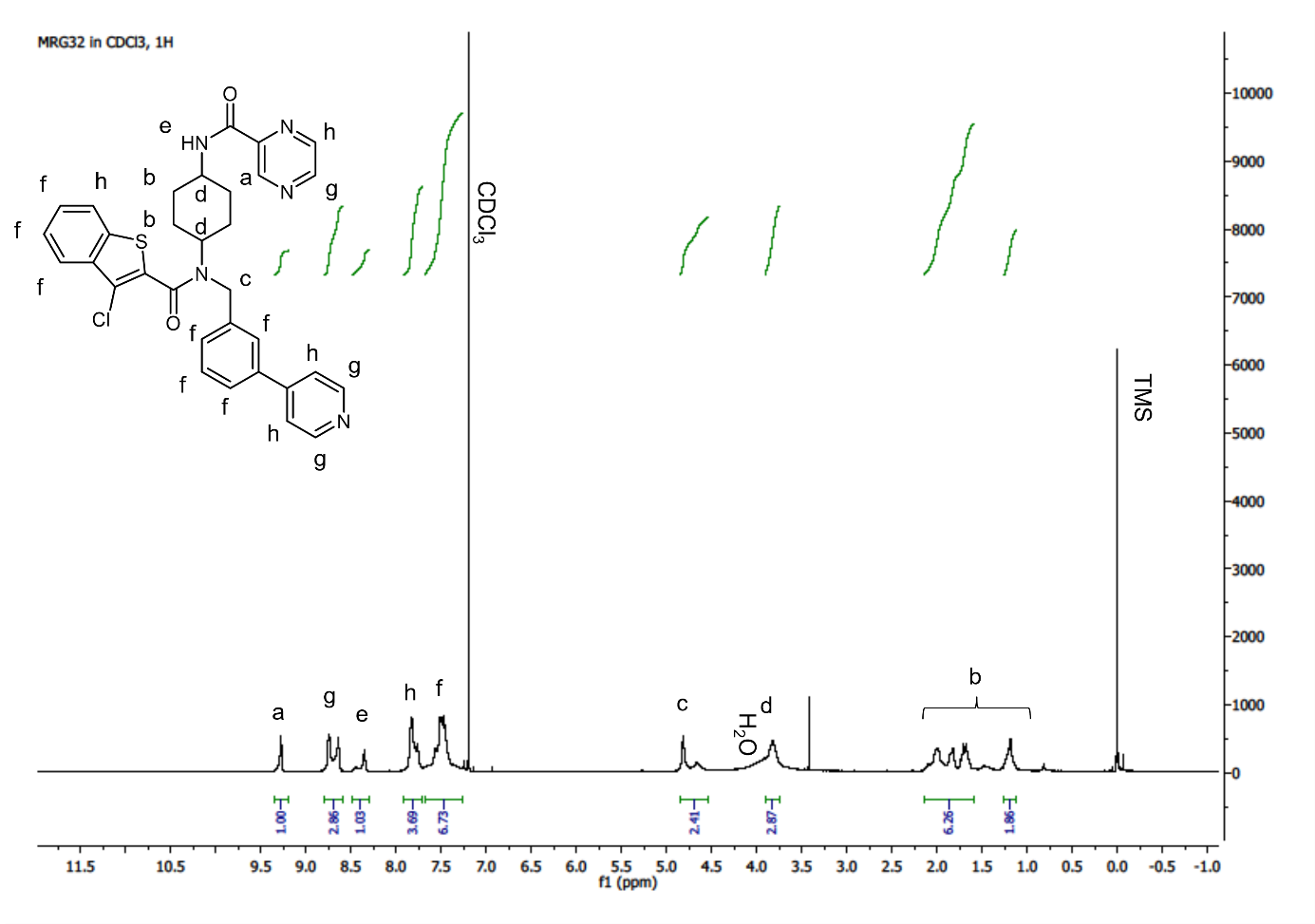


**4-(3-Chloro-*N*-(3-(pyridin-4-yl)benzyl)benzo[*b*]thiophene-2-carboxamido)cyclohexyl)furan-2-carboxamide (MRG33, compound 10)**


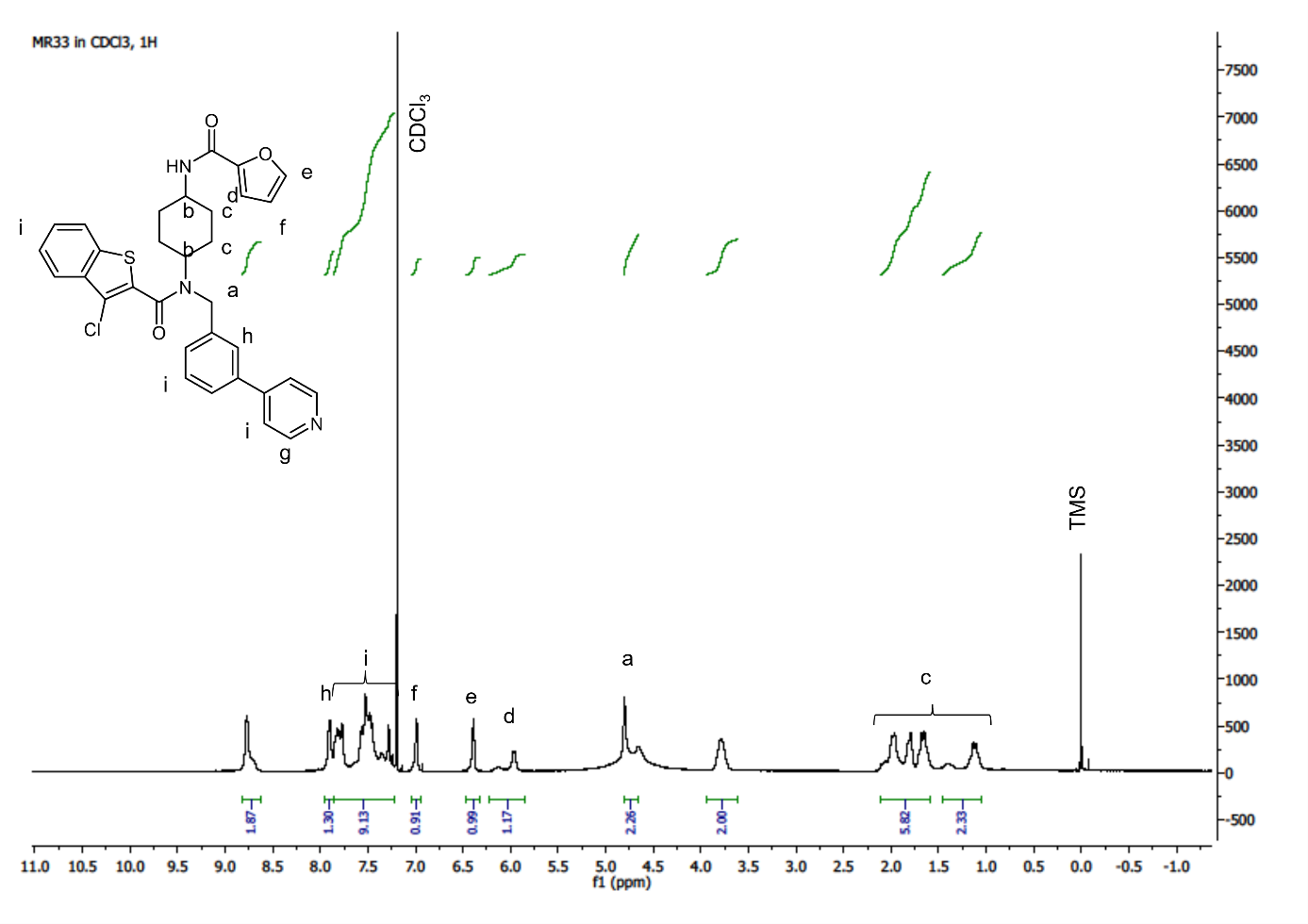


***N*-((1*r*,4*r*)-4-(3-Acetamidobenzamido)cyclohexyl)-3-chloro-*N*-(3-(pyridin-4-yl)benzyl)benzo[*b*]thiophene-2-carboxamide (DJ503701, compound 11)**

**
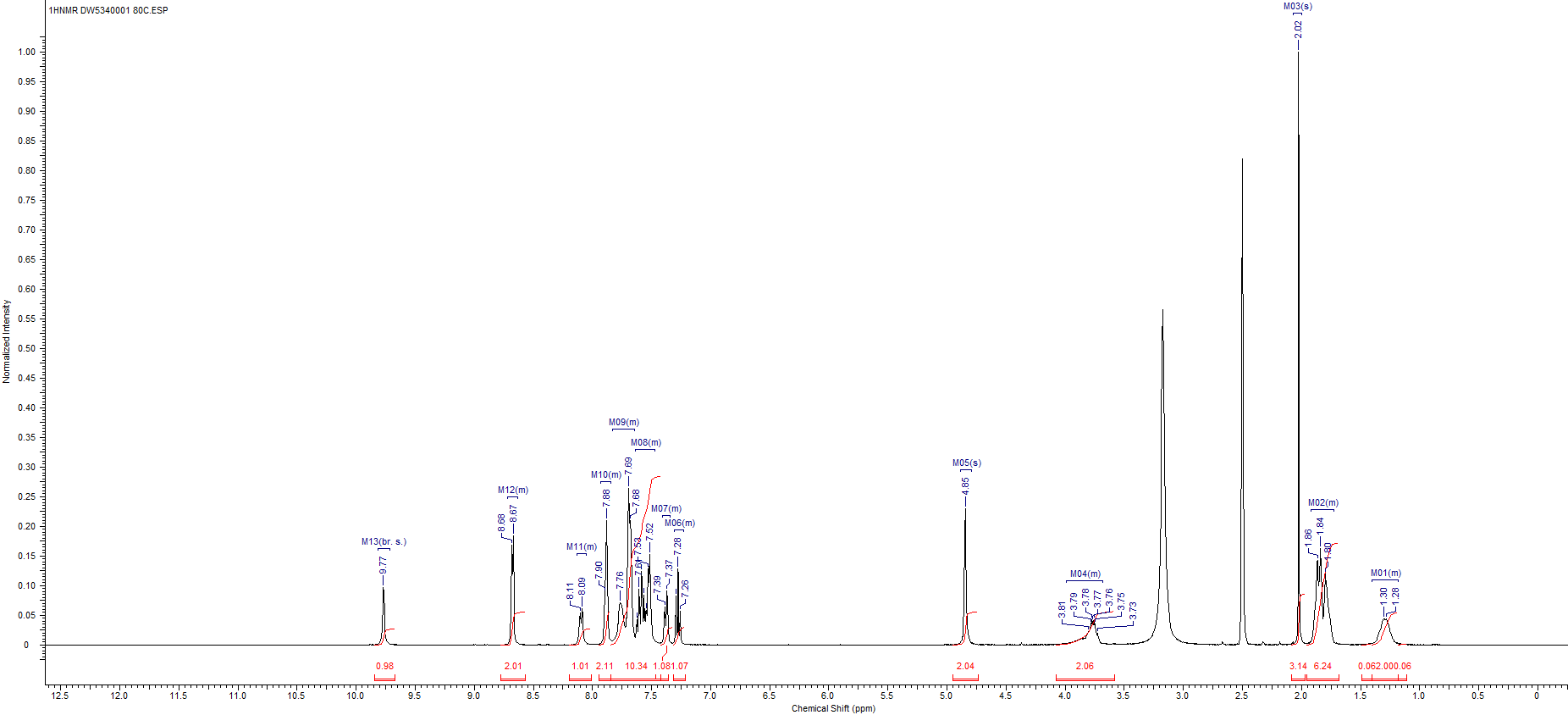
**

**
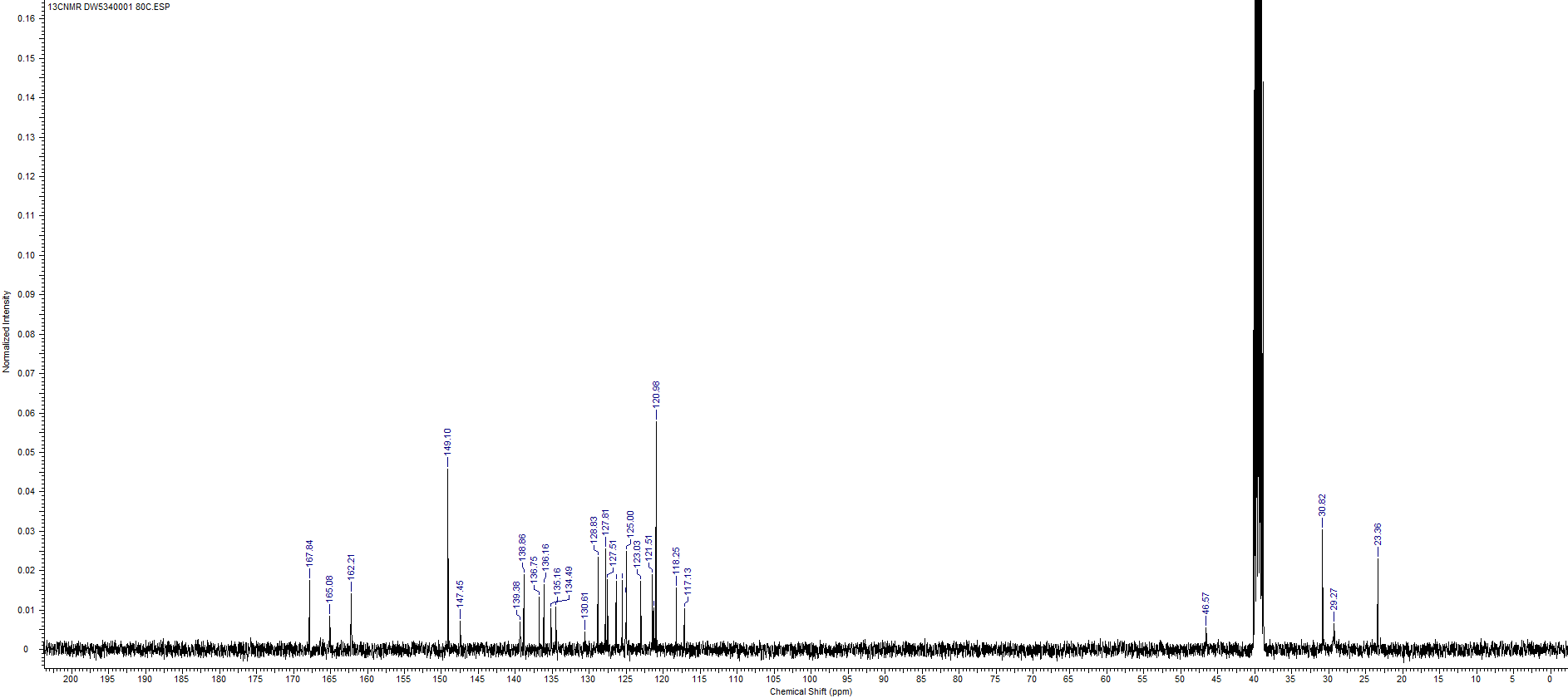
**

**
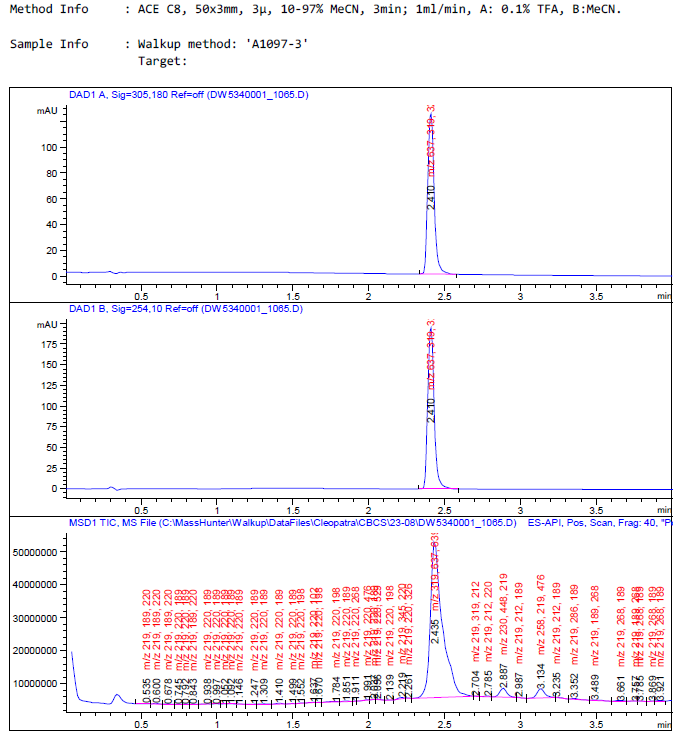

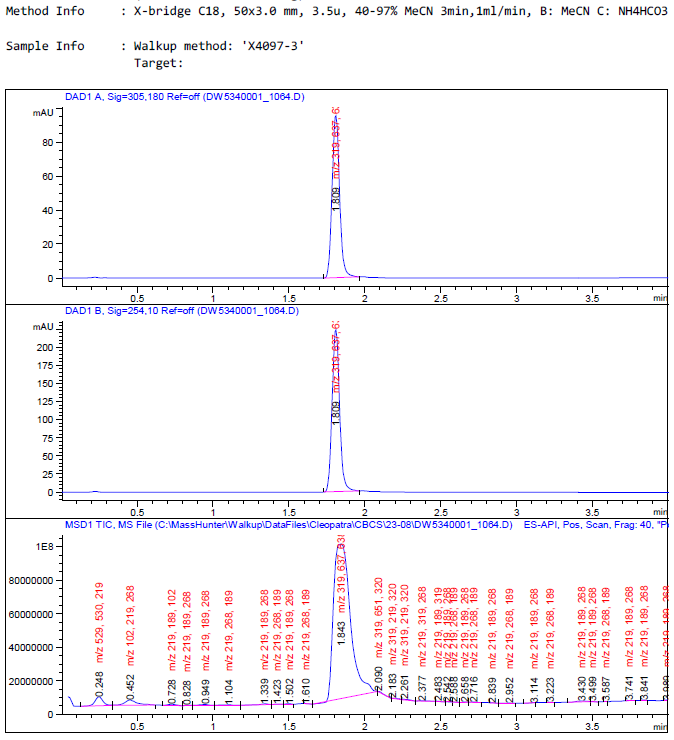
**

**3-Chloro-*N*-((1*r*,4*r*)-4-(3-propionamidobenzamido)cyclohexyl)-*N*-(3-(pyridin-4-yl)benzyl)benzo[*b*]thiophene-2-carboxamide (DJ508501, compound 14)**


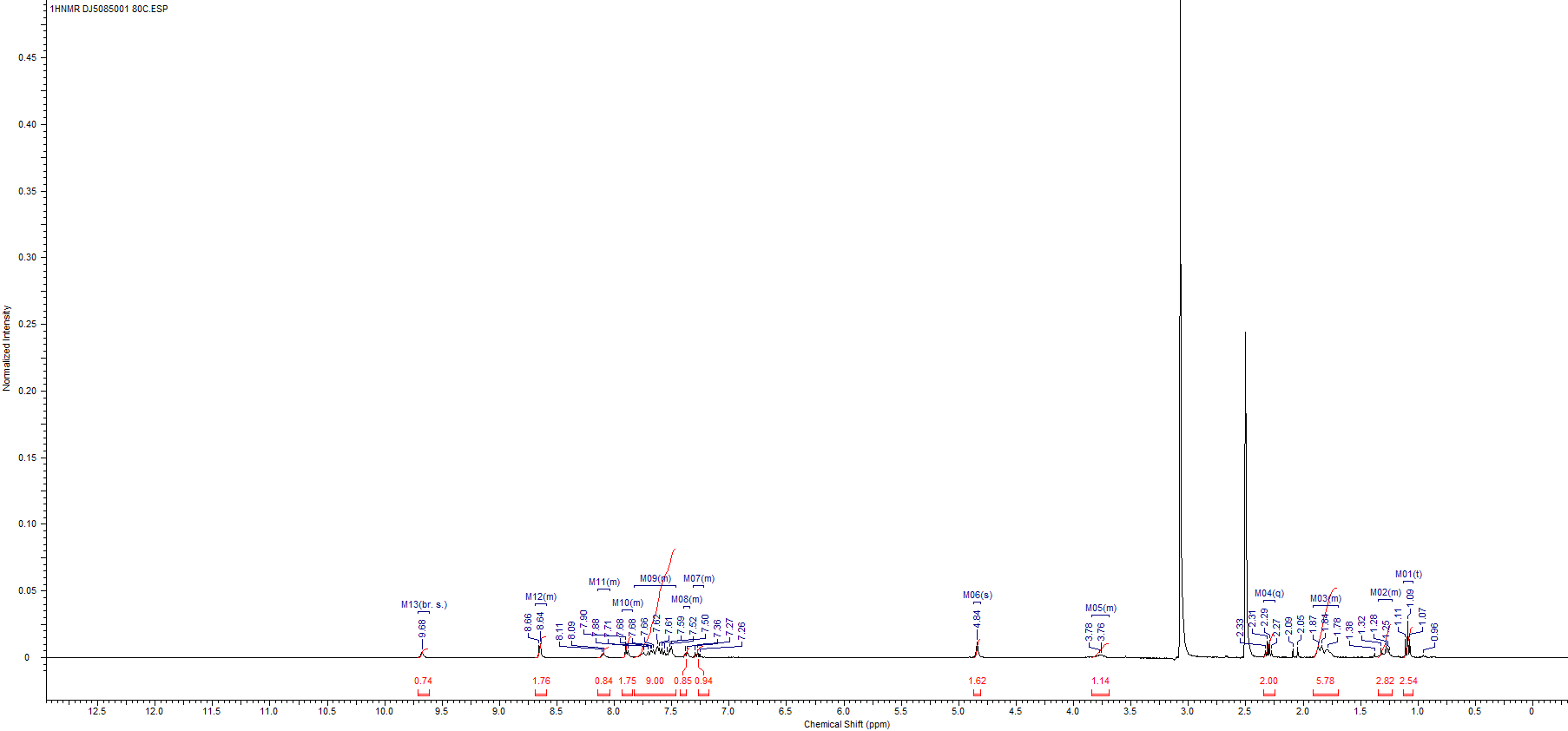


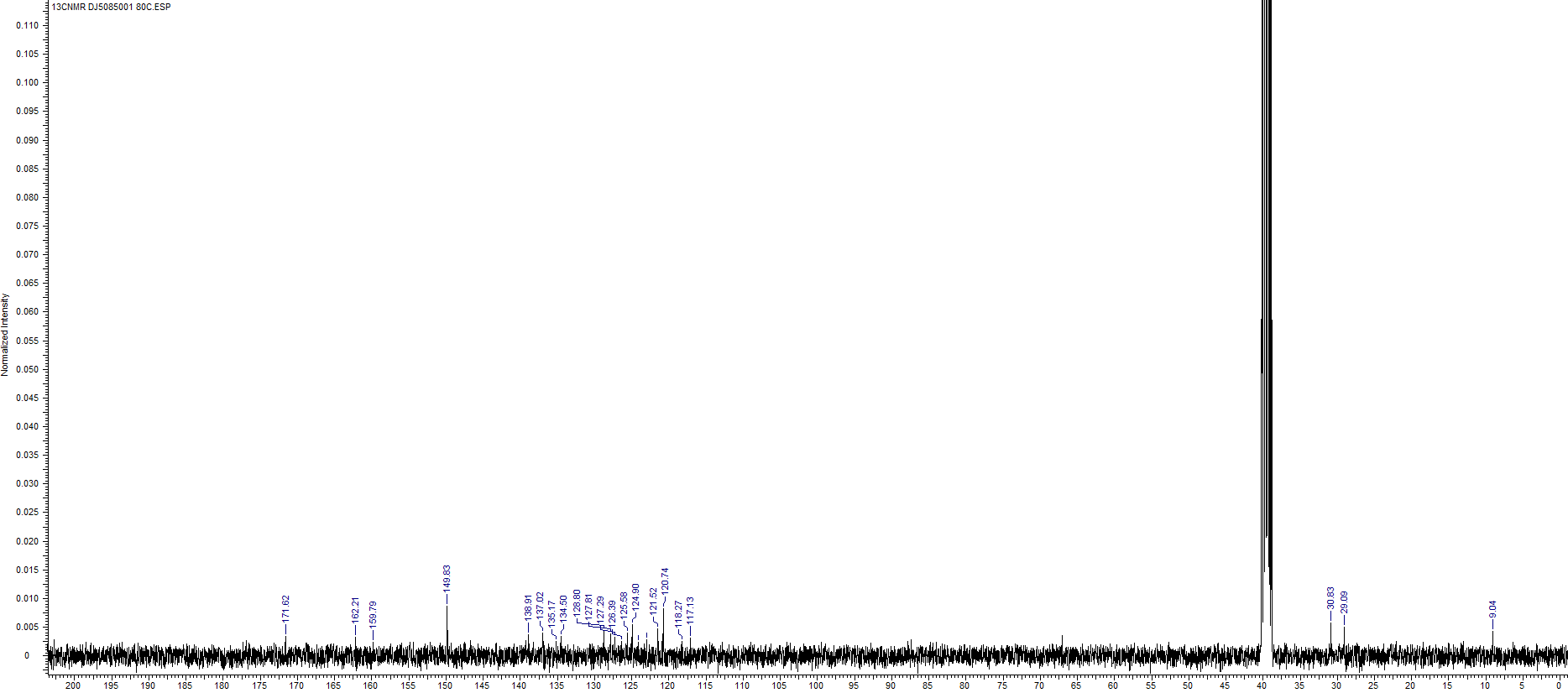


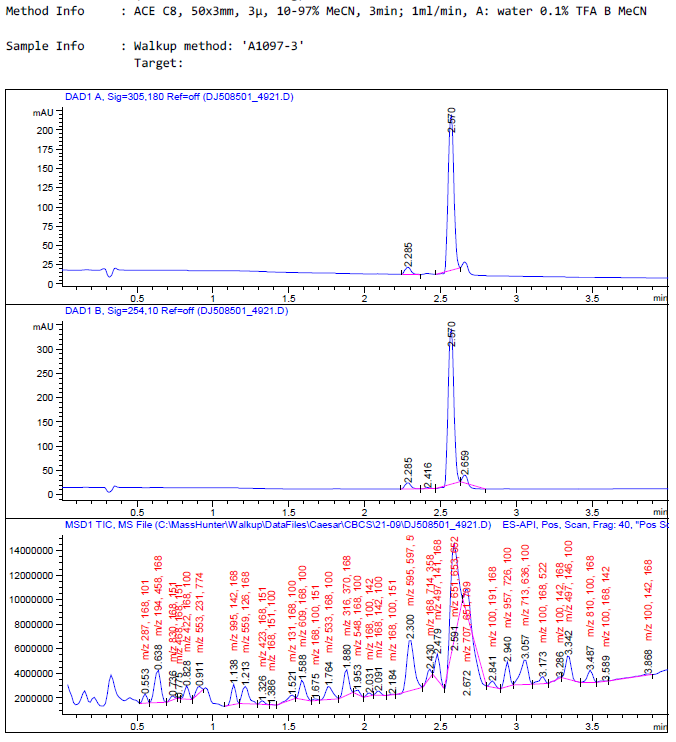

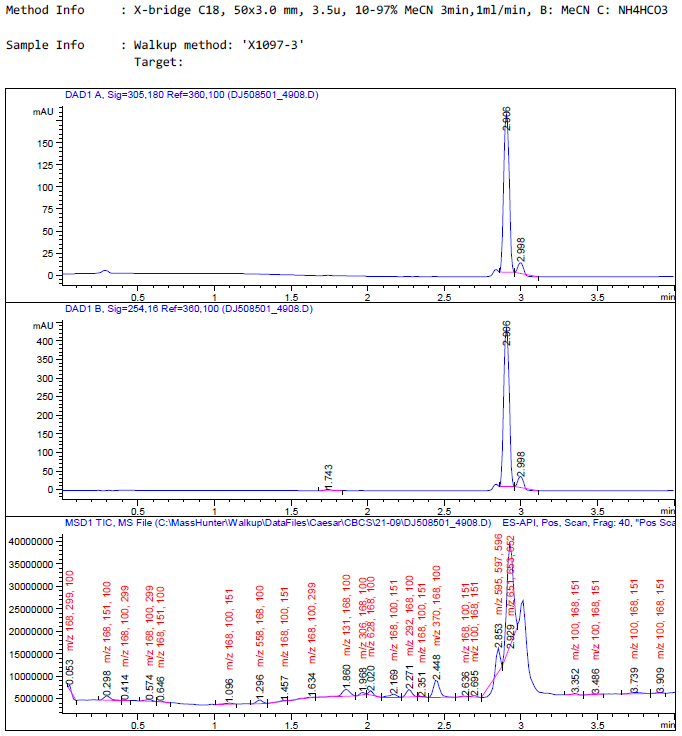


**3-Chloro-*N*-((1*r*,4*r*)-4-(3-isobutyramidobenzamido)cyclohexyl)-*N*-(3-(pyridin-4-yl)benzyl)benzo[*b*]thiophene-2-carboxamide (DJ508401, compound 15)**


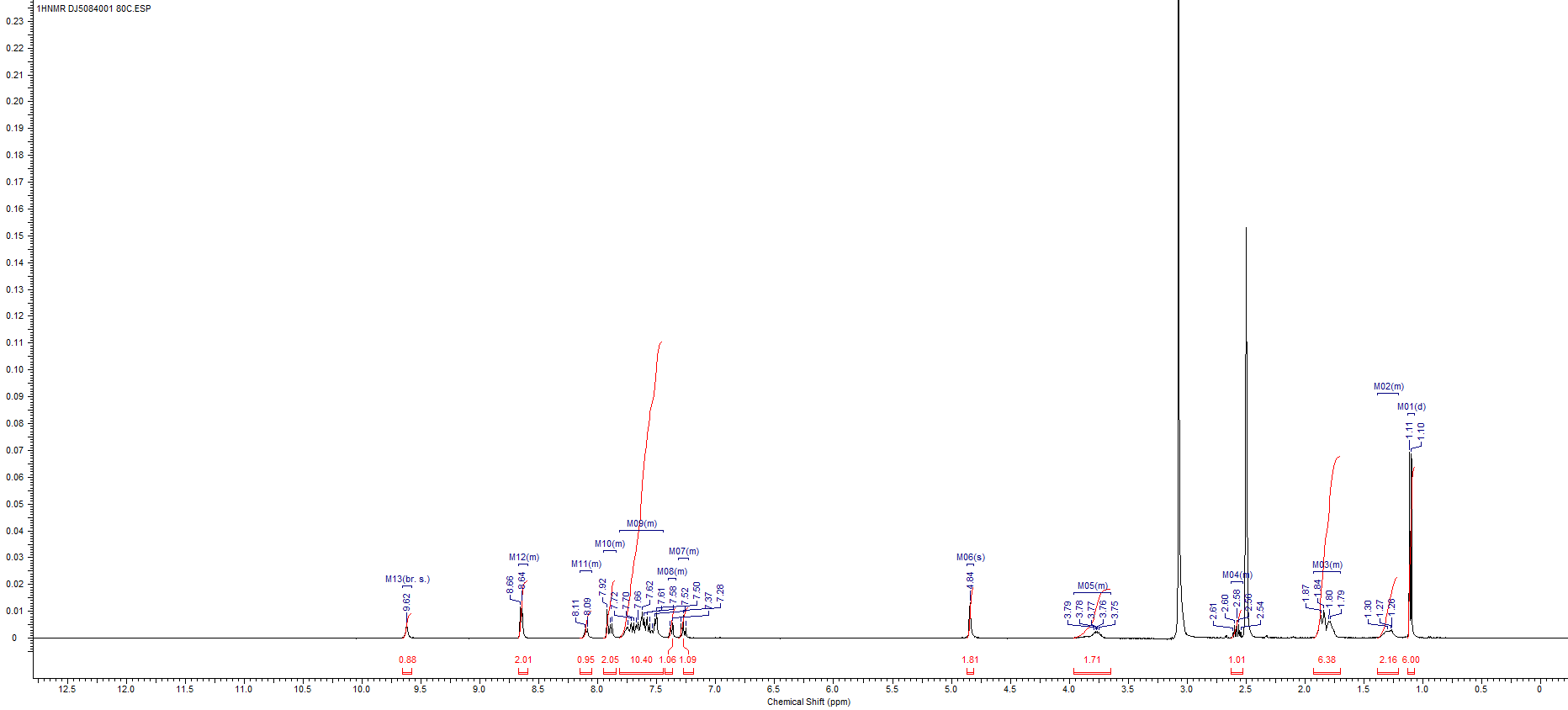


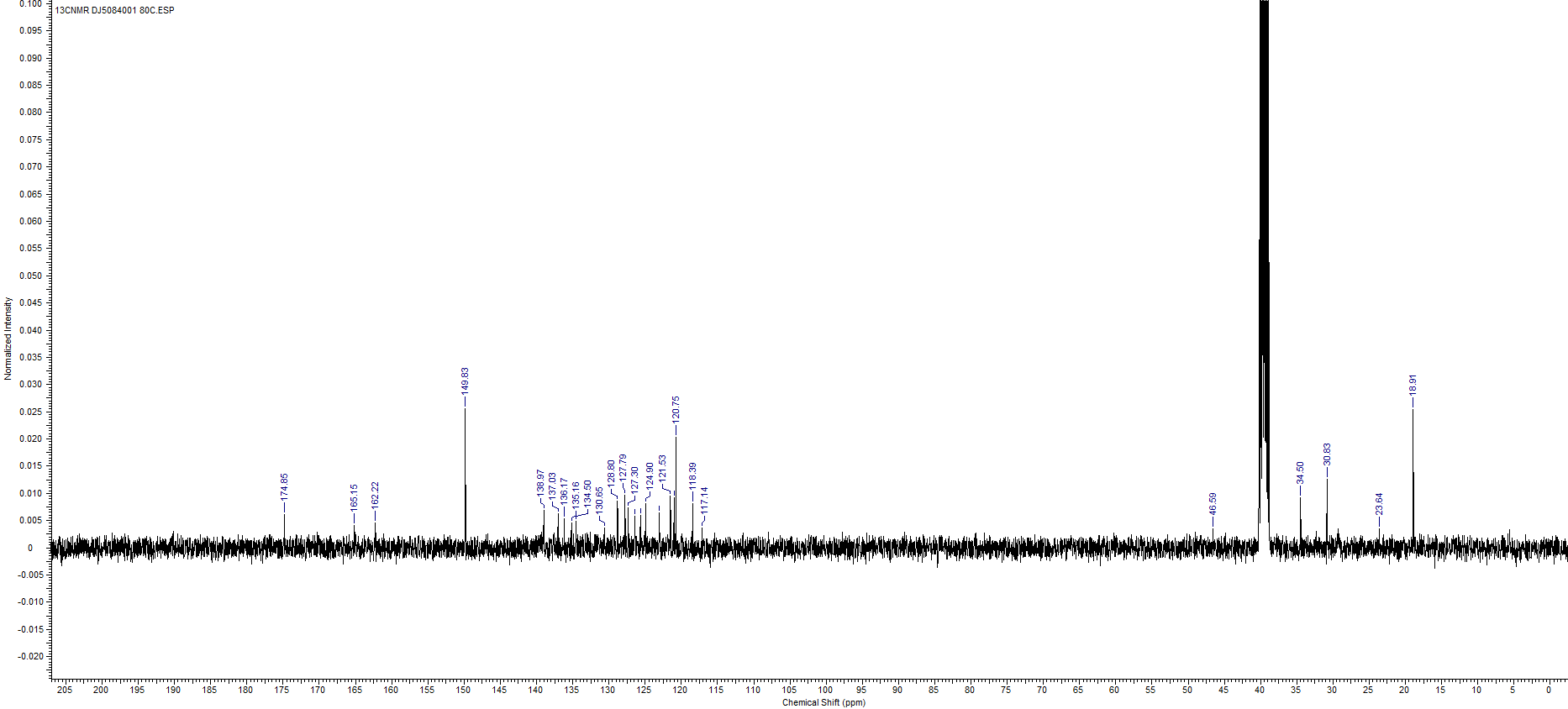


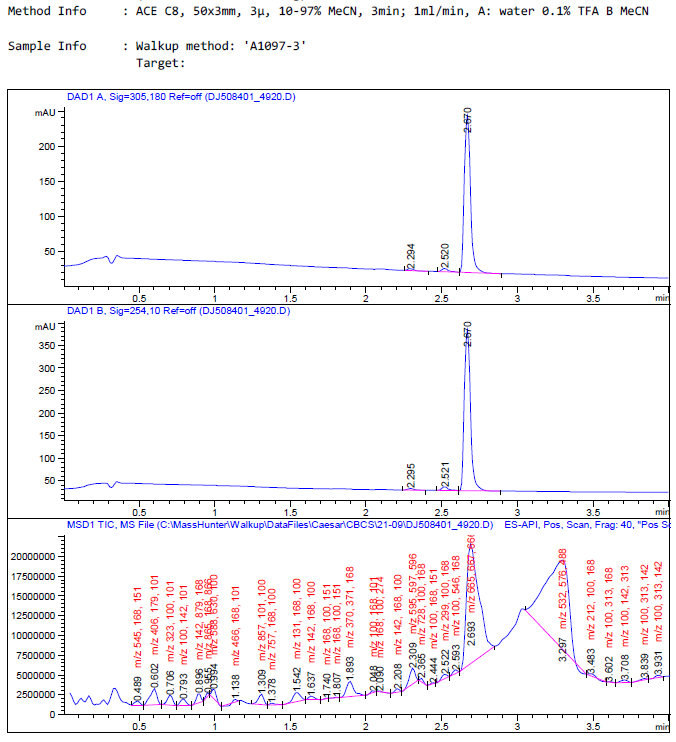

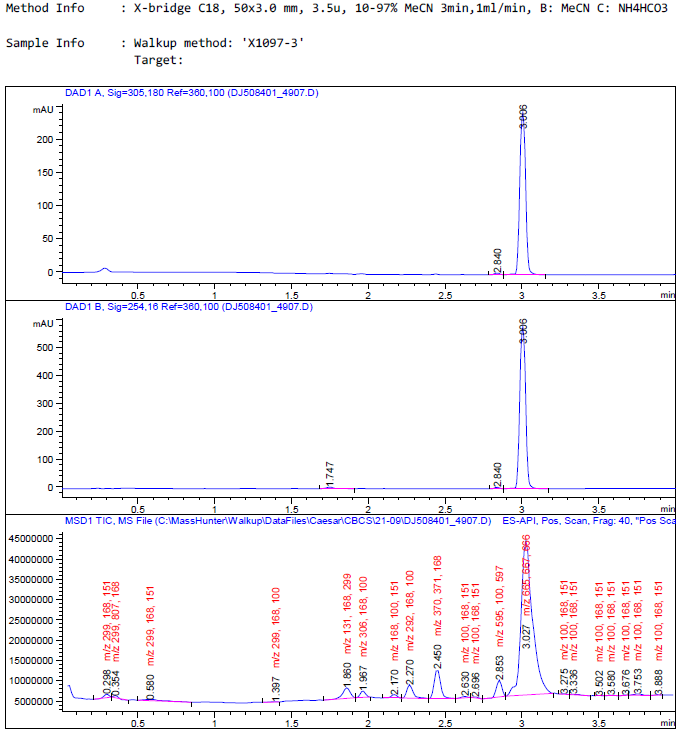


***N*-(3-(((1*r*,4*r*)-4-(3-Chloro-*N*-(3-(pyridin-4-yl)benzyl)benzo[*b*]thiophene-2-carboxamido)cyclohexyl)carbamoyl)phenyl)-1-methylpiperidine-4-carboxamide (DJ508701, compound 17)**


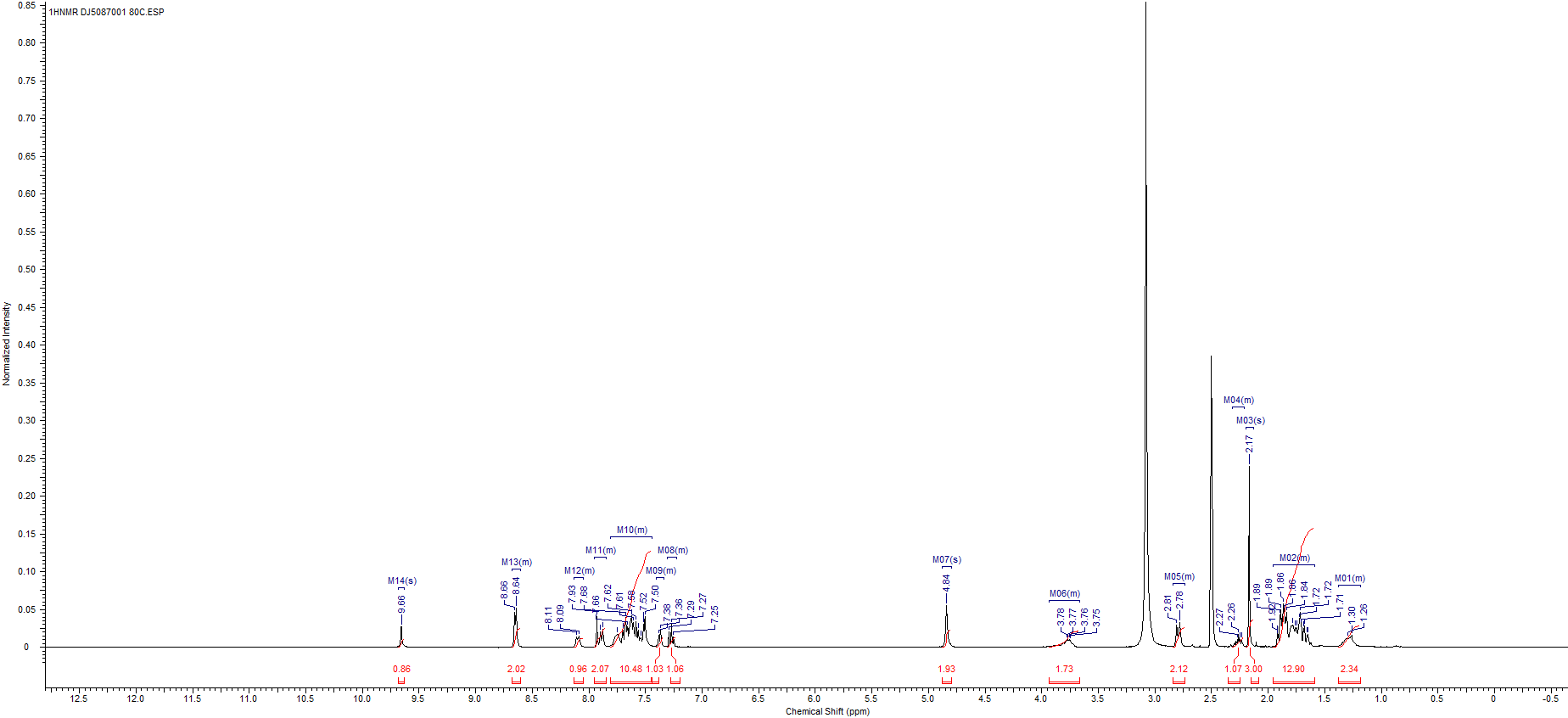


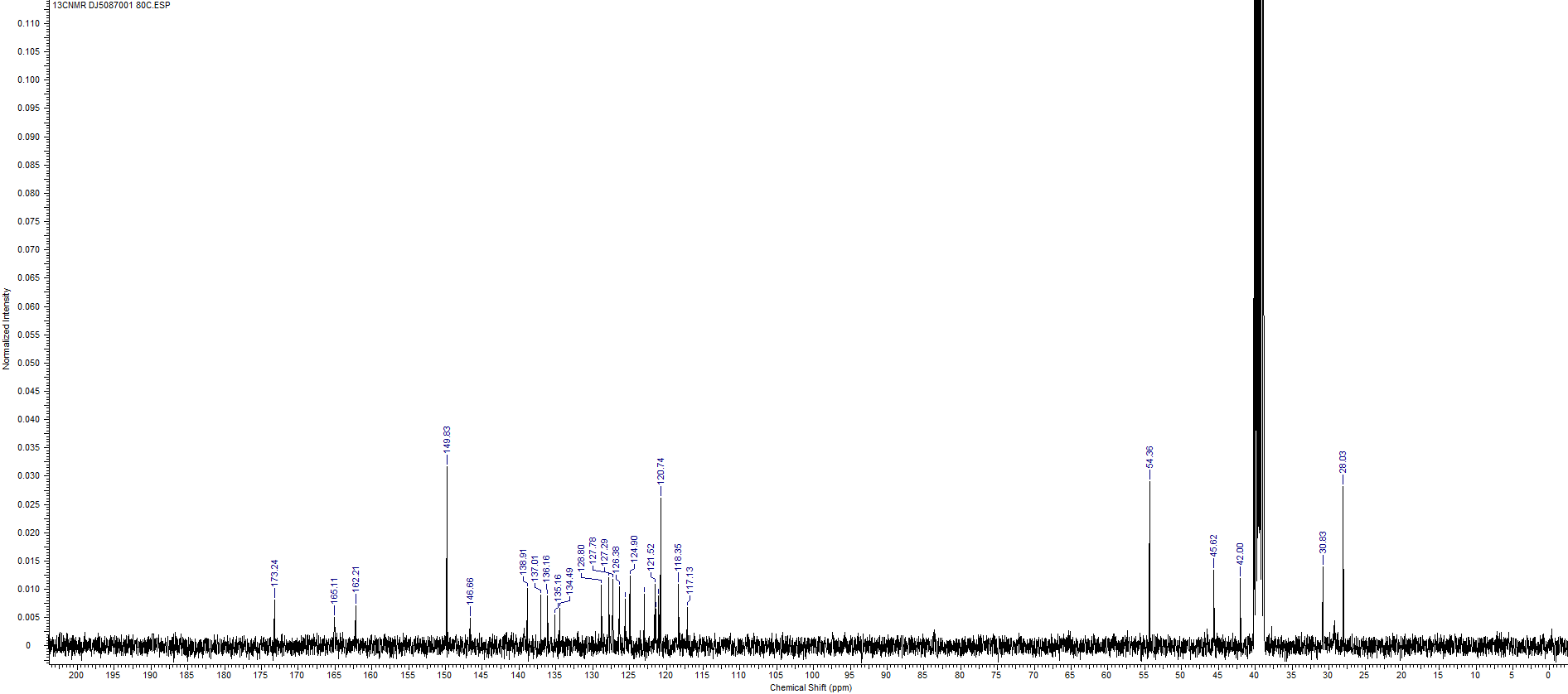


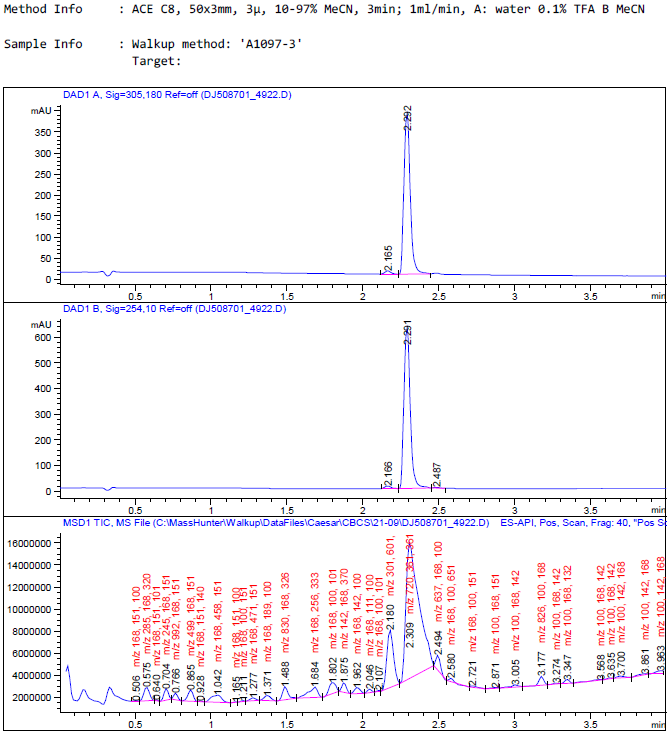

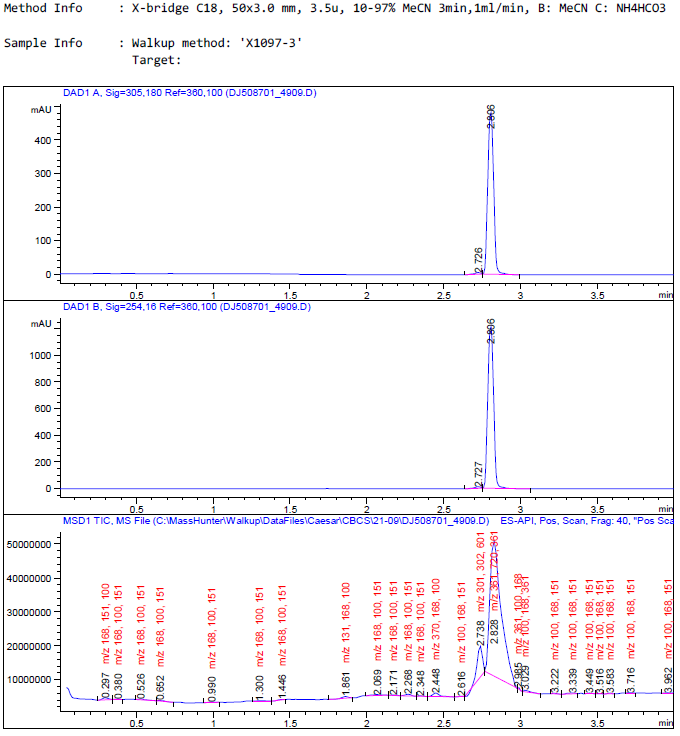


**3-Chloro-*N*-((1*r*,4*r*)-4-(3-(2-(4-methylpiperazin-1-yl)acetamido)benzamido)cyclohexyl)-*N*-(3-(pyridin-4-yl)benzyl)benzo[*b*]thiophene-2-carboxamide (DQ090601, compound 18)**


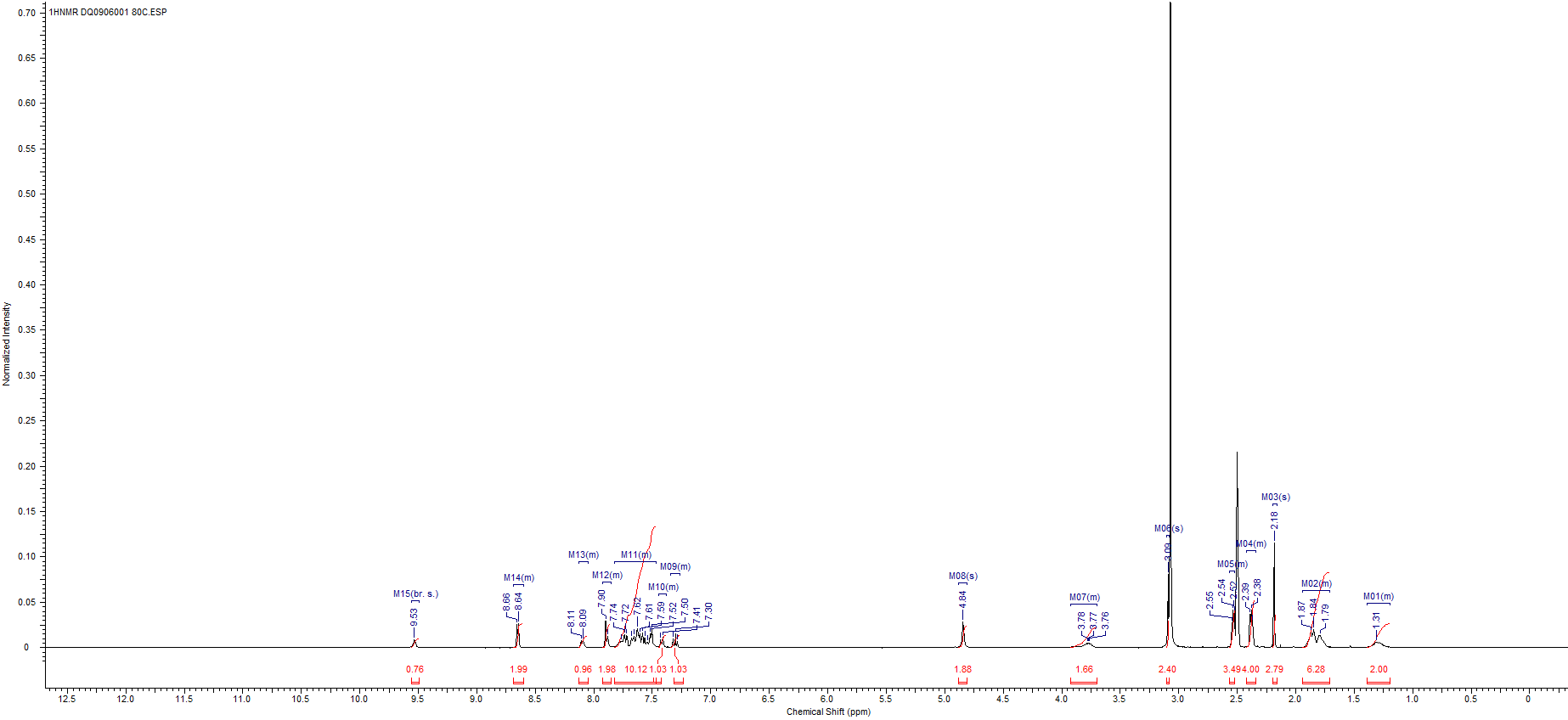


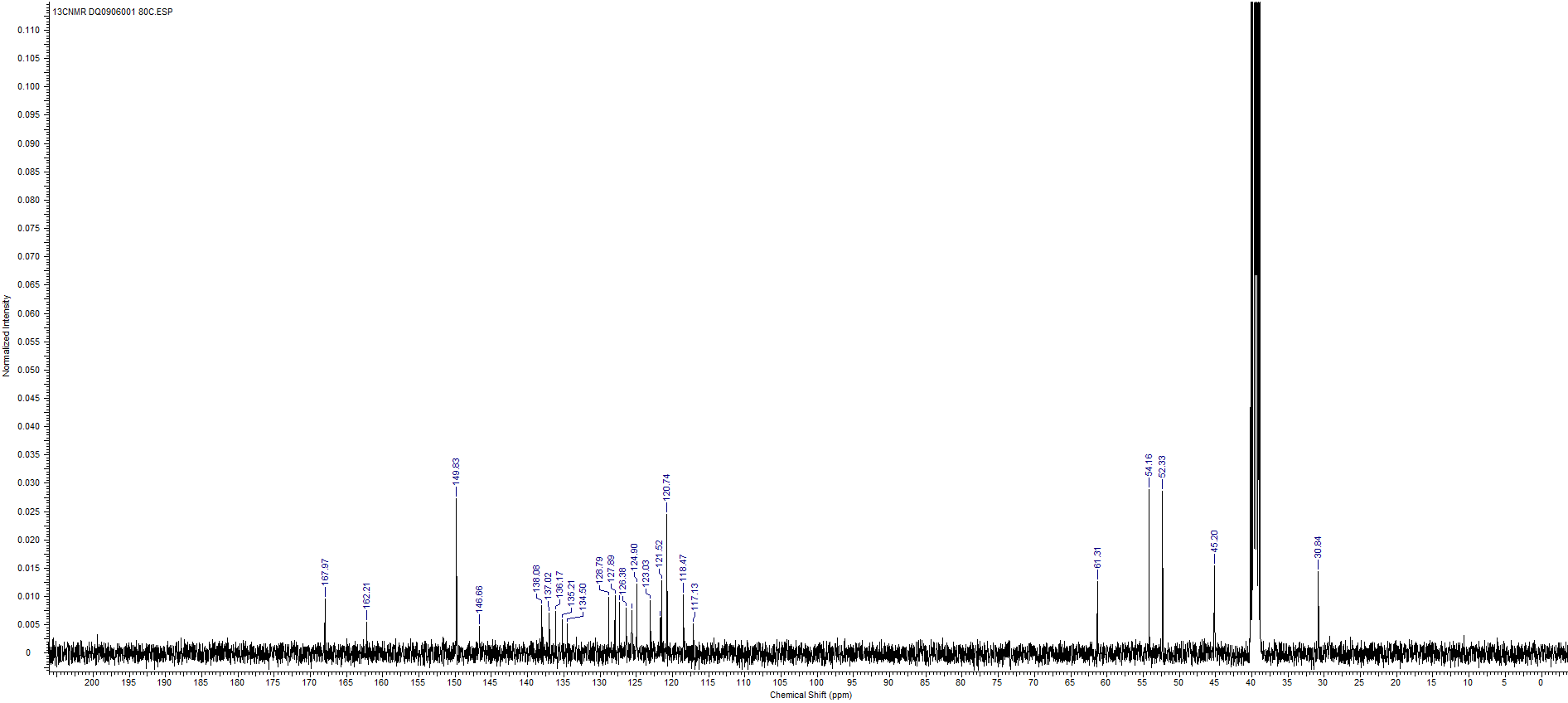


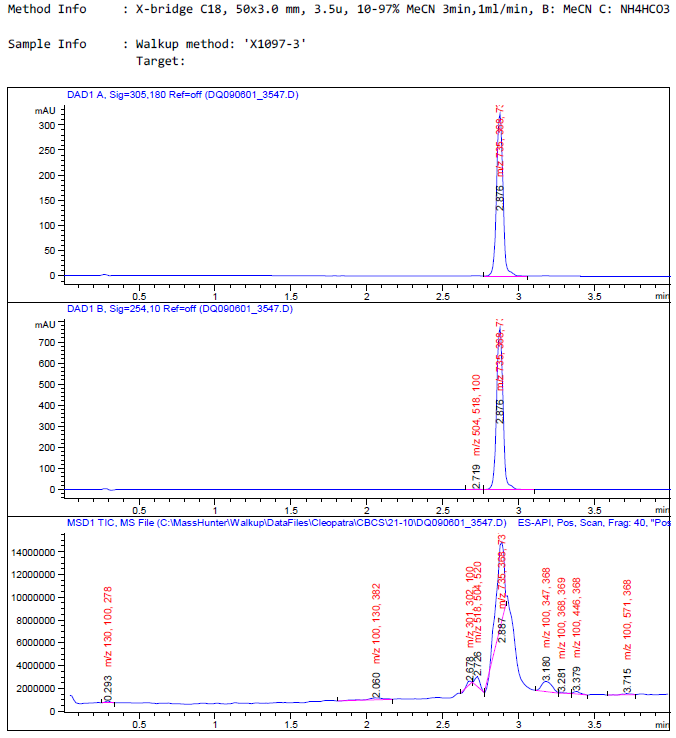


**3-Chloro-*N*-((1*r*,4*r*)-4-(3-(2-morpholinoacetamido)benzamido)cyclohexyl)-*N*-(3-(pyridin-4-yl)benzyl)benzo[*b*]thiophene-2-carboxamide (DQ090801, compound 19)**


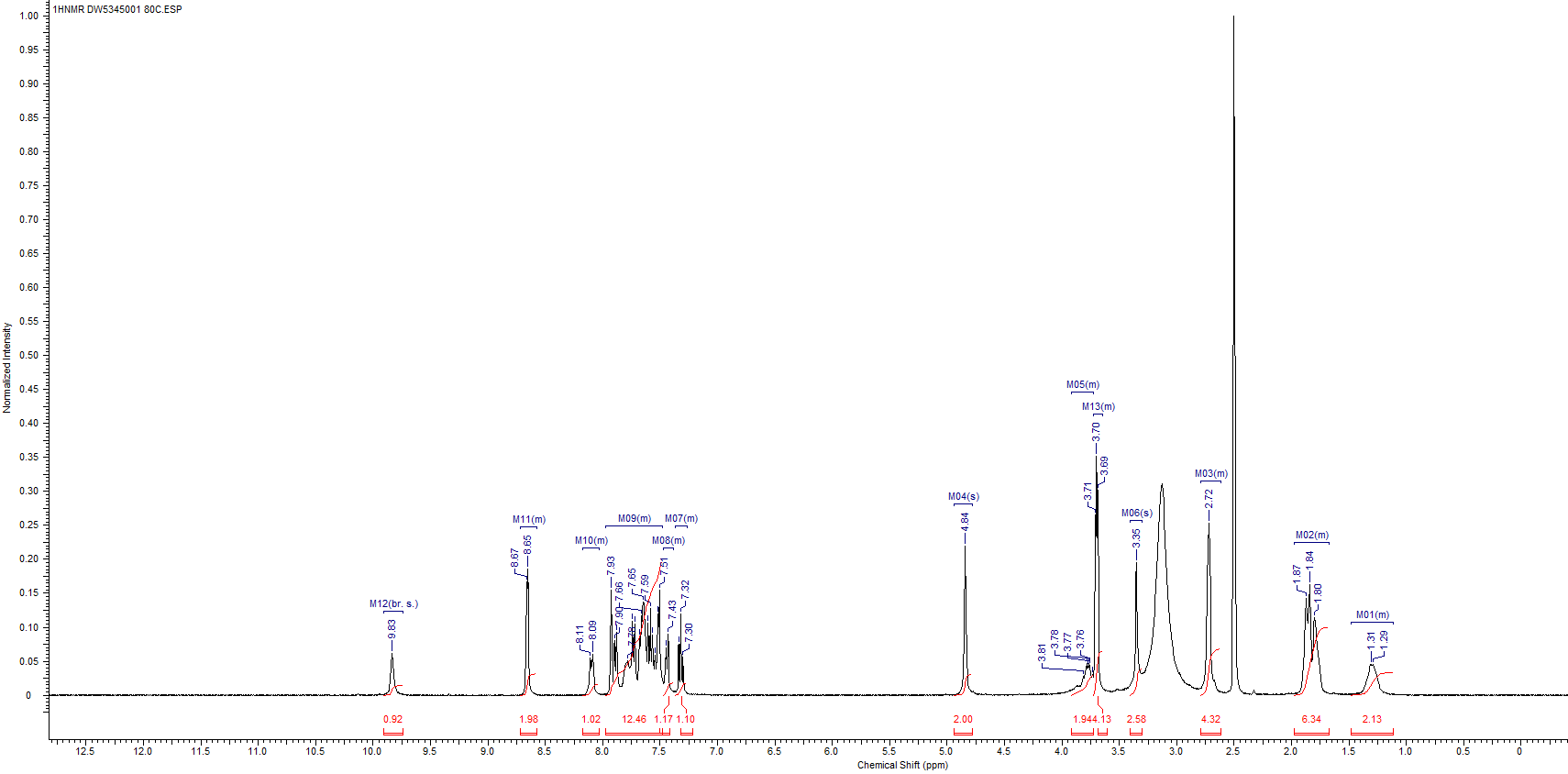


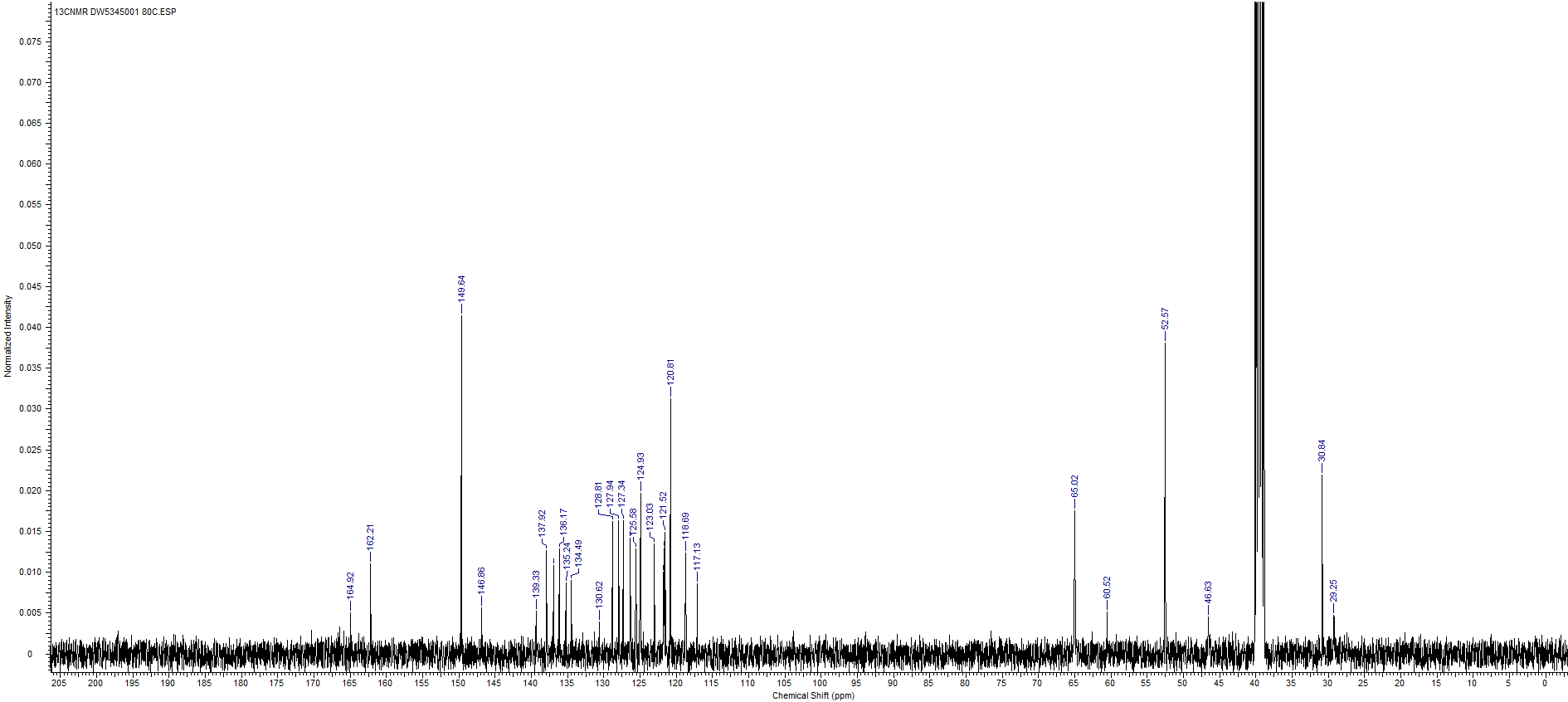


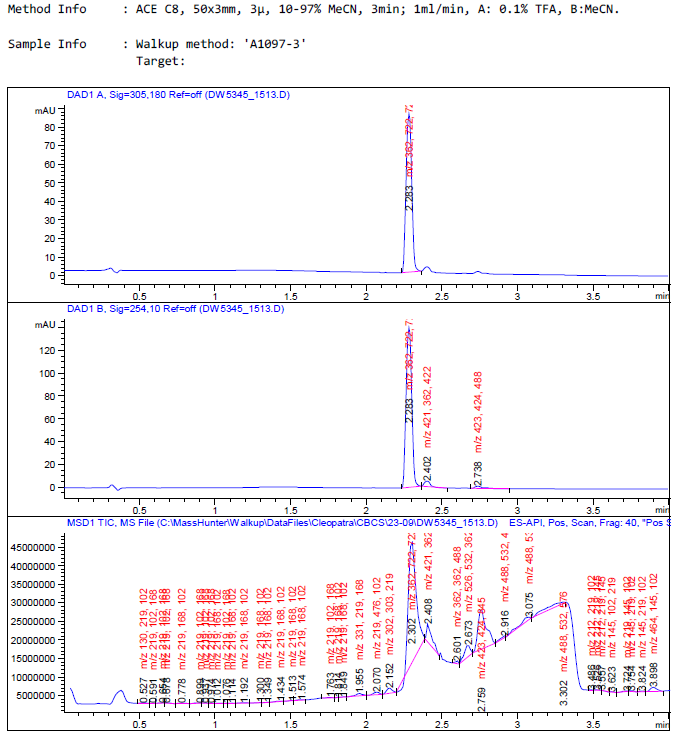

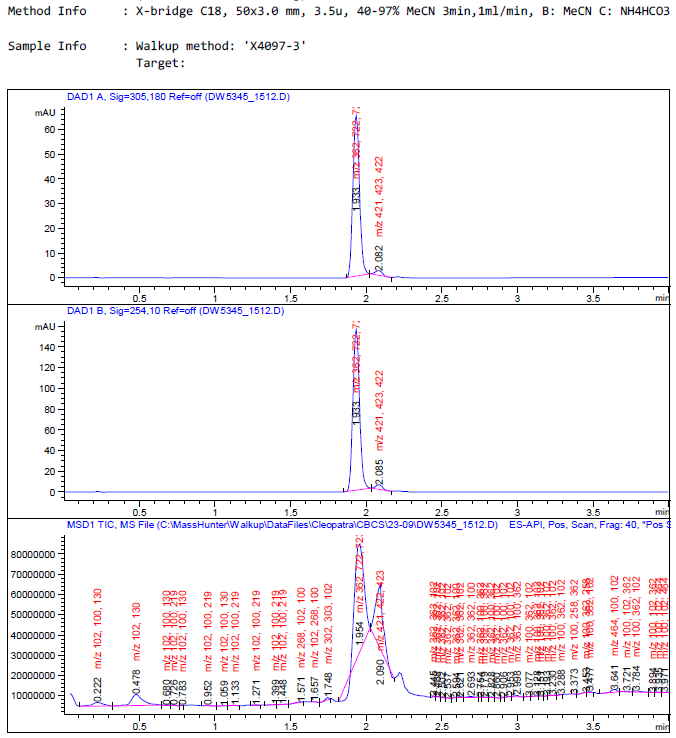


**3-Chloro-*N*-((1*r*,4*r*)-4-(3-chloro-*N*-(3-(pyridin-4-yl)benzyl)benzo[*b*]thiophene-2-carboxamido)cyclohexyl)isonicotinamide (DQ090001, compound 21)**


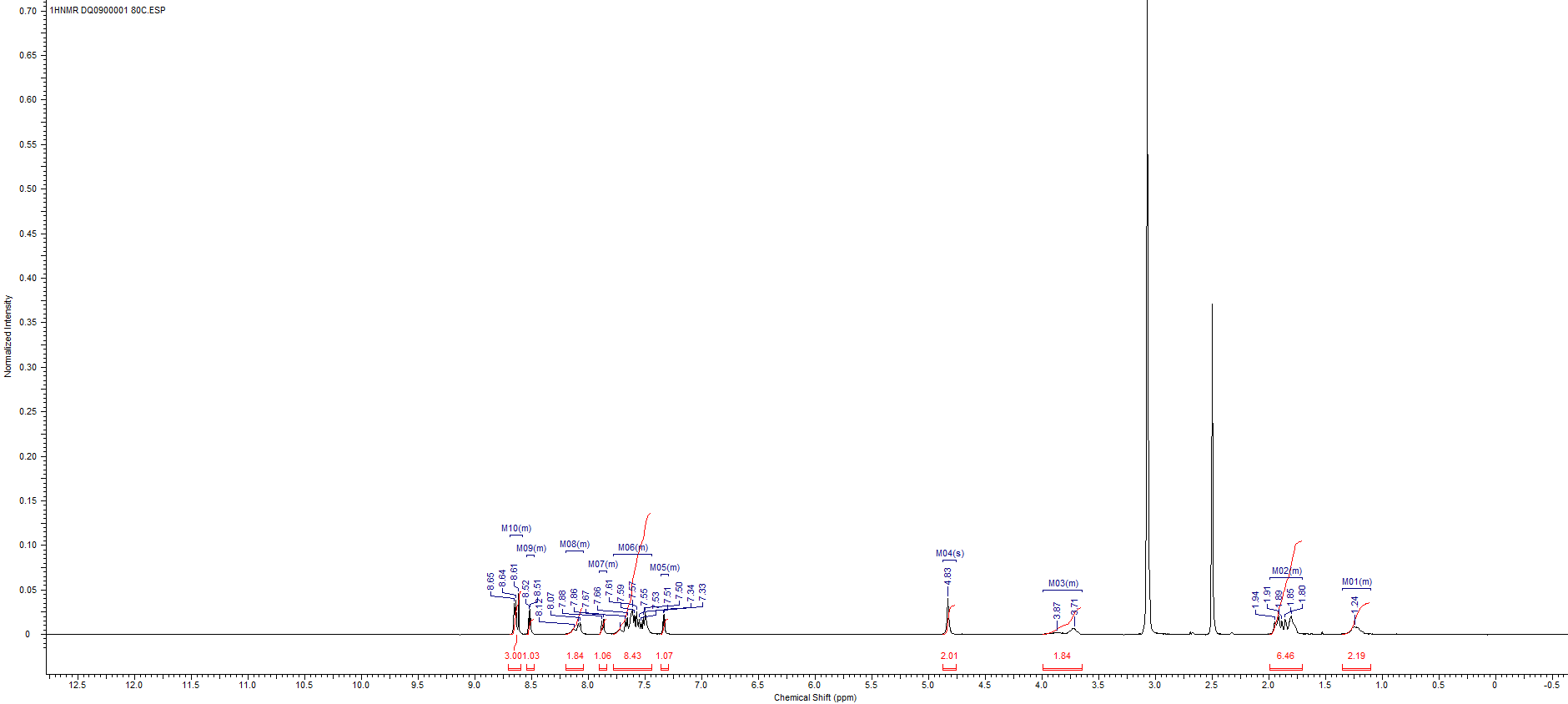


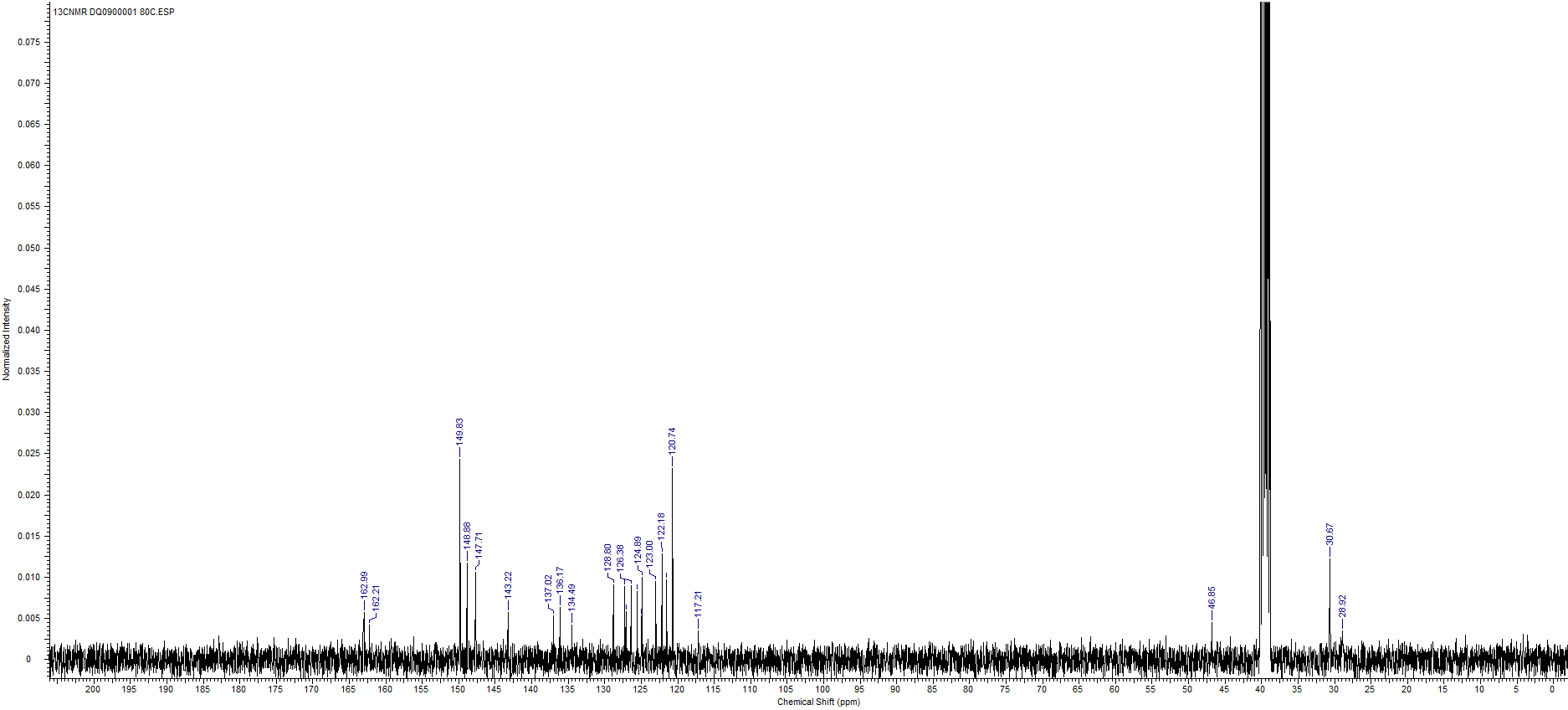


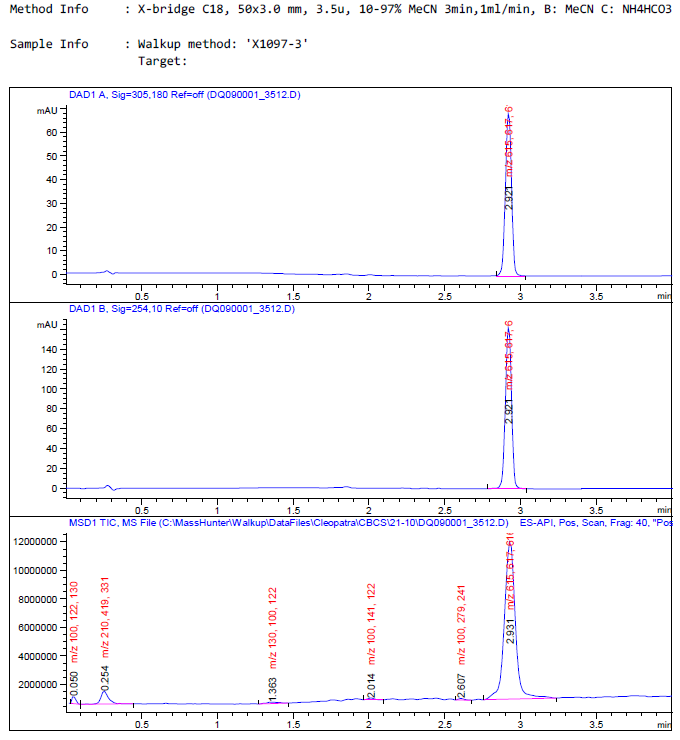


**6-Amino-*N*-((1*r*,4*r*)-4-(3-chloro-*N*-(3-(pyridin-4-yl)benzyl)benzo[*b*]thiophene-2-carboxamido)cyclohexyl)pyridazine-3-carboxamide (DQ090901, compound 23)**


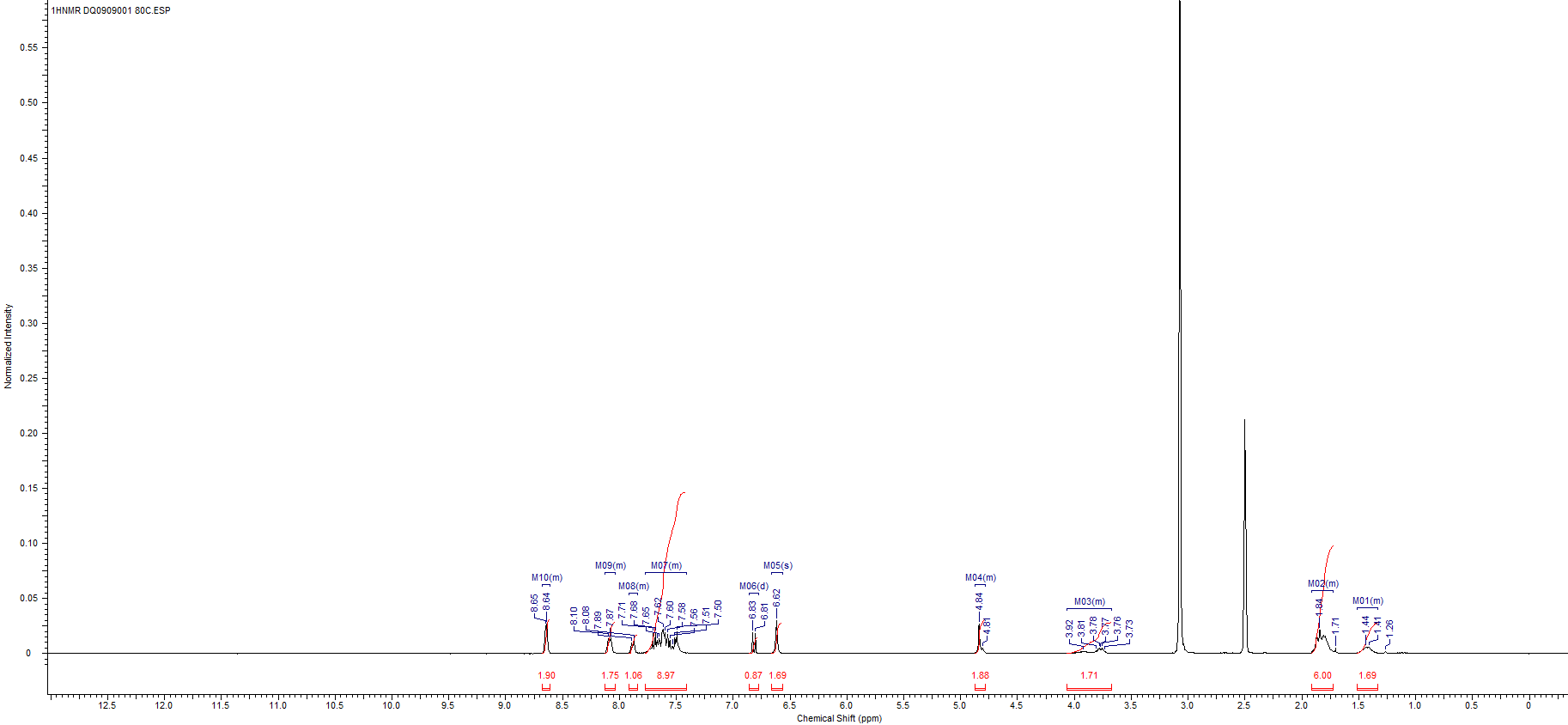


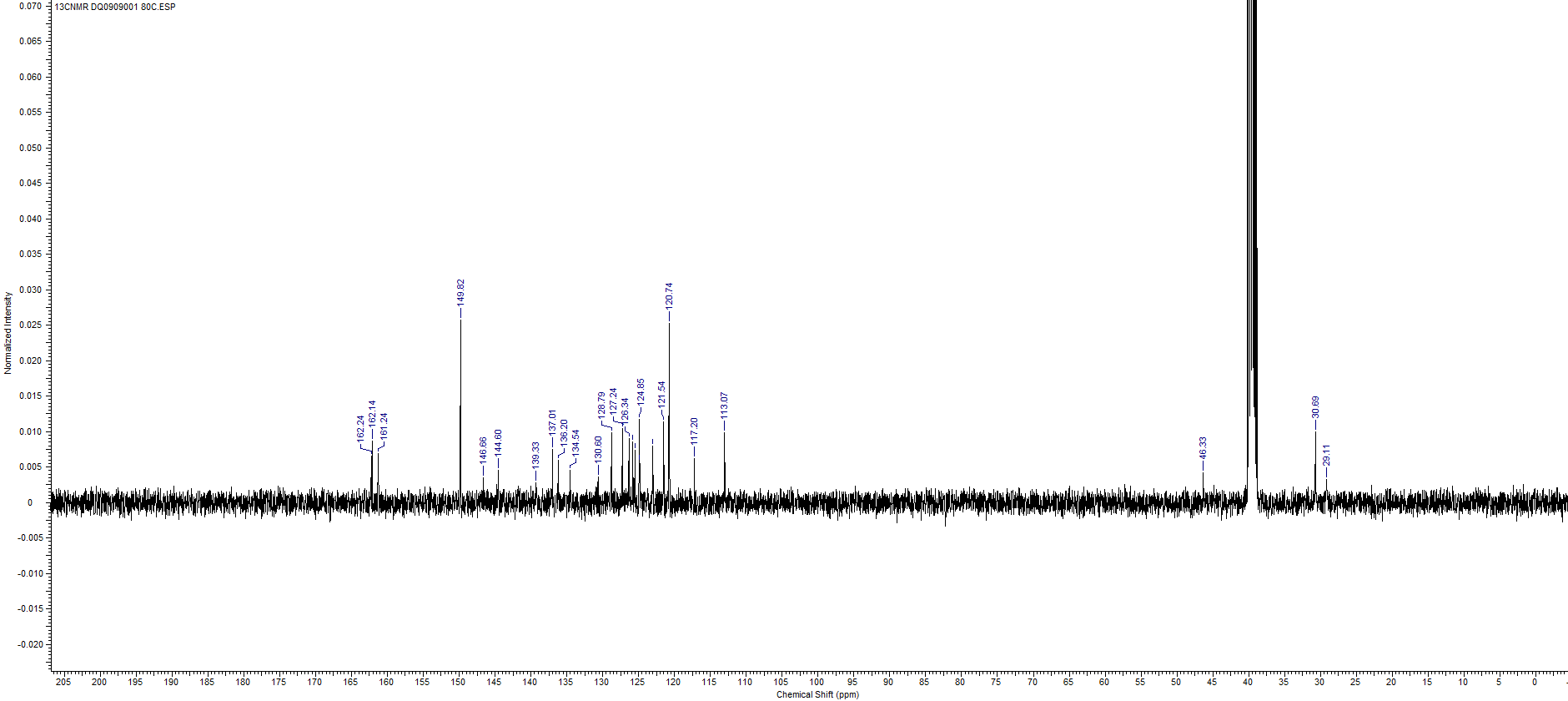


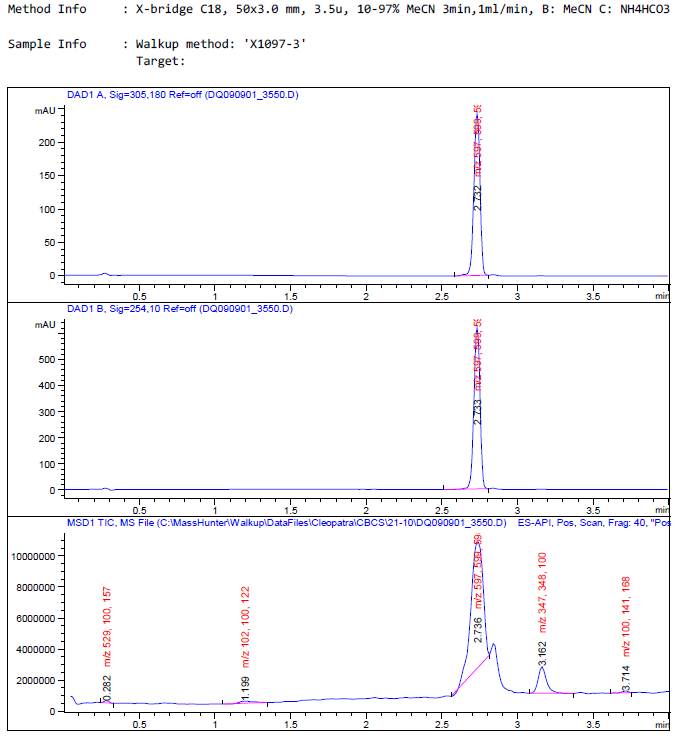


***N*-(1-(2-([1,1'-Biphenyl]-3-ylamino)-1-(benzofuran-2-yl)-2-oxoethyl)piperidin-4-yl)-3-acetamidobenzamide (DJ509001, compound 24)**


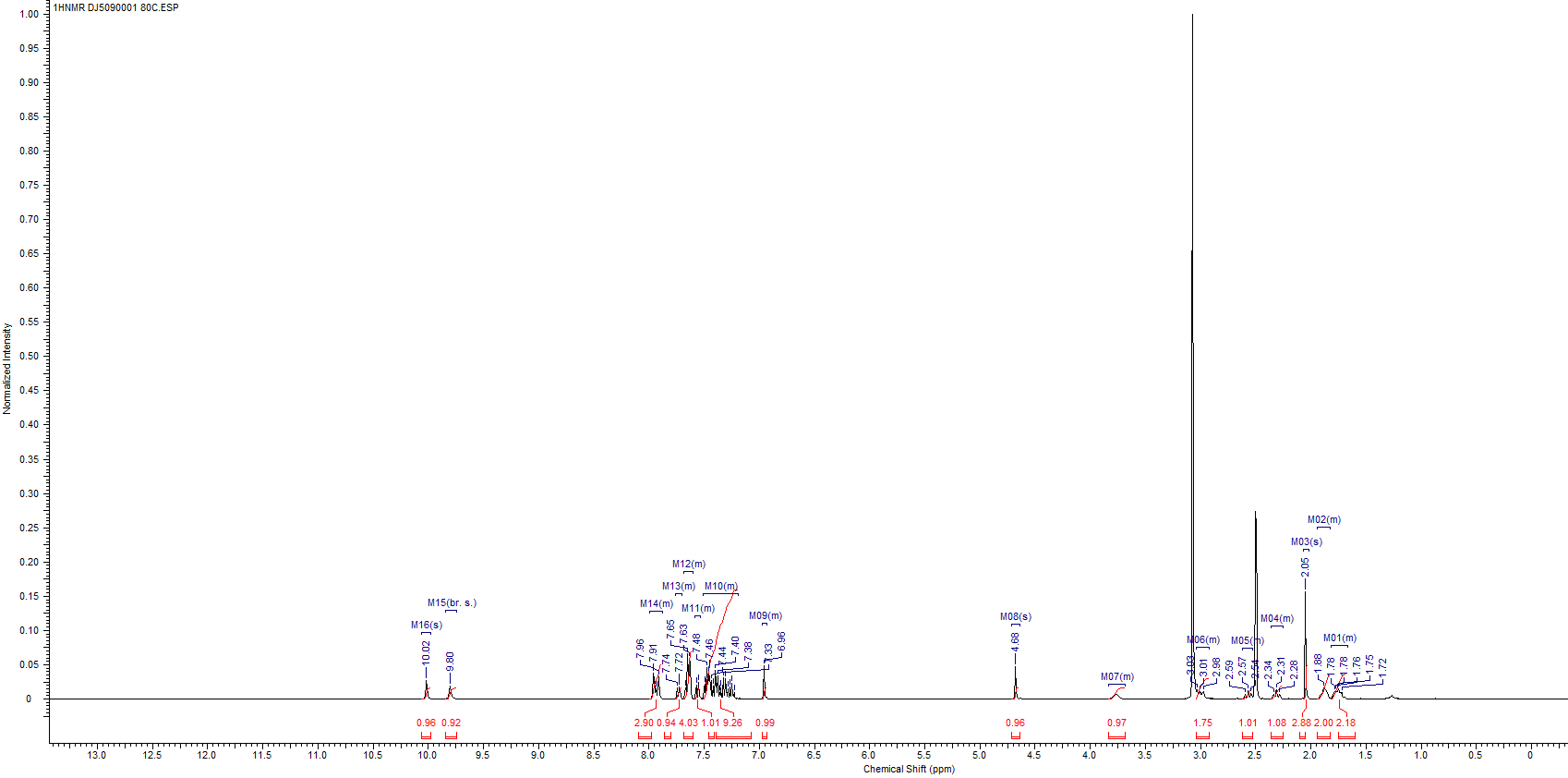


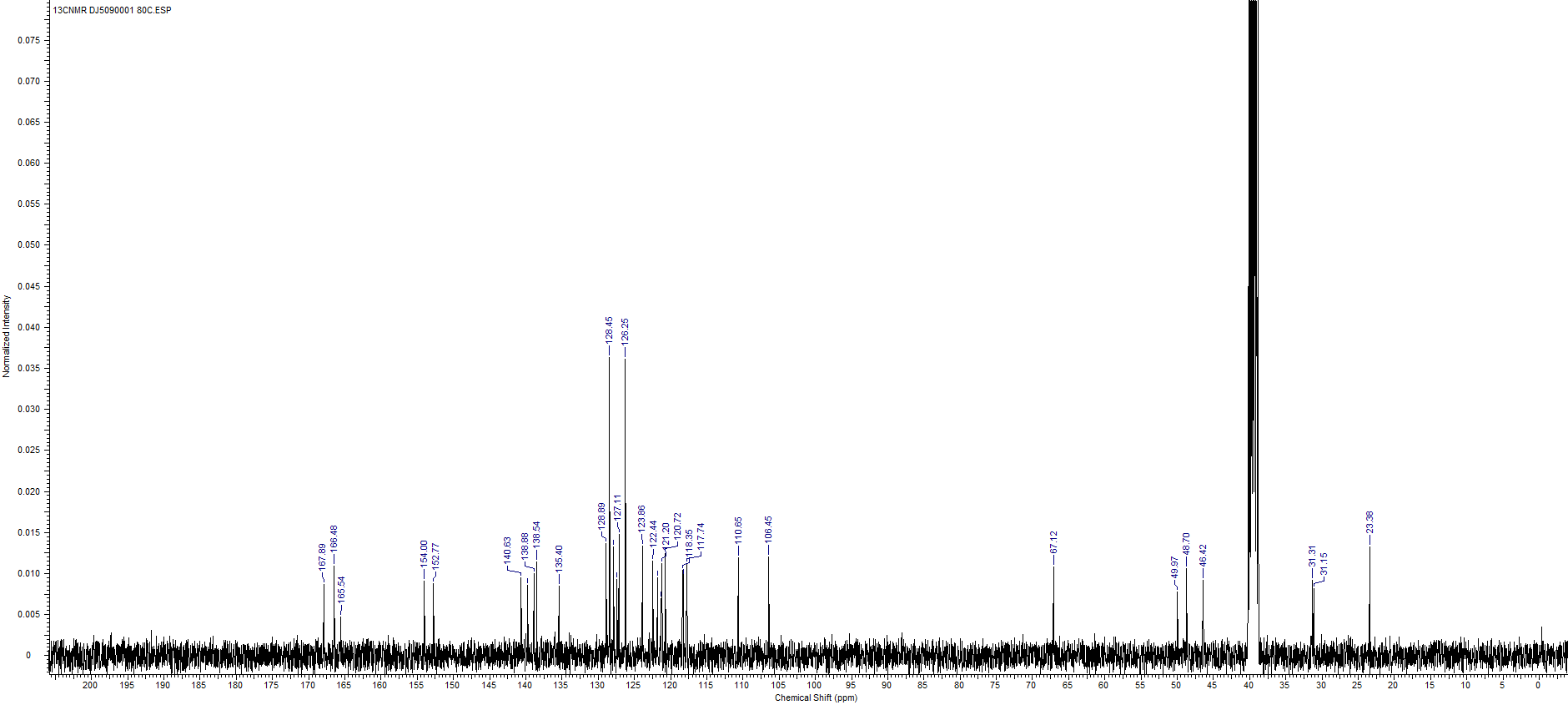


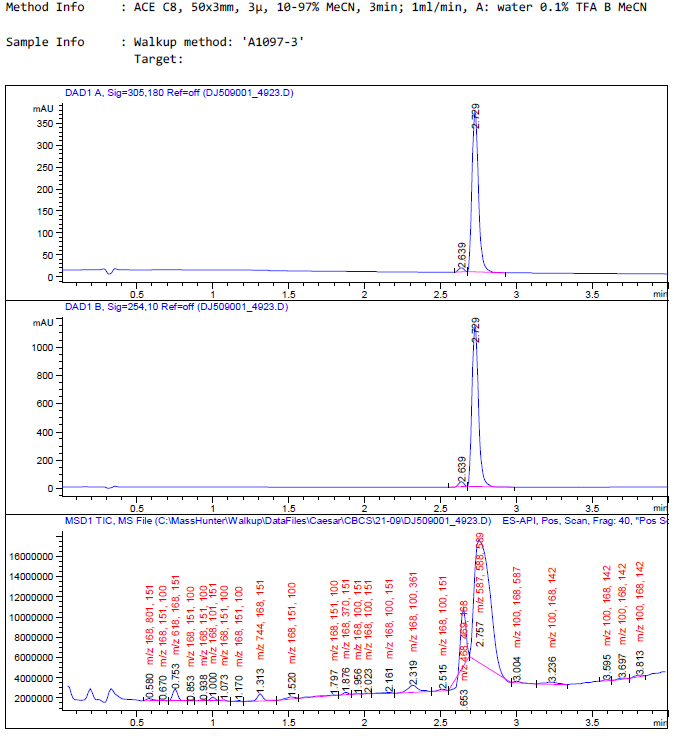

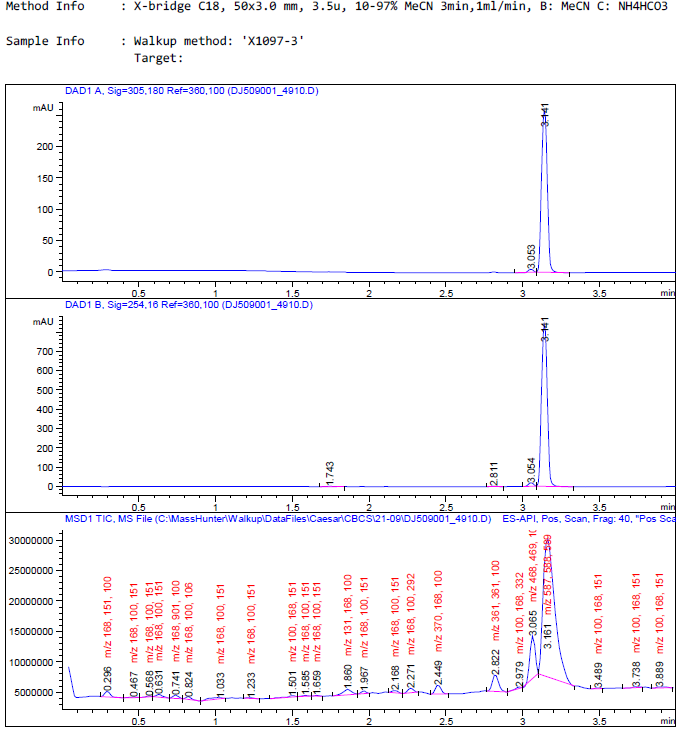


***N*-(4-(3-Chloro-N-(4-(pyridin-3-yl)benzyl)benzo[*b*]thiophene-2-carboxamido)cyclohexyl)furan-2-carboxamide (MRG26, compound 52)**


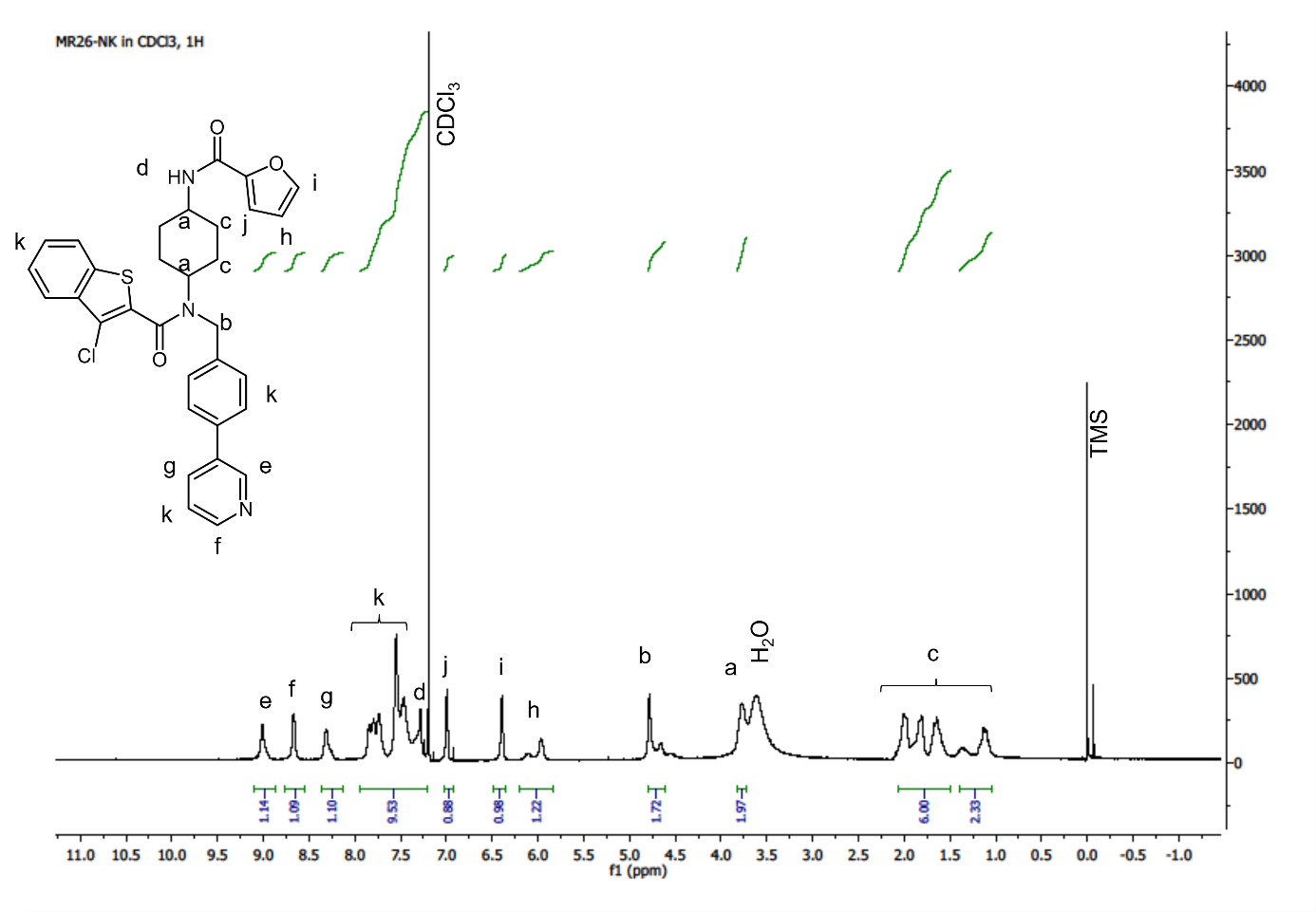


**References**

1 Kinsolving, J. *et al.* A Putative Frizzled 7-Targeting Compound Acts as a Firefly Luciferase Inhibitor. *J Med Chem* **67**, 22332-22341, doi:10.1021/acs.jmedchem.4c02766 (2024).

2 Vorrink, S. U. *et al.* Endogenous and xenobiotic metabolic stability of primary human hepatocytes in long-term 3D spheroid cultures revealed by a combination of targeted and untargeted metabolomics. *Faseb J* **31**, 2696-2708, doi:10.1096/fj.201601375R (2017).

3 Oliva-Vilarnau, N., Vorrink, S. U., Ingelman-Sundberg, M. & Lauschke, V. M. A 3D Cell Culture Model Identifies Wnt/beta-Catenin Mediated Inhibition of p53 as a Critical Step during Human Hepatocyte Regeneration. *Adv Sci (Weinh)* **7**, 2000248, doi:10.1002/advs.202000248 (2020).

4 Gratz, L., Voss, J. H. & Schulte, G. Class-Wide Analysis of Frizzled-Dishevelled Interactions Using BRET Biosensors Reveals Functional Differences among Receptor Paralogs. *ACS Sens* **9**, 4626-4636, doi:10.1021/acssensors.4c00806 (2024).
